# Harnessing Large‐Scale Multi‐Omics Data for Risk Prediction and Deep Phenotyping of Valvular Heart Diseases in the General Population

**DOI:** 10.1002/advs.76345

**Published:** 2026-07-06

**Authors:** Zhihao Jiang, Yang Liu, Mingyu Song, Ning Chen, Canqing Yu, Jun Lv, Eric Yuk Fai Wan, Lu Qi, Liming Li, Dianjianyi Sun, Bang Zheng

**Affiliations:** ^1^ Department of Epidemiology and Biostatistics School of Public Health Peking University Beijing China; ^2^ Peking University Center for Public Health and Epidemic Preparedness & Response Beijing China; ^3^ Ministry of Education Key Laboratory of Epidemiology of Major Diseases (Peking University) Beijing China; ^4^ State Key Laboratory of Vascular Homeostasis and Remodeling Peking University Beijing China; ^5^ Department of Family Medicine and Primary Care Li Ka Shing Faculty of Medicine The University of Hong Kong Hong Kong SAR China; ^6^ Centre for Safe Medication Practice and Research Department of Pharmacology and Pharmacy Li Ka Shing Faculty of Medicine The University of Hong Kong Hong Kong SAR China; ^7^ The Institute of Cardiovascular Science and Medicine Li Ka Shing Faculty of Medicine The University of Hong Kong Hong Kong SAR China; ^8^ Advanced Data Analytics for Medical Science Limited Hong Kong SAR China; ^9^ Department of Epidemiology School of Public Health and Tropical Medicine Tulane University New Orleans Louisiana USA; ^10^ Department of Nutrition Harvard T.H. Chan School of Public Health Harvard University Boston Massachusetts USA; ^11^ Ageing Epidemiology Research Unit School of Public Health Imperial College London London UK; ^12^ Department of Non‐communicable Disease Epidemiology London School of Hygiene & Tropical Medicine London UK

**Keywords:** drug target, metabolomics, prediction model, proteomics, valvular heart disease

## Abstract

The risk profile of valvular heart disease (VHD) and its underlying mechanisms remain poorly understood. This study aimed to develop and validate a multi‐omics‐based risk prediction model, and to elucidate potential biological mechanisms. Using data from the UK Biobank, Cox proportional hazards and machine learning models (XGBoost and LightGBM) were evaluated for predicting VHD and its subtypes (aortic valve stenosis, AVS; aortic valve regurgitation, AVR; mitral valve regurgitation, MVR). Cox models based on key clinical factors showed the best predictive performance (C‐index of 0.75–0.81), which was further enhanced by incorporating proteomic data (all C‐index > 0.81) but not by genomic or metabolomic data. Notably, a simplified 10‐year model comprising only four top proteins maintained favorable performance (C‐index of 0.75–0.82). Cluster analysis identified blood pressure and lipid levels as leading modifiable risk factors for VHD onset. Functional enrichment analysis revealed that VHD is primarily associated with protease inhibition, AVS with fibrotic and matrix metabolic pathways, and MVR with immune‐inflammatory activation. Mendelian randomization and Bayesian colocalization analyses suggested causal associations between CNTN5 and CD8A with risks of AVS and MVR, whilst IGFBP7 showed a reverse‐direction association with AVS. These findings highlight promising avenues for early diagnostic biomarkers and potential precision‐targeted therapies.

## Graphical abstract

Large‐scale UK Biobank analyses identify clinical and proteomic signatures for early prediction of valvular heart disease and its subtypes. Proteins add predictive value for VHD, AVS, and MVR, with outcome‐specific compact panels showing translational potential. Multi‐layer evidence highlights matrix remodeling, protease regulation, immune inflammation, and genetically supported protein candidates for precision prevention.

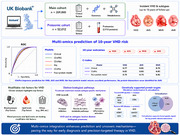

**Abbreviations**
AVS: Aortic Valve Stenosis
BMI: Body Mass Index
CE in S‐HDL: Cholesteryl Esters in Small HDL
CE/TL in IDL%: Cholesteryl Esters to Total Lipids in IDL percentage
DBP: Diastolic Blood Pressure
HbA1c: Glycated Hemoglobin A1c
LA/TFA%: Linoleic Acid to Total Fatty Acids percentage
MVR: Mitral Valve Regurgitation
SBP: Systolic Blood Pressure
S‐HDL‐P: Concentration of Small HDL Particles
TEChol: Total Esterified Cholesterol
TG/TL in IDL%: Triglycerides to Total Lipids in IDL percentage
VHD: Valvular Heart Disease

## 1 Introduction

Valvular heart disease (VHD), predominantly involving degenerative stenosis or regurgitation of the aortic and mitral valves, has emerged as one of the most prevalent forms of cardiovascular diseases, with its incidence rising rapidly over recent decades [1, 2]. Unlike atherosclerotic cardiovascular disease, no effective pharmacological therapy currently exists to halt or reverse the progression of primary VHD [3, 4]. Without timely intervention, the prognosis of VHD is poor; for example, the 5‐year mortality for individuals with moderate or severe aortic stenosis reaches approximately 56% and 67%, respectively [5]. Current evidence indicates that early referral and appropriate surgical treatment offer the greatest potential to restore survival rates to levels comparable to the general population, underscoring the importance of early diagnosis and prompt intervention for at‐risk individuals [6, 7]. Nevertheless, major challenges persist in the risk stratification of VHD and optimal strategies for early prediction and prevention remain to be fully defined. A potential breakthrough has recently emerged with the advancement of big data, driven algorithms and innovative omics technologies [8].

Multi‐omics approaches have shown great potential in elucidating the pathogenesis of VHD and informing therapeutic target discovery [8, 9, 10]. However, it remains unclear whether alterations in omics profiles precede the onset of VHD and its subtypes. Importantly, the predictive power of different omics layers, individually or in combination, across varying time horizons has been largely overlooked. Beyond predictive accuracy, ideal biomarkers for VHD should exhibit high specificity to the underlying pathological process. However, several existing omics‐based and algorithm‐driven models for predicting incident VHD typically achieved a C‐index of 0.75 or below [11, 12, 13]. These findings highlight substantial room for improvement in refining predictive models and uncovering the mechanistic pathways that drive the development of VHD.

In this study, we adopt a data‐driven, multi‐omics approach within a large, well‐characterized prospective cohort with long‐term follow‐up to identify plasma biomarkers of incident VHD and evaluate their predictive performance. Leveraging recent releases from the UK Biobank (UKB), which include comprehensive clinical data, 2910 plasma proteins, 252 circulating metabolites, and summary‐level genome‐wide association study (GWAS) data on over 0.5 million individuals, this study aimed to systematically assess the predictive value and mechanistic links between multi‐omics features and incident VHD as well as its subtypes.

## 2 Results

### 2.1 Study Population

**FIGURE 1:**
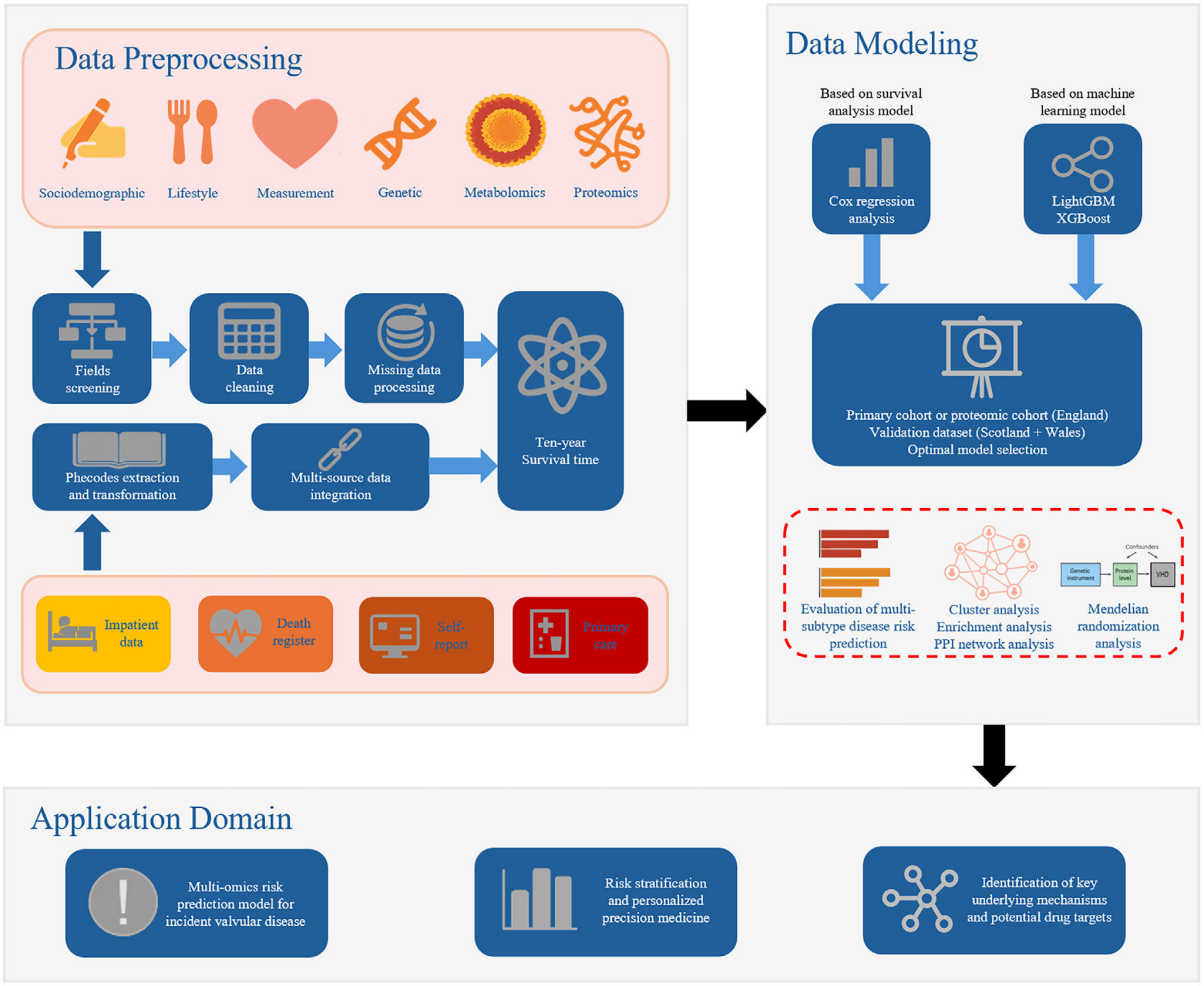
Overview of study design: Diverse data from the UK Biobank are utilized, including six categories: sociodemographic data, lifestyle data, physical measurement data, genetic data, metabolic data, and protein data. Predictor variables are generated after completing field selection, data cleaning, and preprocessing of missing data. Response variables are derived from hospitalization, death, self‐reported, and primary care data, which are standardized to ICD‐10 codes. After temporal alignment of the independent and dependent variables, a 10‐year survival time observation period is constructed, and multiple models are fitted to carry out disease prediction, risk assessment, and bioinformatics analyses. This approach provides ideas for predicting valvular disease risk at baseline, promoting precise risk stratification, and identifying mechanistic pathways.

Figure 1 and Figure S1 illustrate the study design and cohort flowchart. Table 1 presents the baseline characteristics of participants in the primary cohort and the proteomic cohort, both with a mean age of 58 years and 46% male proportion. The two cohorts demonstrated high consistency in sociodemographic and clinical parameters, indicating that the proteomic cohort is highly representative of the overall UK Biobank population. During the 10‐year follow‐up, the primary cohort documented 3800 (1.41%) incident cases of VHD, including 1723 (0.64%) aortic valve stenosis (AVS), 1832 (0.68%) mitral valve regurgitation (MVR), and 546 (0.20%) aortic valve regurgitation (AVR). Similarly, the proteomic cohort recorded 834 (1.60%) incident VHD cases, comprising 367 (0.70%) AVS, 428 (0.83%) MVR, and 105 (0.20%) AVR.

**TABLE 1:** Baseline characteristics of the Primary cohort and the Proteomic Cohort.

	Primary cohort(N = 269840)	Proteomic cohort(N = 52012)
**Age, years, median [IQR]**	58.0 [50.0, 63.0]	58.0 [50.0, 64.0]
**Male sex, n (%)**	124088 (45.97)	23945 (46.02)
**White ethnicity, n (%)**	258028 (95.60)	49071 (94.30)
**Townsend deprivation index, median [IQR]**	−2.20 [‐3.68, 0.44]	−2.05 [‐3.62, 0.77]
**University education, n (%)**	98067 (36.33)	19274 (37.04)
**Never smoking, n (%)**	108395 (40.16)	20857 (40.08)
**Alcohol intake frequency, n (%)**		
Daily or almost daily	54264 (20.10)	10485 (20.15)
Three or four times a week	62710 (23.23)	11820 (22.56)
Once or twice a week	70491 (26.12)	13493 (25.93)
One to three times a month	30119 (11.16)	5685 (10.92)
Special occasions only	30854 (11.43)	6122 (11.76)
Never	21469 (7.95)	4506 (8.66)
**MET for moderate activity, minutes/week, median [IQR]**	480.00 [120.00, 1200.00]	480.00 [120.00, 1200.00]
**MET for vigorous activity, minutes/week, median [IQR]**	240.00 [0.00, 960.00]	240.00 [0.00, 960.00]
**MET for walking, minutes/week, median [IQR]**	693.00 [297.00, 1386.00]	693.00 [297.00, 1386.00]
**Diet score, n (%)**		
0	9218 (3.42)	1755 (3.37)
1	137495 (50.94)	26357 (50.65)
2	98943 (36.66)	19229 (36.95)
3	24251 (8.98)	4695 (9.02)
**Sleep duration, h / day, median [IQR]**	7.00 [7.00, 8.00]	7.00 [7.00, 8.00]
**Discretionary screen‐time, hours/day, median [IQR]**	3.50 [2.50, 5.00]	4.00 [2.50, 5.00]
**Medication Status, n (%)**		
Lipid lowering medication, n (%)	49045 (18.17)	9959 (19.14)
Antihypertensive medication, n (%)	61198 (22.67)	12515 (24.05)
Antidiabetic medication, n (%)	10244 (3.80)	2147 (4.12)
Antiplatelet medication, n (%)	40029 (14.83)	8083 (15.53)
Anticoagulant medication, n (%)	2328 (0.86)	654 (1.26)
Antiresorptive medication, n (%)	4257 (1.58)	968 (1.86)
Vitamin D or Vitamin K supplement, n (%)	7247 (2.68)	1437 (2.76)
Calcium supplement, n (%)	62049 (22.99)	12207 (23.46)
**Physical Measures and Biomarkers**		
Systolic blood pressure, mmHg, mean (SD)	138.00 (19.22)	138.00 (19.28)
Diastolic blood pressure, mmHg, mean (SD)	82.00 (10.45)	82.00 (10.50)
Body mass index, kg/m2, mean (SD)	26.78 (4.78)	26.78 (4.80)
C reactive protein, mg/L, median [IQR]	1.33 [0.66, 2.77]	1.34 [0.66, 2.82]
eGFR, mL/min/1.73m2, median [IQR]	92.77 [82.79, 100.04]	92.52 [82.21, 99.84]
Creatinine, µmol/L, median [IQR]	70.40 [61.40, 80.90]	70.70 [61.40, 81.30]
LDL direct, mmol/L, mean (SD)	3.51 (0.85)	3.49 (0.86)
Lipoprotein A, nmol/L, median [IQR]	21.00 [9.56, 61.70]	21.63 [9.61, 63.32]
Apolipoprotein A, g/L, median [IQR]	1.51 [1.35, 1.70]	1.51 [1.34, 1.70]
Apolipoprotein B, g/L, median [IQR]	1.02 [0.86, 1.18]	1.01 [0.85, 1.18]
Total bilirubin, µmol/L, median [IQR]	8.03 [6.40, 10.37]	8.04 [6.40, 10.39]
Direct bilirubin, µmol/L, median [IQR]	1.51 [1.20, 1.98]	1.51 [1.20, 1.99]
HDL, mmol/L, mean (SD)	1.39 (0.37)	1.39 (0.37)
Triglycerides, mmol/L, median [IQR]	1.49 [1.05, 2.15]	1.48 [1.05, 2.14]
Glycated haemoglobin (HbA1c), mmol/mol, mean (SD)	35.20 (5.76)	35.30 (5.93)
Urate, µmol/L, median [IQR]	303.10 [250.60, 360.80]	303.60 [251.40, 362.40]
Urea, mmol/L, median [IQR]	5.28 [4.50, 6.15]	5.28 [4.49, 6.18]
Serum calcium, mmol/L, mean (SD)	2.38 (0.09)	2.38 (0.09)
Serum phosphate, mmol/L, mean (SD)	1.16 (0.16)	1.16 (0.16)

### 2.2 Prediction of the 10‐Year risk of VHD From Multi‐Modal Clinical Data

**FIGURE 2:**
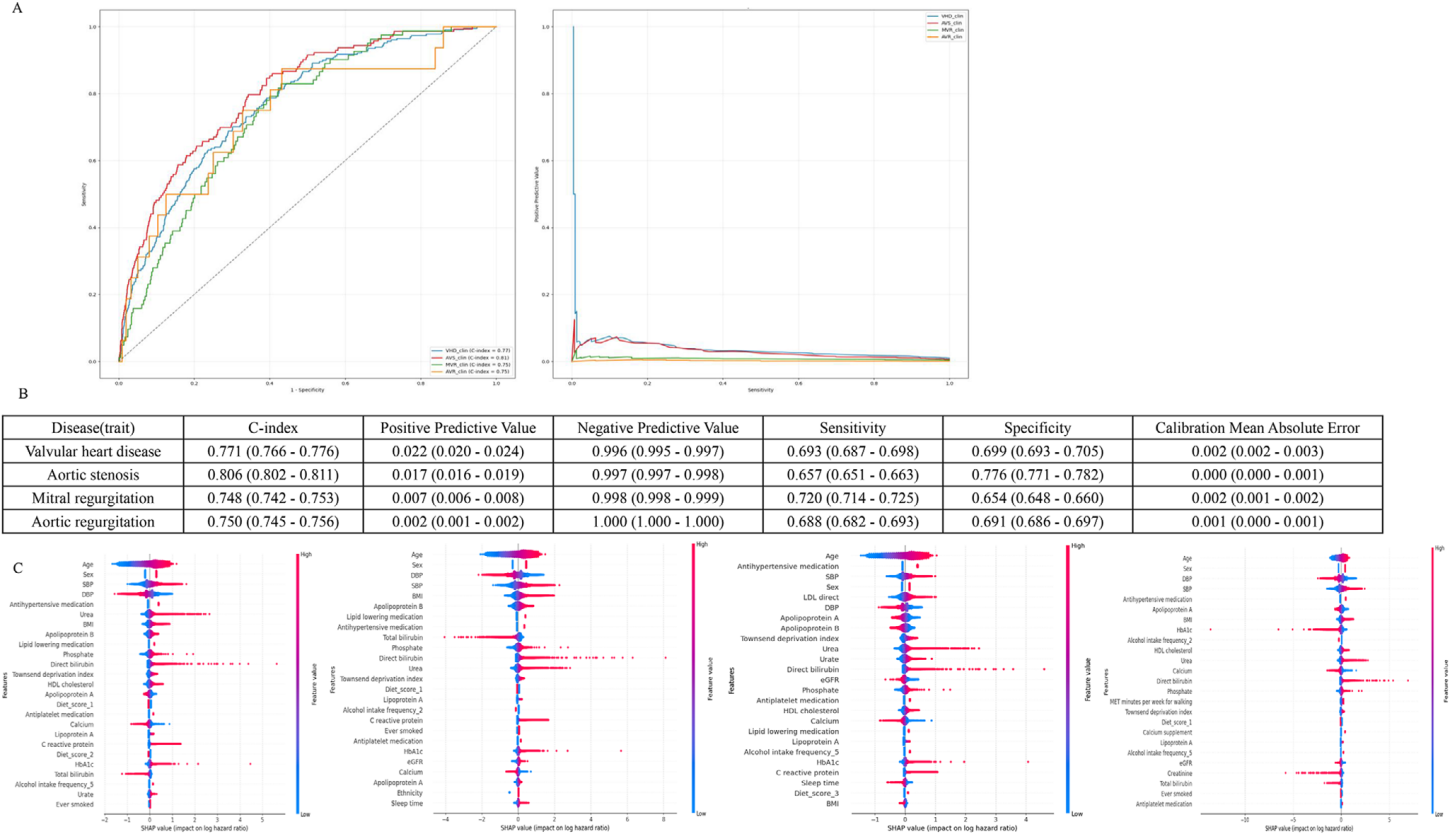
Clinical prediction model of valvular disease risk in the primary cohort (Clin model). (A) ROC curves and precision‐recall curves of valvular disease prediction models. (B) Performance metrics of the Clin model (using the top 25 important features) in the independent test dataset. (C) SHAP visualization plot of the top 25 important features selected. The width of the horizontal bars represents the degree of contribution to disease prediction—the wider the range, the greater the contribution. The color of the horizontal bars indicates the magnitude of the feature values, represented by a gradient from blue (low) to red (high), with the color bar on the right showing the specific color coding. The direction of the x‐axis indicates the likelihood of developing the disease (right side) or remaining healthy (left side). DBP Diastolic Blood Pressure, SBP Systolic Blood Pressure, BMI Body Mass Index, HbA1c Glycated Hemoglobin A1c.

We developed a clinical model (Clin model) based on sociodemographic characteristics and clinical data for predicting the 10‐year risk of VHD diagnosis. In the geographically defined test set comprising participants from Scotland and Wales, the Clin model demonstrated strong predictive performance (C‐index), with values of 0.771 (95% CI: 0.766–0.776) for VHD, 0.806 (95% CI: 0.802–0.811) for AVS, 0.748 (95% CI: 0.742–0.753) for MVR, and 0.750 (95% CI: 0.745–0.756) for AVR (Figure 2A,B). Calibration analysis revealed that the mean absolute error between the predicted and actual risks of the Clin model remained within the range of 0.000–0.002, indicating excellent calibration and strong reliability (Figure 2B). SHAP plots of the top 25 features revealed that age, sex, blood pressure, and body mass index (BMI) were the most important predictors (Figure 2C).

### 2.3 Multi‐Omics VHD Risk Prediction Models

**FIGURE 3:**
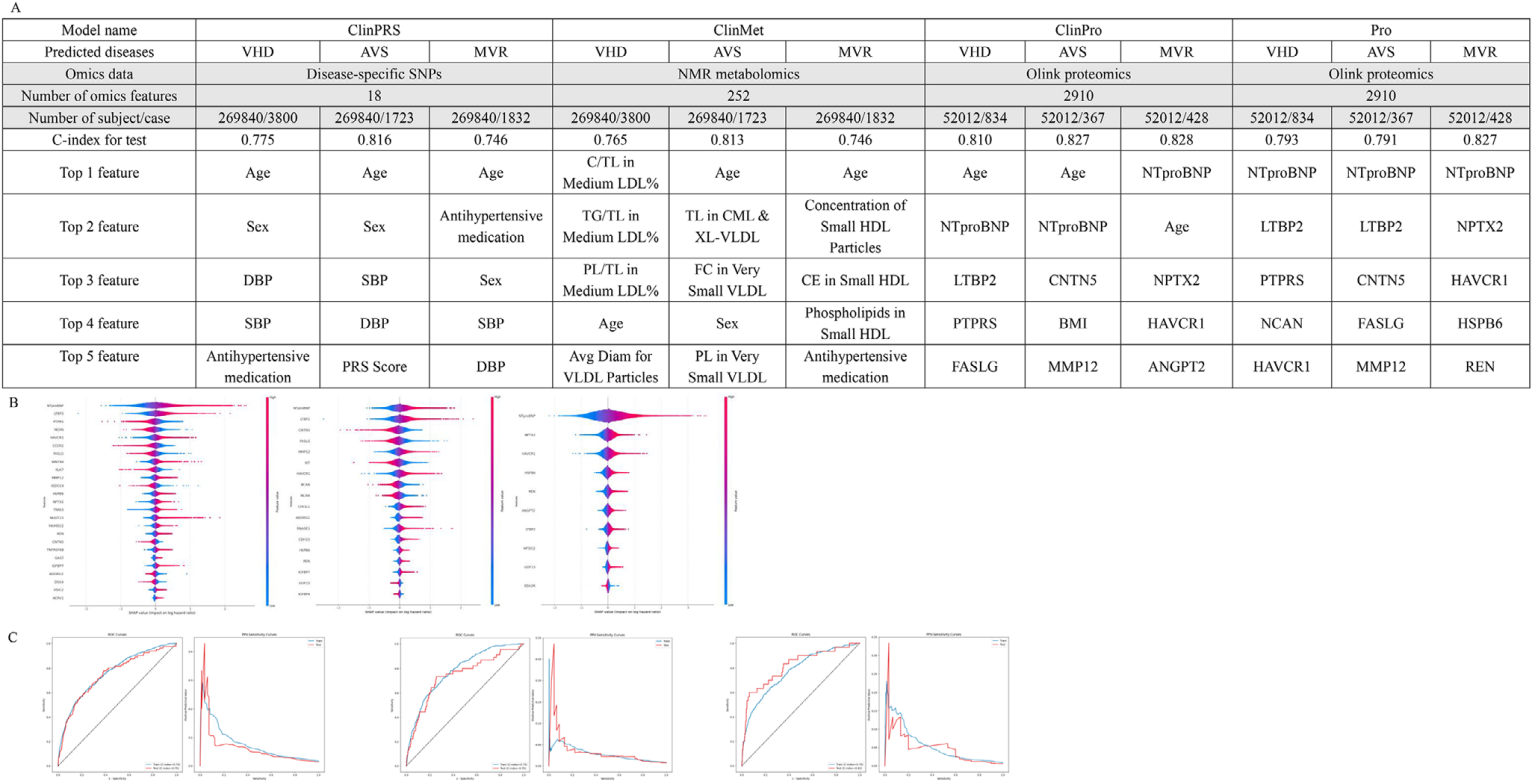
Multi‐omics valvular disease risk biomarkers. (A) Top ranked features from omics models. The C‐index values reported for the Pro model correspond to the full protein‐only LASSO model including all selected protein predictors. (B) SHAP visualization plot of the top 25 important features selected based on the Pro model, covering VHD, AVS, and MVR. (C) The ROC curves and precision‐recall curves of the valvular disease prediction models constructed using only the four most important proteins identified from the Pro model. TG/TL in IDL% Triglycerides to Total Lipids in IDL percentage. CE/TL in IDL% Cholesteryl Esters to Total Lipids in IDL percentage. C/TL in Medium LDL% Cholesterol to Total Lipids in Medium LDL percentage. TG/TL in Medium LDL% Triglycerides to Total Lipids in Medium LDL percentage. PL/TL in Medium LDL% Phospholipids to Total Lipids in Medium LDL percentage. Avg Diam for VLDL Particles Average Diameter for VLDL Particles. TL in CML & XL‐VLDL Total Lipids in Chylomicrons and Extremely Large VLDL. FC in Very Small VLDL Free Cholesterol in Very Small VLDL. PL in Very Small VLDL Phospholipids in Very Small VLDL. TG in CML & XL‐VLDL Triglycerides in Chylomicrons and Extremely Large VLDL. CE in Small HDL Cholesteryl Esters in Small HDL.

Compared to the Clin model, the ClinPRS model showed slightly improved predictive performance only for AVS (C‐index = 0.816) and VHD (C‐index = 0.775). In the validation analysis using the updated AVS PRS, predictive performance was further modestly improved for AVS (C‐index = 0.823) and VHD (C‐index = 0.778), with little to no gain observed for MVR and AVR. Meanwhile, the ClinMet model enhanced the predictive capability for AVS (C‐index = 0.813) but did not improve the prediction of VHD, MVR, and AVR (Figure 3A).

Our study found that the ClinPro model significantly improved performance for VHD (C‐index = 0.810), AVS (C‐index = 0.827), and MVR (C‐index = 0.828), with a particularly notable increase of 0.10 for MVR, highlighting the supplemental predictive value of proteins such as NT‐proBNP and NPTX2. For AVR, no predictive protein biomarkers were identified in this study. Interestingly, based on the feature importance rank of the proteomics model, the use of only the top four proteins effectively predicted valvular heart disease (VHD, C‐index = 0.75), aortic valve stenosis (AVS, C‐index = 0.75), and mitral valve regurgitation (MVR, C‐index = 0.82) in the geographical validation test set, demonstrating favorable predictive 10‐year performance (Figure 3B,C). Compared to the ClinPro model which required 25 features, the model relying solely on these four proteins maintained strong predictive capability while exhibiting greater application potential and clinical translation prospects (Table S1). More detailed Cox regression results and SHAP plots under different models are presented in Table S2 and Figures S2 and S3.

The predictive performance for VHD and subtypes remained largely consistent after setting 5‐ and 15‐years observation periods in the geographical validation test set compared to the 10‐year survival period (Figure S4). For AVS, the performance was poorer at the 5‐year follow‐up (C‐index = 0.63) but consistent at the 15‐year follow‐up (C‐index = 0.73). In contrast, MVR exhibited the opposite pattern, showing higher predictive performance at the 5‐year follow‐up (C‐index = 0.83) but a decline at the 15‐year follow‐up (C‐index = 0.78).

Overall, the models showed acceptable calibration, with calibration slopes generally close to 1.0. However, O/E ratios indicated some absolute risk overestimation, particularly for MVR and AVR, while AVS showed better agreement between observed and expected events (Figure S5 and Table S2). Decision curve analysis showed that most models provided positive net benefit within the 0%–2% threshold probability range. In the primary cohort, net benefit was greater for VHD and AVS across non‐proteomic models. In the proteomic cohort, the Pro and ClinPro models showed favorable net benefit for VHD, AVS, and MVR, supporting the clinical utility of proteomic features (Figure S6). Compared with the PREVENT model, reclassification improvement in the primary cohort was modest and mainly observed for VHD and AVS. In contrast, proteomics‐based models showed stronger reclassification performance, with the ClinPro model achieving categorical net reclassification improvements (NRIs) of 0.095, 0.146, and 0.218 and continuous NRIs of 0.144, 0.105, and 0.222 for VHD, AVS, and MVR, respectively. These results highlight the added value of proteomic features beyond PREVENT, particularly for VHD, AVS, and MVR (Table S3). Sensitivity analyses using lower thresholds of 1.0% and 2.0% for both categorical NRI and continuous NRI yielded findings largely consistent with the primary analysis across VHD, AVS, and MVR, supporting the robustness of the observed reclassification improvements in low‐incidence settings (Tables S4 and S5).

### 2.4 Precision Phenotyping for At‐Risk Population of VHD

**FIGURE 4:**
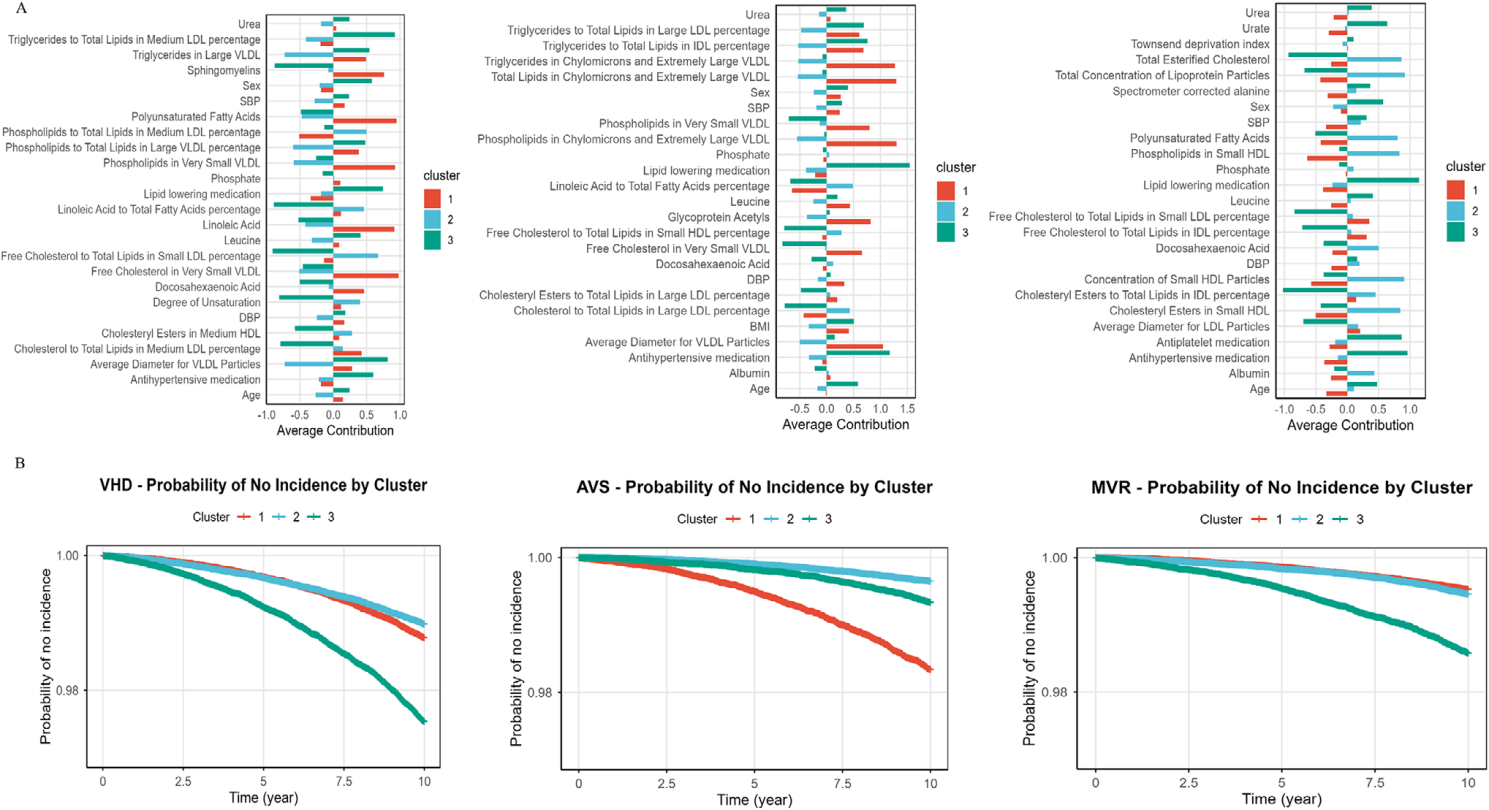
Clustering risk overview in the primary cohort. (A) The feature contribution plots corresponding to the three clustering subtypes, covering VHD, AVS, and MVR. (B) Comparison of the event‐free probability over time across the three clusters generated for each condition: VHD, AVS, and MVR.

After identifying the top 25 most important features in the ClinMet model (Table S6), we detected three clusters within our study population for VHD, AVS, and MVR. By analyzing the feature contributions and prognosis of each cluster, we found that the cluster 3 population was more prone to developing VHD, AVS, and MVR, highlighting the urgency of early intervention for high‐risk groups across the general population (Figure 4A,B and Figure S7). In cluster 3, the key predictors for the progression of VHD were primarily advanced age, higher triglycerides to total lipids in medium LDL percentage, elevated average diameter for VLDL particles, and more frequent use of lipid lowering medication. The high‐risk subgroup for AVS was featured by older age, elevated SBP, higher BMI, and increased urea, along with more use of antihypertensive drugs and lipid lowering medication. Meanwhile, the MVR high‐risk subgroup was featured by older age, increased urea and urate levels, as well as lower concentrations of total esterified cholesterol, reduced polyunsaturated fatty acids, and a decreased percentage of cholesteryl esters to total lipids in IDL (Figure 4A,B and Table S7).

### 2.5 GO and KEGG Enrichment Analysis

**FIGURE 5:**
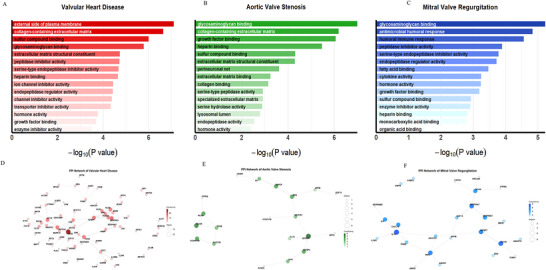
GO Enrichment analysis and protein—protein interaction network of valvular diseases after LASSO‐based screening. (A–C) The GO enrichment analysis for VHD, AVS and MVR. (D–E) The protein—protein interaction network for VHD, AVS and MVR. For the proteins screened in the valve disease model, a protein‐protein interaction (PPI) network was constructed using the STRING database or the average absolute SHAP value. Based on the existing biological information, the score‐threshold was set to 400 when exploring protein‐protein interactions using the STRING database. Additionally, in the PPI network constructed based on the average absolute SHAP values, the size and color of the proteins are determined by their number of connections. Proteins with more connections to other proteins are displayed larger and in a more intense color.

Based on proteomics data, we performed enrichment analysis to explore the biological functions associated with VHD, AVS, and MVR (Table S8). The VHD subtype was primarily characterized by terms related to extracellular matrix structure and protease inhibition, reflecting its role in matrix organization and proteolytic regulation. The AVS subtype also showed enrichment in extracellular matrix components, but was distinguished by unique associations with growth factor binding and perineuronal net structures, suggesting a specialized role in neuro‐environmental regulation. In contrast, the MVR subtype displayed a clear emphasis on humoral immune responses and fatty acid binding, indicating a link between lipid metabolism and antimicrobial defense mechanisms in its pathogenesis (Figure 5A–C). Based on the Kyoto Encyclopedia of Genes and Genomes (KEGG) pathway enrichment analysis, VHD and AVS exhibit distinct signaling pathway profiles. VHD is primarily enriched in the cytokine‐cytokine receptor interaction pathway (hsa04060) and the MAPK signaling pathway (hsa04010), along with significant association with the breast cancer pathway (hsa05224). This pattern suggests that its pathogenesis involves immune‐inflammatory responses, regulation of cell proliferation, and activation of tumor‐related signaling networks. In contrast, AVS shows significant enrichment in the PI3K‐Akt signaling pathway (hsa04151) and the renin‐angiotensin system (hsa04614), indicating a different mechanistic basis potentially related to metabolic regulation and vascular homeostasis (Table S9).

To further characterize shared and subtype‐specific mechanisms, Gene Ontology (GO) and KEGG enrichment analyses were performed for overlapping and phenotype‐specific protein predictors. Shared AVS‐MVR proteins were mainly enriched in growth regulation, blood pressure regulation, extracellular matrix organization, and glycosaminoglycan or growth factor binding. AVS‐specific proteins were enriched in vascular endothelial growth factor signaling and collagen‐containing extracellular matrix, suggesting roles in angiogenic signaling and matrix remodeling. In contrast, MVR‐specific proteins were enriched in peptidase inhibitor activity, fatty acid binding, and cytokine‐related functions, indicating potential involvement of protease regulation, lipid metabolism, and immune‐inflammatory responses (Table S10). No significant KEGG pathways were identified after multiple‐testing correction.

### 2.6 Protein Interaction Networks of VHD

The protein‐protein interaction (PPI) network constructed based on the co‐expression information from the STRING database revealed a highly interconnected protein subnetwork for VHD, AVS, and MVR (Figure 5D–F). Among these proteins, the most densely connected ones include TP53 (a critical tumor suppressor that regulates cell cycle arrest, apoptosis, and DNA repair, with its dysfunction implicated in numerous cancers and other diseases), IGFBP7 (an important metabolic regulator and biomarker of cellular senescence secretome and heart failure), BCAN (a brain‐specific extracellular matrix chondroitin sulfate proteoglycan involved in central nervous system development and synaptic plasticity), IL18 (a pro‐inflammatory cytokine belonging to the IL‐1 family that plays important roles in both innate and adaptive immunity), and REN (an aspartic protease that promoted the generation of angiotensin, thereby regulating the renin‐angiotensin system). For AVS and MVR, LASSO‐identified proteins were ranked based on their mean absolute SHAP values to characterize their biological functions and potential drug targets (Table S1), with emphasis placed on the overlap between the two disease subtypes. We found that two proteins (Growth differentiation factor 15 and Renin) could be identified as druggable targets in the TTD (Table 2).

**TABLE 2:** All proteins identified for AVS and MVR outcomes.

Protein	Biological function	Relevant drug
N‐terminal proBNP	A protein secreted by the ventricles in response to volume overload leading to ventricular dilation, playing a crucial role in the diagnosis and prognosis of cardiovascular diseases [14]	
Latent transforming growth factor beta binding protein 2	A secreted extracellular matrix protein associated with pulmonary fibrosis [15] and dementia pathogenesis [16]	
Hepatitis A virus cellular receptor 1	A transmembrane protein involved in immune regulation and inflammatory responses, often upregulated in tissue injury, reflecting inflammatory activity and cellular stress in cardiovascular conditions [17, 18, 19]	
Heat shock protein beta 6	A small heat shock protein that regulates smooth muscle relaxation and protects cardiomyocytes against stress‐induced damage, contributing to cardiac function and adaptation to hemodynamic stress [20, 21]	
Renin	An enzyme that initiates the renin‐angiotensin system by catalyzing the formation of angiotensin I, playing a central role in blood pressure regulation, fluid balance, and cardiovascular remodeling [22, 23]	Aliskiren
Growth differentiation factor 15	A stress‐responsive cytokine of the transforming growth factor‐β superfamily, released under conditions of inflammation, oxidative stress, and tissue injury, and associated with cardiovascular risk and disease progression [24, 25, 26]	Ponsegromab

In the proteomic cohort, after adjusting for age, sex, BMI, and the top 20 genetic principal components, shared proteins identified by LASSO for AVS and MVR were analyzed for their biological functions and potential drug targets. Druggable target search was performed using the Therapeutic Target Database, at http://db.idrblab.net/.

### 2.7 Mendelian Randomization and Bayesian Colocalization Analysis

**FIGURE 6:**
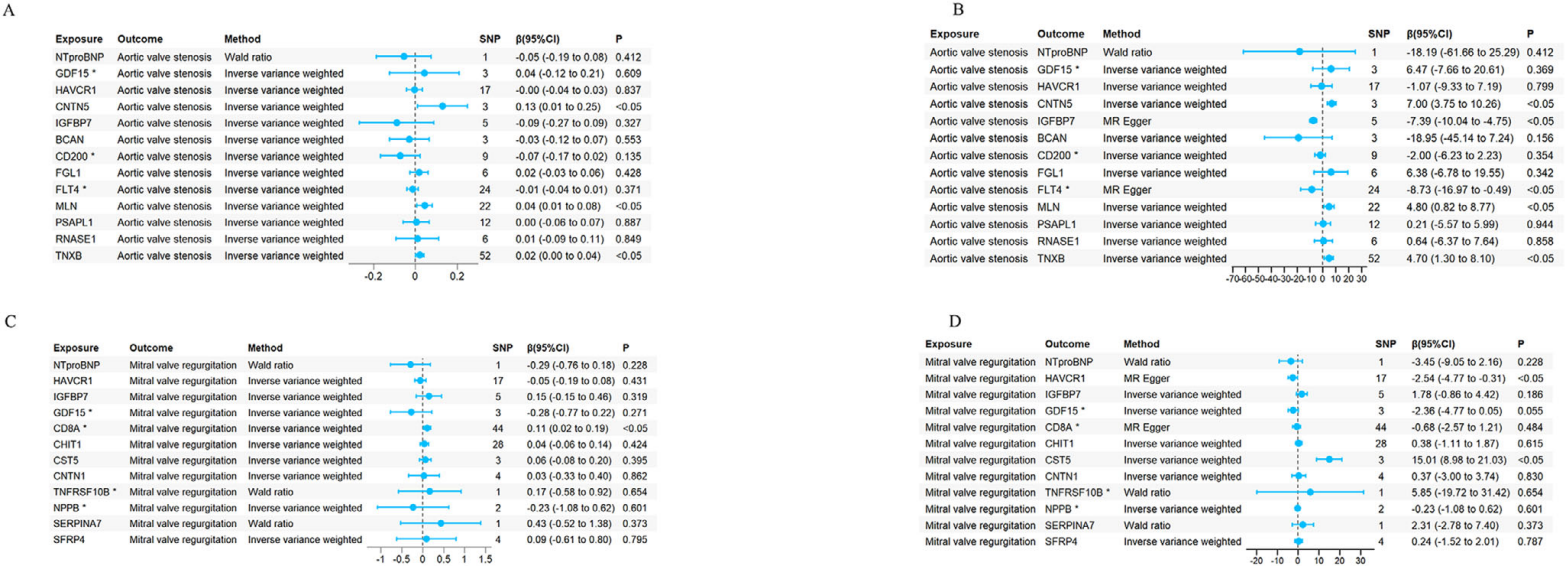
Mendelian randomization analyses between proteomics and valvular disease after LASSO screening. After identifying suitable protein GWAS data in the GWAS catalog (https://www.ebi.ac.uk/gwas/home) and IEU databases (https://gwas.mrcieu.ac.uk), all proteins for AVS and MVR were selected based on their covariate‐adjusted importance rank for the subsequent Mendelian randomization analysis. In cases where evidence of horizontal pleiotropy was present, the MR‐Egger results were used. The instrumental variable threshold for all proteins was set to p < 5e‐08. (A) Forward Mendelian randomization results with protein as the exposure and AVS as the outcome. (B) Backward Mendelian randomization results with AVS as the exposure and proteins as the outcome. (C) Forward Mendelian randomization results with proteins as the exposure and MVR as the outcome. (D) Backward Mendelian randomization results with MVR as the exposure and proteins as the outcome. Proteins with (^*^) are potentially druggable targets (Therapeutic Target Database, at http://db.idrblab.net/).

Detailed results of all instrumental variables and Mendelian randomization (MR) analyses are provided in Tables S11 and S12. The two‐sample MR analyses identified several protein‐disease pairs with evidence supporting different levels and directions of association. Evidence consistent with a potential protein‐to‐disease relationship was observed for CNTN5 (Contactin 5), MLN (Promotilin), and TNXB (Tenascin‐X) with AVS, as well as for CD8A (T‐cell surface glycoprotein CD8 alpha chain) with MVR (P < 0.05; Figure 6A–D). In contrast, reverse‐direction MR analyses suggested that genetic liability to AVS may influence circulating levels of IGFBP7 (Insulin‐like growth factor binding protein 7) and FLT4 (Vascular endothelial growth factor receptor 3), while genetic liability to MVR may influence circulating levels of HAVCR1 (Hepatitis A virus cellular receptor 1) and CST5 (Cystatin‐D) (P < 0.05; Figure 6A–D). Additionally, a borderline reverse MR signal was observed between MVR and GDF15 (Growth differentiation factor 15) (P = 0.055), suggesting that MVR progression may contribute to elevated circulating GDF15 levels (Figure 6D). These reverse‐direction associations should be interpreted cautiously and do not provide evidence for a causal effect of the corresponding proteins on disease risk. Colocalization analysis of the protein‐disease pairs showing significant MR associations identified shared causal variants for three pairs (PP.H4 ≥ 70%; Table S13): CNTN5 with AVS (PP.H4 = 87.56%), CD8A with MVR (PP.H4 = 82.26%), and IGFBP7 with AVS (PP.H4 = 73.03%). Notably, the colocalization signal for IGFBP7 was observed in the absence of significant forward MR evidence and therefore should not be interpreted as evidence of a protein‐to‐disease causal relationship. According to the Therapeutic Target Database, CD200, FLT4, CD8A, NPPB, and TNFRSF10B were classified as druggable targets. For protein‐disease pairs showing significant MR evidence, we additionally generated scatter plots and funnel plots (Figures S8 and S9).

## 3 Discussion

Our clinical model effectively predicted the incidence of VHD and its subtypes, with age, sex, blood pressure, and BMI emerging as the strongest predictors. While adding genetic or metabolomics data yielded a modest improvement in model performance, the incorporation of proteomic data resulted in a substantial enhancement in the prediction of VHD, AVS, and MVR. Notably, comparable predictive accuracy was achieved using only the top four proteins, with NTproBNP serving as the strongest predictor. Extracellular matrix organization and protease inhibition emerged as common pathways, whereas AVS exhibited pronounced enrichment in matrix structural and growth‐factor‐binding functions, and MVR showed stronger immune and inflammatory activity. Several key hub proteins, identified through Mendelian randomization and Bayesian colocalization analyses, were implicated as potentially contributing to the risk of incident AVS or MVR.

Accurate prediction of future disease risk enables both early intervention and the targeted implementation of preventive strategies among high‐risk populations. However, the development of predictive models for VHD or its subtypes remains limited. Although an artificial intelligence‐enhanced electrocardiogram model for predicting valvular regurgitation has been developed, its training and validation on a retrospective, hospital‐based dataset over four years limited its robustness and generalizability [11]. Two additional models with clinical and proteomic predictors focused exclusively on AVS [12, 13]. In contrast, our study is the first to employ a prospective design, systematically evaluate multiple VHD subtypes, integrate advanced machine learning algorithms, and leverage multi‐omics data to enhance predictive performance. Notably, our clinical‐factor‐only model for AVS (C‐index: 0.81) outperformed the aforementioned models that combined clinical and proteomic features, offering a practical and generalizable approach for VHD risk stratification.

While the addition of genomic or metabolomic features offered limited incremental value beyond clinical factors, the integration of proteomic data led to a marked improvement in model performance. This superiority may be attributed to the ability of circulating proteins to capture an individual's current physiological state [27, 28] and serve as early indicators of disease processes, potentially detecting subclinical pathological changes before overt symptom onset. In contrast, PRSs are inherently static, unable to reflect the dynamic stages of disease progression or the influence of environmental and lifestyle factors. Metabolomic profiles, while reflecting short‐term metabolic fluctuations, may provide limited incremental value, partly because the Nightingale NMR platform predominantly captures lipid‐related measures that overlap with conventional clinical variables [29], thereby restricting complementary information, and may also correlate less directly with key disease mechanisms such as valvular remodeling and inflammatory processes compared with proteomic markers. To enhance predictive performance, proteins were selected based on feature importance rather than MR‐derived causal evidence. Importantly, while the top four protein‐only models effectively predicted VHD cases, underscoring the potential of parsimonious proteomic panels as feasible tools for population‐level screening and individualized prevention, it should be noted that the selected proteins were outcome‐specific, with distinct panels identified for VHD, AVS, and MVR. This heterogeneity warrants consideration in clinical translation.

Although the mechanisms of VHD, such as lipid metabolism disorders and mechanical stress, have been systematically investigated [30], both the European Society of Cardiology and American Heart Association guidelines, as well as recent Lancet Series, have primarily focused on the clinical management of established disease rather than on its early prevention [4, 8, 31, 32]. This gap may stem from the limited population‐based evidence available to support the formulation of standardized preventive recommendations for VHD. Degenerative VHD could be prevented by enhancing cardiovascular health status [33, 34], even as we acknowledge ageing as the foremost VHD risk predictor in our clinical model. Our results emphasize that early prevention should focus on comprehensive cardiovascular risk management, particularly through optimizing blood pressure, lipid, and glucose levels, along with promoting healthy lifestyle behaviors.

Extracellular matrix (ECM) organization, protease inhibition, and glycosaminoglycan binding constitute the core biological processes underpinning VHD progression. In particular, collagen‐containing ECM and protease inhibitor activities were consistently enriched across all VHD subtypes, underscoring the central role of fibrotic remodeling in valvular degeneration. For AVS, pathways related to ECM structural constituents and growth factor binding were prominent, aligning with the established calcific‐fibrotic paradigm driven by TGF‐β and BMP signaling [35, 36]. In contrast, MVR demonstrated stronger enrichment in immune and inflammatory responses, suggesting that chronic inflammation and matrix degradation jointly contribute to leaflet dysfunction [37, 38].

At the protein level, IGFBP7 and GDF15 emerged as shared hub molecules across VHD networks. IGFBP7 regulates endothelial‐to‐mesenchymal transition and vascular fibrosis through IGF‐independent mechanisms [39], while GDF15 acts as a stress‐responsive cytokine linked to oxidative stress, inflammation, and maladaptive cardiac remodeling [25]. These proteins have attracted interest as potential therapeutic targets: modulation of IGFBP7 signaling has shown promise in reversing cardiac fibrosis in preclinical studies [25], and elevated GDF15 levels have been proposed as potential both biomarkers and pharmacological targets for valvular and myocardial fibrosis, although the mechanisms mediating these effects remain unclear [40]. Nevertheless, IGFBP7 showed evidence of a reverse‐direction association with AVS in our MR analyses, whereas no consistent evidence was observed for GDF15. Collectively, these findings suggest that IGFBP7 may reflect disease‐related biological processes or represent a downstream biomarker rather than being a causal driver of AVS. At the pathway level, our results suggest that ECM remodeling, protease regulation, and inflammatory‐fibrotic crosstalk may represent relevant biological mechanisms in degenerative VHD, warranting further investigation alongside current surgical and transcatheter approaches.

The strengths of this study include the development of the first multi‐omics‐based prediction model for VHD and its subtypes, supported by a nationwide cohort with long‐term follow‐up. Moreover, we identified key modifiable risk factors for incident VHD and its subtypes, uncovered biological mechanisms underlying disease progression, and revealed multiple potential therapeutic targets for VHD. Several limitations should be acknowledged when interpreting our results. First, outcome ascertainment relied on hospital admission and death registry data, which may miss milder or outpatient‐managed VHD cases and could include undiagnosed prevalent cases in the non‐event group. This may attenuate the observed model performance. Second, external validation in independent, large‐scale prospective cohorts with long‐term follow‐up and available multi‐omics data would strengthen the generalizability of our model. However, such resources remain scarce. To address this, we performed a within‐cohort replication analysis by splitting the UK Biobank sample into training and testing sets based on geographical regions in the UK. Third, although we identified key protein biomarkers with high accuracy for predicting incident VHD and subtypes, the positive predictive value remained low because of the low incidence of VHD, indicating that these biomarkers may be more suitable for risk enrichment or multi‐step screening rather than stand‐alone population screening. Additionally, the use of Olink NPX values on a relative log_2_ scale limits direct clinical applicability. While NPX values are suitable for comparative analyses within a single study, clinical translation requires a separate platform (e.g., ELISA or Olink Flex with calibrators) to measure predictive biomarkers in absolute concentrations (pg/mL). Cross‐platform standardization is also essential, as different proteomics platforms may yield divergent results due to variations in detection limits and sample processing [41]. As such, future validation studies using absolute quantification methods are needed before clinical implementation. Finally, several MR analyses, including those involving IGFBP7, FLT4, and HAVCR1, relied on single genetic instruments and were therefore evaluated using the Wald ratio method. Because horizontal pleiotropy cannot be formally assessed in single‐instrument MR analyses, these findings should be interpreted with caution and regarded as hypothesis‐generating rather than definitive evidence of causality.

This multi‐omics‐enhanced prediction model demonstrated high accuracy in predicting incident VHD and its subtypes. MR and colocalization analyses highlighted potential candidate proteins for future therapeutic investigation and provided insights into disease mechanisms. These findings may lay the groundwork for a risk‐based VHD prediction framework to facilitate earlier detection, timely intervention, and future studies of targeted prevention and therapeutic strategies.

## 4 Experimental Section

### 4.1 Data Source

The UKB is a prospective cohort study that recruited over 0.5 million participants aged 40 to 69 between 2006 and 2010. Participants from England, Scotland, and Wales provided individual‐level data and biological samples at local assessment centers. The UKB cohort has been approved by the NHS National Research Ethics Service North West, and all participants provided written informed consent. This study is based on the UK Biobank application number 592266.

### 4.2 Pre‐Processing of Multifaceted Data

#### 4.2.1 Non‐Omics Data

Variable selection was based on a comprehensive literature review of PubMed, MEDLINE, the Cochrane Library, and Embase for full‐text articles published between January 1, 2000, and December 31, 2024, using search terms such as “aortic valve,” “aortic valve stenosis,” and “aortic valve regurgitation” “mitral valve,” and “mitral regurgitation,” combined with terms related to pathophysiology, epidemiology, natural history, risk factors, and management [33]. After processing participants' sociodemographic characteristics and clinical data at baseline, the selected variables were incorporated into Cox regression and machine learning models. For more details on the selection of these variables, please refer to the supplementary methods section in Table S14.

The diet score assessed participants' regular dietary intake, including the consumption of fruits, vegetables, fish, red meat, and processed meat (Table S15) [42, 43]. The score ranges from 0 to 3, with a higher score indicating a healthier dietary pattern. Discretionary screen time was defined as the total number of hours per day spent on watching TV and non‐occupational computer use. Drug information and supplement information were divided into eight categories (Table S16) [33].

#### 4.2.2 Multi‐Omics Data

Following a literature review, 18 genome‐wide significant loci (P < 5×10^−8^) for AVS were identified (Table S17). As a validation analysis, we additionally identified 257 European‐ancestry SNPs from a recently published polygenic risk score (PRS) for AS (Table S18). Using the reported effect alleles and their corresponding beta values, we computed PRS for each individual by processing the imputed genetic data from the UK Biobank with PLINK (v.2.00a3LM) [44, 45].

Using a high‐throughput proton nuclear magnetic resonance metabolomics platform, 252 circulating metabolite biomarkers in EDTA plasma samples from 274,143 participants in the UK Biobank were quantified [29, 46]. The analysis covered lipid‐related metabolites (cholesterol, triglycerides, fatty acids, etc.), amino acids and related metabolites (alanine amino acids, albumin, etc.), energy metabolism intermediates (glucose metabolism‐related, ketone bodies, etc.), composite indices and ratios (cholesterol‐to‐total lipid ratio, etc.), and other metabolites.

Proteomic analysis was performed on blood samples from 52 999 participants through Olink Analytical Services in Sweden, providing Normalized Protein eXpression values on a log_2_ scale. After data filtering, only biosamples from the baseline survey were included, covering 2922 proteins. The participants included in the proteomic analysis were highly representative of the UKB population. Detailed sample selection, processing, and quality control procedures have been reported in previous studies [47, 48].

#### 4.2.3 Missing Data Imputation and Data Cleaning

Missing values for all non‐omics UKB data were imputed using the R package mice [49]. The covariates included in the imputation models were listed in Tables S19 and S20, including all variables used in the analysis models plus candidate predictors. For metabolites and proteins, we first excluded all variables with a missing rate greater than 20%, and then utilized the miceforest package in Python with five iterations to impute the remaining missing values, resulting in a single dataset [50]. A total of 252 metabolites and 2910 proteins were included in the subsequent analysis.

For outlier treatment, 1%–99% quantile truncation was used for continuous variables for outlier detection and truncation to reduce the interference of extreme values in clinical model prediction. However, original values were retained for the following features: Age, BMI, Townsend deprivation index, sleep time, discretionary screen time, MET minutes per week for moderate activity, MET minutes per week for vigorous activity, and MET minutes per week for walking.

#### 4.2.4 Assessment of Valvular Heart Disease

The study outcome was the occurrence of non‐rheumatic VHD, including AVS, AVR, and MVR. The International Classification of Diseases, 10th Revision (ICD‐10) codes for AVS (I35.0 and I35.2), AVR (I35.1), and MVR (I34.0) were identified from hospital admission records and death registry data [51], with prior validation studies supporting their high diagnostic accuracy (positive predictive value >80%) [33, 52, 53, 54]. Participants with pre‐existing valvular conditions at baseline were excluded, including rheumatic valvular disease, non‐rheumatic valvular disease, congenital valvular abnormalities, endocarditis with VHD, and Marfan syndrome, based on primary care data, hospital inpatient data, death register records, and self‐reported medical conditions (Table S21).

#### 4.2.5 Study Cohorts

Based on the availability of non‐omics data and multi‐omics data, two prospective cohorts were established: the main cohort includes 269 840 participants with sociodemographic characteristics, clinical information, genomic, and metabolomic data; the proteomic cohort comprises 52 012 participants, encompassing sociodemographic characteristics, clinical information, and proteomics data. In addition to meeting the exclusion criteria of the main cohort, participants in the proteomic cohort must also possess the top 20 genetic components for subsequent covariate adjustment. Subsequently, based on the baseline assessment center location, the two cohorts were divided into training and testing sets, with participants from England forming the training set (approximately 90%) and participants from Scotland/Wales forming the testing set (approximately 10%) for geographical validation within the UKB cohort. This geographically defined split may capture regional differences in population characteristics, lifestyle factors, dietary habits, and healthcare‐related factors, thereby providing a more rigorous assessment of model generalizability. Participants were followed from recruitment until the first occurrence of an outcome event, death from other causes, loss to follow‐up, or the study endpoint, whichever occurred first. The primary analysis used a 10‐year follow‐up endpoint. To assess the robustness of the findings, extension analyses were additionally performed using 5‐year and 15‐year endpoints.

#### 4.2.6 Model Selection

In the primary cohort, we compared the performance of three different models (Cox regression, LightGBM, and XGBoost) in predicting valvular heart disease using sociodemographic and clinical data, with the aim of selecting the optimal model for subsequent analysis. For each model, we employed 41 non‐omics features as inputs, applying LASSO regression for feature selection with five‐fold internal cross‐validation on the training set (*n* = 245,584). Hyperparameter optimization was conducted via Optuna (20 trials) to determine the optimal LASSO parameters (alpha_min_ratio and max_iter) as well as the best‐performing configurations for the two machine learning algorithms. The selected non‐zero features from the training set were then applied to a geographical test set (*n* = 24 256) for validation. Ultimately, the Cox regression model achieved superior test‐set performance for VHD, AVS, and MVR, suggesting that it may be better suited for predicting valvular heart disease outcomes in this study. Given its overall consistency, generalizability, and interpretability, the Cox regression model was selected as the final model (Table S22).

The LightGBM model was trained using the Cox partial likelihood loss (objective = ‘cox’) within the LightGBM library (v.4.6.0) and follow‐up time was defined from recruitment until the first occurrence of the outcome of interest, death from other causes, loss to follow‐up, or the study endpoint (10 years for the primary analysis). The predicted log hazard ratios were converted to absolute risk estimates via Cox recalibration. Hyperparameters, including learning rate (1e‐5 to 1e‐2), number of leaves (16–96), maximum tree depth (4–7), and L1/L2 regularization (1e‐3 to 10.0), were optimized using the concordance index (C‐index).

The XGBoost model based on tree structure was implemented using the XGBoost library (v.3.0.0). The key parameters optimized during model training included the learning rate (ranging from 1e‐5 to 1e‐2), maximum tree depth (3–8), minimum child weight (1–5), gamma regularization (1e‐6 to 1.0), subsample ratio (0.6–1.0), and regularization terms (L1/L2, ranging from 1e‐3 to 10.0). The model utilized the Cox proportional hazards objective function for survival analysis and employed early stopping with 100 rounds of patience to prevent overfitting.

In all models, feature selection was performed via LASSO regularization. Mean absolute SHAP values, which quantify the average marginal contribution of each feature across all possible feature combinations, were then computed for the LASSO‐retained features. Up to 25 features were retained in the final models, as model performance stabilized when approximately 25 features were included, supporting a balance between predictive performance and parsimony. The model performance was comprehensively evaluated through multi‐dimensional metrics: (1) Discriminative ability was quantified using the C‐index to assess prediction ranking accuracy; (2) Classification performance was calculated based on optimal cutoff values, including positive predictive values/negative predictive values and sensitivity/specificity; (3) Calibration was evaluated by computing the mean absolute error between predicted risks and actual risks through grouped calibration curves. Calibration plots were generated using both raw and Cox recalibrated predictions, with perfect calibration represented by the 45‐degree line. Calibration accuracy was further assessed using the calibration slope [55, 56, 57]; (4) Clinical utility was evaluated using decision curve analysis [58], with net benefit calculated across threshold probabilities from 0% to 2% and compared against the “intervention for all” and “intervention for none” strategies; (5) Incremental predictive value was assessed by calculating the NRI with the PREVENT model [59] as the reference. The PREVENT model incorporates established cardiovascular risk factors including age, sex, current smoking, diabetes, eGFR, BMI, SBP, total and HDL cholesterol, antihypertensive medications, and lipid‐lowering medications. Both categorical NRI (10‐year risk threshold of 5%) and continuous NRI (5% risk difference threshold) were computed [60]. In addition, sensitivity analyses were performed using 1.0% and 2.0% 10‐year risk thresholds for categorical NRI, as well as 1.0% and 2.0% risk difference thresholds for continuous NRI, for VHD, AVS, and MVR to align with the observed low incidence in the study population. All metrics were calculated with 95% confidence intervals using the bootstrap method (n = 100 repetitions), with all models developed in the training set and evaluated in the test set.

### 4.3 Statistical Analysis

All statistical analyses were performed using Python v.3.10 and R v.4.2.1. This study ultimately employed the Cox proportional hazards regression model to evaluate longitudinal associations between baseline non‐omics/multi‐omics data and valvular heart disease risk. To identify which risk biomarkers are most important for predicting disease incidence, we further quantified the contribution of each feature in the model to individual prediction outcomes using mean absolute SHAP values. We integrated sociodemographic characteristics and clinical data into a Cox proportional hazards regression model to develop a clinical model (Clin model). Additionally, we also integrated multi‐omics data with clinical features for model construction, including genomics (ClinPRS model), metabolomics (ClinMet model), and proteomics (ClinPro model), with the ClinPro model based on the proteomic cohort instead of the main cohort, where a Clin model was also developed for within‐cohort comparison.

After establishing predictive performance in the overall population, we conducted cluster analysis using the top 25 important features selected by LASSO for the final predictive model and their corresponding mean absolute SHAP values in the primary cohort to elucidate heterogeneity in feature importance across subgroups. This approach ensured consistency with the feature set used for prediction model construction. This analysis incorporated non‐omics and metabolomic data, as these data were available in the largest sample size and captured dynamic phenotypic and metabolic states, whereas proteomic data were limited to a smaller subset and genomic data reflected relatively static genetic susceptibility. Positive and negative mean SHAP values were combined to indicate whether each feature acted as a risk or protective factor at the population level. The clustering process involved determining the optimal number of clusters using the elbow method implemented in the R package “cluster,” followed by K‐means clustering to obtain the feature contribution levels of different clusters. Subsequently, downstream predictive performance and survival analyses were conducted to distinguish high‐risk, medium‐risk, and low‐risk populations for valvular heart disease.

In the proteomic cohort, using the optimal parameters determined by the ClinPro model, proteins associated with valvular heart disease were identified for subsequent analysis after adjusting for age, sex, body mass index (BMI), and the top 20 genetic principal components. Functional enrichment analysis [including GO biological processes, GO cellular components, GO molecular functions, and KEGG pathways] and PPI network data were implemented using the R packages clusterProfiler, org.Hs.eg.db, enrichplot, igraph, and STRINGdb [61]. For functional enrichment analysis, we used all non‐zero protein features selected by LASSO as input, with both pvalueCutoff and qvalueCutoff set to 0.05. Given the existence of both significantly enriched parent and child GO terms, the GO term list may become excessively lengthy. To improve interpretability, the simplify function was employed with a threshold of 0.8 to remove redundant GO terms while retaining the most significant ones. GO and KEGG enrichment analyses were further applied to overlapping and phenotype‐specific protein predictors to characterize shared and subtype‐specific biological mechanisms. We then explored the biological functions and drug targets of proteins shared by AVS and MVR.

After obtaining summary data from GWAS in the GWAS Catalog and IEU databases, we performed bidirectional two‐sample MR analysis to evaluate genetically predicted associations between protein exposures and the diseases using the TwoSampleMR package (v.0.6.19) in R, with the inverse‐variance weighted (IVW) method providing the primary causal estimates [62]. Sensitivity analyses were conducted using robust methods including MR‐Egger and weighted median approaches. This analysis systematically evaluated genetically supported associations between proteins with suitable GWAS data and AVS as well as MVR (both with available GWAS data) using GWAS summary statistics from two independent populations. Genetic instruments were selected as SNPs associated with any protein at genome‐wide significance (*P* < 5 × 10^−8^). To avoid confounding by complex linkage disequilibrium (LD) and pleiotropy, SNPs within the major histocompatibility complex region (chr6: 25.5–34.0 Mb) were excluded. All retained SNPs underwent rigorous quality control, including LD clumping using a European reference panel with a 100 kb window and an r^2^ threshold of < 0.001. For each protein‐related instrument, we calculated the F‐statistic and removed those with F < 10 to mitigate weak instrument bias. When only a single instrumental variable remained for a protein, causal effects were estimated using the Wald ratio method.

To investigate whether plasma proteins and disease risk share causal genetic variants and to reduce the possibility that MR associations were driven by linkage disequilibrium, we performed Bayesian colocalization analysis using the “coloc” R package [63]. For each protein, SNPs within ±500 kb of its pQTL locus were included in this analysis. Default parameters were employed: p1  =  1 × 10^−4^ (prior probability that a SNP is associated with the protein only), p2  =  1 × 10^−4^ (prior probability that a SNP is associated with AS only), and p12  =  1 × 10^−5^ (prior probability that a SNP is associated with both traits) [64]. We tested five hypotheses: H0 (no association), H1/H2 (association with one trait only), H3 (distinct causal variants for the two traits), and H4 (a shared causal variant). A posterior probability for H4 greater than 70% was considered strong evidence of colocalization [64, 65].

## Author Contributions

**Zhihao Jiang**: Conceptualization, Software, Formal analysis, Visualization, Writing – original draft, Writing – review & editing. **Yang Liu**: Conceptualization, Software, Validation, Visualization, Writing – original draft, Writing – review & editing. **Mingyu Song, Ning Chen**: Conceptualization, Software, Validation. **Canqing Yu**, **Jun Lv**, **Eric Yuk Fai Wan, Lu Qi** and **Liming Li**: Writing – review & editing. **Bang Zheng** and **Dianjianyi Sun**: Conceptualization, Supervision, Resources, Funding acquisition, Writing – original draft, Writing – review & editing. **Zhihao Jiang** and **Yang Liu**: contributed equally as co‐first authors. **Bang Zheng** and **Dianjianyi Sun**: contributed equally as co‐corresponding authors. The corresponding authors attest that all the listed authors meet authorship criteria and that no others meeting the criteria have been omitted.

## Funding

This work was supported by the National Key R&D Program of China (2023YFC2509400) and the National Natural Science Foundation of China (82061Y0067 and 82103920).

## Conflicts of Interest

EYFW has received research grants from the Health Bureau, the Hong Kong Research Grants Council, Narcotics Division, Security Bureau, Social Welfare Department, Labour and Welfare Bureau of the Government of the Hong Kong SAR and National Natural Science Foundation of China; serves on member of Core Team for Expert Group on Drug Registration of Pharmacy and Poisons Board, and is the director of Advance Data Analytics for Medical Science (ADAMS) Limited (HK). These are outside the submitted work. All other authors disclose no conflicts of interest.

## Supporting information




**Supporting file**: advs76345‐sup‐0001‐SuppMat.docx

## Data Availability

Researchers can apply for access to UK Biobank resources and related data on the study website (https://www.ukbiobank.ac.uk/).
