# Supplementary material for: Harnessing Large‐Scale Multi‐Omics Data for Risk Prediction and Deep Phenotyping of Valvular Heart Diseases in the General Population
Source: Adv Sci (Weinh). 2026 Jul 6;13(53):e76345. doi: 10.1002/advs.76345 (PMC13336115; doi:10.1002/advs.76345)
Supplement: Supplementary file 1 [file ADVS-13-e76345-s001.docx]

Supporting Information

**Harnessing Large-Scale Multi-Omics Data for Risk Prediction and Deep Phenotyping of Valvular Heart Diseases in the General Population**

Zhihao Jiang^*^, Yang Liu^*^, Mingyu Song, Ning Chen, Canqing Yu, Jun Lv, Eric Yuk Fai Wan, Lu Qi, Liming Li, Dianjianyi Sun^#^, Bang Zheng^#^

#Corresponding authors:

Bang Zheng & Dianjianyi Sun,

Department of Epidemiology & Biostatistics, Peking University Health Science Center, 38 Xueyuan Road, Beijing 100191, China; London School of Hygiene & Tropical Medicine, London, UK

Email: zhengbang2@bjmu.edu.cn; dsun1@bjmu.edu.cn

**Supplementary Information Text**

**Methods S1.** Inclusion criteria for cohort.

**Figure S1.** Flow chart of the study.

**Figure S2.** Predictive performance across different feature thresholds.

**Figure S3.** SHAP plot of multi-omics valvular disease risk biomarkers.

**Figure S4.** Predictive performance of the top four proteins for VHD, AVS, and MVR subtypes at 5-year and 15-year survival timepoints.

**Figure S5.** Calibration Analysis of Multi-Omics Prediction Models for Incident Valvular Heart Disease.

**Figure S6.** Decision Curve Analysis of Prediction Models for Incident Valvular Heart Disease.

**Figure S7.** Valvular heart disease clustering analysis status.

**Figure S8.** Significant Mendelian randomization associations involving AVS.

**Figure S9.** Significant Mendelian randomization associations involving MVR.

**Table S1.** Proteins selected by LASSO regression without covariate adjustment.

**Table S2.** Cox regression analysis results.

**Table S3.** Net-reclassification indices for multi-omics-based risk prediction model compared to PREVENT model (hold-out validation cohort).

**Table S4.** Net reclassification indices for multi-omics-based risk prediction models compared with the PREVENT model using a 1% 10-year risk threshold (hold-out validation cohort)

**Table S5.** Net reclassification indices for multi-omics-based risk prediction models compared with the PREVENT model using a 2% 10-year risk threshold (hold-out validation cohort)

**Table S6.** The clustering analysis features screened by LASSO regression.

**Table S7.** Clustering feature contribution for three valvular heart disease subtypes.

**Table S8.** Proteins selected by LASSO regression after covariate adjustment.

**Table S9.** KEGG pathway analysis results.

**Table S10.** GO Enrichment Analysis of Shared and Subtype-Specific Protein Predictors.

**Table S11.** Instrumental variables for plasma proteins.

**Table S12.** Results of Mendelian Randomization analysis.

**Table S13.** Bayesian colocalization for causal plasma proteins and diseases.

**Table S14.** Rationale for selection of risk factors.

**Table S15.** The scoring system of diet.

**Table S16.** Drug classification and data coding.

**Table S17.** SNP loci included in the construction of the polygenic risk score.

**Table S18.** SNP loci included in the validation polygenic risk score analysis.

**Table S19.** Missing percentage and reason for variables in primary cohort.

**Table S20.** Missing percentage and reason for variables in proteomic cohort.

**Table S21.** ICD codes and Field ID defining diseases in UKB.

**Table S22.** Comparative analysis of the clinical performance of three different predictive models.

**Methods S1. Inclusion criteria for cohort.**

1. **Between 2006 and 2010, more than half a million participants in the UK Biobank aged 37-73 years.**

**Exclusion criteria for cohort:**

1. **Prevalent valvular heart disease at baseline.**

(1)	Rheumatic valve disease

Rheumatic mitral valve diseases

·Rheumatic mitral stenosis/ ·Rheumatic mitral insufficiency /·Rheumatic mitral stenosis with insufficiency/

·Other rheumatic mitral valve diseases/ ·Rheumatic mitral valve disease, unspecified

Rheumatic aortic valve diseases

· Rheumatic aortic stenosis/ · Rheumatic aortic insufficiency/ · Rheumatic aortic stenosis with insufficiency/

· Other rheumatic aortic valve diseases/ ·Rheumatic aortic valve disease, unspecified

Rheumatic tricuspid valve diseases

·Rheumatic tricuspid stenosis/ ·Rheumatic tricuspid insufficiency ·Rheumatic tricuspid stenosis with insufficiency/

·Other rheumatic tricuspid valve diseases/ ·Rheumatic tricuspid valve disease, unspecified

(2)	Congenital valve disease or Marfan’s syndrome disease

Congenital malformations of aortic and mitral valves

·Congenital aortic stenosis/ ·Congenital aortic insufficiency/ ·Congenital mitral stenosis/

·Congenital mitral insufficiency/ ·Hypoplastic left heart syndrome/

·Other congenital malformations of aortic and mitral valves/

·Congenital malformation of aortic and mitral valves, unspecified

Congenital malformations of pulmonary and tricuspid valves

·Congenital pulmonary stenosis/ ·Congenital pulmonary insufficiency/ ·Congenital tricuspid stenosis

·Ebstein's anomaly / ·Other congenital malformations of tricuspid valve /

·Congenital malformation of tricuspid valve, unspecified

Marfan's syndrome

(3)	Endocarditis with Valvular Heart Disease

·Endocarditis, valve unspecified/ ·Mitral valve disorders in diseases classified elsewhere/

·Aortic valve disorders in diseases classified elsewhere/ · Pulmonary valve disorders in diseases classified elsewhere

· Multiple valve disorders in diseases classified elsewhere/

· Endocarditis, valve unspecified, in diseases classified elsewhere

(4)	Non-rheumatic valve disease / Degenerative valve disease

Nonrheumatic aortic valve disorders

·Aortic stenosis/ ·Aortic insufficiency/·Aortic stenosis with insufficiency/·Other aortic valve disorders/

·Aortic valve disorder, unspecified

Nonrheumatic mitral valve disorders

·Mitral stenosis/ ·Mitral insufficiency/·Mitral prolapse/·Other nonrheumatic mitral valve disorders/

·Nonrheumatic mitral valve disorder, unspecified/

Nonrheumatic tricuspid valve disorders

·Nonrheumatic tricuspid stenosis/ · Nonrheumatic tricuspid insufficiency

· Nonrheumatic tricuspid stenosis with insufficiency/ ·Other nonrheumatic tricuspid disorders/

·Nonrheumatic tricuspid valve disorder, unspecified

(5)	Pulmonary valve disease

·Pulmonary stenosis/·Pulmonary insufficiency/·Pulmonary stenosis with insufficiency

·Other pulmonary valve disorders/·Pulmonary valve disorder, unspecified

(6)	Multiple valve disease

·Disorders of both mitral and aortic valves/ ·Disorders of both mitral and tricuspid valves/

·Disorders of both aortic and tricuspid valves/ ·Combined disorders of mitral, aortic and tricuspid valves/

·Other multiple valve diseases/ ·Multiple valve disease, unspecified

2. **Lack of genomics and metabolomics data at baseline in the primary cohort.**
3. **Lack of proteomics and top 20 genetic principal components data at baseline in the proteomic cohort.**

**Figure S1. Flow chart of the study.**

**Figure S2. Predictive performance across different feature thresholds.**

A

D

C

B

Predictive Performance Across Different Feature Count Thresholds. ROC curves comparing model discrimination for **(A)** VHD; **(B)** AVS; **(C)** MVR; **(D)** AVR. Within each panel, ROC curves are shown for Clin, ClinPRS, ClinMet, ClinPro, and Pro models (ClinPro and Pro models were excluded for AVR due to the absence of proteomics-based feature selection).

**Figure S3. SHAP plot of multi-omics valvular disease risk biomarkers.**

A

B

**A** SHAP visualization plot of the top 25 important features selected based on ClinMet model, covering VHD, AVS, and MVR. **B** SHAP visualization plot of the top 25 important features selected based on ClinPro model, covering VHD, AVS, and MVR.

**Figure S4. Predictive performance of the top four proteins for VHD, AVS, and MVR subtypes at 5-year and 15-year survival timepoints.**

A

B

C

**A** The ROC curves and precision-recall curves of the VHD prediction model using only the top four most important proteins based on the Pro model, evaluated at 5-year and 15-year survival time points. **B** The ROC curves and precision-recall curves of the AVS prediction model using only the top four most important proteins based on the Pro model, evaluated at 5-year and 15-year survival time points. **C** The ROC curves and precision-recall curves of the MVR prediction model using only the top four most important proteins based on the Pro model, evaluated at 5-year and 15-year survival time points.

**F****igure S5. Calibration Analysis of Multi-Omics Prediction Models for Incident Valvular Heart Disease**

A

B

C

F

E

D

Calibration curves showing the agreement between predicted (both raw and recalibrated) and observed 10-year risks for VHD, AVS, MVR, and AVR across clinical **(A)**, clinical + PRS **(B)**, clinical + metabolomic **(C)**, clinical + metabolomic + PRS **(D)**, proteomic-only **(E)**, and clinical + proteomic **(F)** models. Raw probabilities are shown with solid lines and filled circles and Cox-recalibrated probabilities are shown with dash-dotted lines and filled squares. The diagonal dashed line represents perfect calibration.

**Figure S6.** **Decision Curve Analysis of Prediction Models for Incident Valvular Heart Disease.**

C

B

A

E

F

D

Decision curve analysis comparing the net benefit of clinical **(A)**, clinical + PRS **(B)**, clinical + metabolomic **(C)**, clinical + metabolomic + PRS models **(D)**, proteomic-only **(E)**, and clinical + proteomic **(F)** against treat-all and treat-none strategies across threshold probabilities of 0-2% for predicting 10-year risk of VHD, AVS, MVR, and AVR.

**Figure S7.** **Valvular heart disease clustering analysis status.**

A

B

**A** Construct three clustered feature heat maps based on the top 25 features screened by LASSO, covering VHD, AVS, and MVR. **B** Distribution of risk scores for individual clusters of VHD, AVS, and MVR.

**Figure S8. Significant Mendelian randomization associations involving AVS.**

B

A

D

C

E

F

H

G

Scatter plot of MR, with red representing IVW, blue representing MR-Egger, and green representing Weighted median. **A** CNTN5 → AVS. **B** AVS → CNTN5. **C** AVS → IGFBP7. **D** AVS → FLT4. **E** MLN → AVS. **F** AVS → MLN. **G** TNXB → AVS. **H** AVS → TNXB.

**Figure S9. Significant Mendelian randomization associations involving MVR.**

B

A

C

D

Scatter plot of MR, with red representing IVW, blue representing MR-Egger, and green representing Weighted median. **A** MVR → HAVCR1. **B** MVR → GDF15. **C** CD8A → MVR. **D** MVR → CST5.

**Table S1. Proteins selected by LASSO regression without covariate adjustment.**

| Disease outcome | UniProt ID | Gene name | Protein name | Entrez ID | Mean absolute SHAP value | Mean SHAP Value |
| --- | --- | --- | --- | --- | --- | --- |
| Valvular heart disease | None | NTproBNP | N terminal prohormone of brain natriuretic peptide | None | 0.326112 | 0.041922 |
|  | Q14767 | LTBP2 | Latent transforming growth factor beta binding protein 2 | 4053 | 0.191601 | 0.027487 |
|  | O75255 | PTPRS | Receptor type tyrosine protein phosphatase S | 5802 | 0.134711 | -0.00176 |
|  | O14594 | NCAN | Neurocan | 1463 | 0.129081 | -0.031146 |
|  | F1CME6 | HAVCR1 | Hepatitis A virus cellular receptor 1 | 26762 | 0.116687 | -0.001614 |
|  | I3L3R5 | CCER2 | Coiled coil domain containing glutamate rich protein 2 | 643669 | 0.112063 | 0.003839 |
|  | A0A0H3VB22 | FASLG | Tumor necrosis factor ligand superfamily member 6 | 356 | 0.103761 | -0.015849 |
|  | O14904 | WNT9A | Protein Wnt 9a | 7483 | 0.085152 | -0.014119 |
|  | P49862 | KLK7 | Kallikrein 7 | 5650 | 0.080934 | 0.004223 |
|  | B2R9X8 | MMP12 | Macrophage metalloelastase | 4321 | 0.072069 | -0.004698 |
|  | Q8TDY8 | IGDCC4 | Immunoglobulin superfamily DCC subclass member 4 | 57722 | 0.071292 | -0.013327 |
|  | A0A384MEB1 | HSPB6 | Heat shock protein beta 6 | 126393 | 0.070552 | -0.0037 |
|  | P47972 | NPTX2 | Neuronal pentraxin 2 | 4885 | 0.068122 | -0.000299 |
|  | B6D427 | TNNI3 | Troponin I cardiac muscle | 7137 | 0.067618 | -0.002012 |
|  | Q9NV35 | NUDT15 | Nudix hydrolase 15 | 55270 | 0.065532 | -0.003244 |
|  | Q7Z304 | MAMDC2 | MAM domain containing 2 | 256691 | 0.055427 | 0.014159 |
|  | A0A1B0GUZ2 | REN | Renin | 5972 | 0.052951 | 0.006662 |
|  | A1L4P0 | CNTN5 | Contactin 5 | 53942 | 0.046874 | -0.012041 |
|  | O95407 | TNFRSF6B | Tumor necrosis factor receptor superfamily member 6B | 8771 | 0.04542 | -0.001965 |
|  | A0A0E3VY36 | GAST | Gastrin | 2520 | 0.043675 | -0.01317 |
|  | Q16270 | IGFBP7 | Insulin like growth factor binding protein 7 | 3490 | 0.041333 | 0.009988 |
|  | Q8IZP9 | ADGRG2 | Adhesion G protein coupled receptor G2 | 10149 | 0.041082 | -0.00457 |
|  | Q86SJ6 | DSG4 | Desmoglein 4 | 147409 | 0.039043 | -0.002239 |
|  | Q96IQ7 | VSIG2 | V-set and immunoglobulin domain containing 2 | 23584 | 0.031691 | -0.003442 |
|  | B5BUP0 | ACRV1 | Acrosomal protein SP-10 | 56 | 0.031683 | -0.005121 |
| Aortic valve stenosis | None | NTproBNP | N terminal prohormone of brain natriuretic peptide | None | 0.223773 | 0.028766 |
|  | Q14767 | LTBP2 | Latent transforming growth factor beta binding protein 2 | 4053 | 0.213705 | 0.030658 |
|  | A1L4P0 | CNTN5 | Contactin 5 | 53942 | 0.165521 | -0.04252 |
|  | A0A0H3VB22 | FASLG | Tumor necrosis factor ligand superfamily member 6 | 356 | 0.157503 | -0.024058 |
|  | B2R9X8 | MMP12 | Macrophage metalloelastase | 4321 | 0.145415 | -0.009479 |
|  | P10721 | KIT | KIT proto-oncogene, receptor tyrosine kinase | 3815 | 0.143297 | -0.024743 |
|  | F1CME6 | HAVCR1 | Hepatitis A virus cellular receptor 1 | 26762 | 0.137983 | -0.001909 |
|  | A8K144 | BCAN | Brevican | 63827 | 0.107896 | -0.035212 |
|  | O14594 | NCAN | Neurocan | 1463 | 0.107622 | -0.025968 |
|  | P36222 | CHI3L1 | Chitinase 3 like 1 | 1116 | 0.093801 | 0.00421 |
|  | A0A0S2Z517 | ADGRG1 | Adhesion G protein coupled receptor G1 | 9289 | 0.080526 | -0.007267 |
|  | P07998 | RNASE1 | Ribonuclease A family member 1, pancreatic | 6035 | 0.077533 | -0.019466 |
|  | P55291 | CDH15 | Cadherin 15 | 1013 | 0.067759 | -0.002905 |
|  | A0A384MEB1 | HSPB6 | Heat shock protein beta 6 | 126393 | 0.039192 | -0.002056 |
|  | A0A1B0GUZ2 | REN | Renin | 5972 | 0.036202 | 0.004555 |
|  | Q16270 | IGFBP7 | Insulin like growth factor binding protein 7 | 3490 | 0.028959 | 0.006998 |
|  | O14629 | GDF15 | Growth differentiation factor 15 | 9518 | 0.018652 | 0.001827 |
|  | P22692 | IGFBP4 | Insulin like growth factor binding protein 4 | 3487 | 0.017057 | 0.000588 |
| Mitral valve regurgitation | None | NTproBNP | N terminal prohormone of brain natriuretic peptide | None | 0.459136 | 0.059023 |
|  | P47972 | NPTX2 | Neuronal pentraxin 2 | 4885 | 0.148175 | -0.00065 |
|  | F1CME6 | HAVCR1 | Hepatitis A virus cellular receptor 1 | 26762 | 0.146544 | -0.002027 |
|  | A0A384MEB1 | HSPB6 | Heat shock protein beta 6 | 126393 | 0.095757 | -0.005022 |
|  | A0A1B0GUZ2 | REN | Renin | 5972 | 0.082067 | 0.010325 |
|  | O15123 | ANGPT2 | Angiopoietin 2 | 285 | 0.071694 | 0.003214 |
|  | Q14767 | LTBP2 | Latent transforming growth factor beta binding protein 2 | 4053 | 0.068495 | 0.009826 |
|  | A0A384MTN6 | WFDC2 | WAP four disulfide core domain protein 2 | 10406 | 0.039618 | -0.004875 |
|  | O14629 | GDF15 | Growth differentiation factor 15 | 9518 | 0.0355 | -0.003477 |
|  | Q9HAV5 | EDA2R | Tumor necrosis factor receptor superfamily member 27 | 60401 | 0.025684 | 0.00655 |

**Table S2. Cox regression analysis results.**

**Table S3. Net-reclassification indices for multi-omics-based risk prediction model compared to PREVENT model (hold-out validation cohort).**

**Table S4. Net reclassification indices for multi-omics-based risk prediction models compared with the PREVENT model using a 1% 10-year risk threshold (hold-out validation cohort).**

**Table S5. Net reclassification indices for multi-omics-based risk prediction models compared with the PREVENT model using a 2% 10-year risk threshold (hold-out validation cohort).**

**Table S6. The clustering analysis features screened by LASSO regression.**

**Table S7. Clustering feature contribution for three valvular heart disease subtypes.**

**Table S8. Proteins selected by LASSO regression after covariate adjustment.**

| Disease outcome | UniProt ID | Gene name | Protein name | Entrez ID | Mean absolute SHAP value | Mean SHAP Value |
| --- | --- | --- | --- | --- | --- | --- |
| Valvular heart disease | A0A140VJX0 | ACAA1 | Acetyl-CoA acyltransferase 1 | 30 | 0.061172936 | -0.010587756 |
|  | B5BUP0 | ACRV1 | Acrosomal protein SP-10 | 56 | 0.020288976 | -0.002455681 |
|  | A0A8V8TL71 | ACTN4 | Alpha-actinin-4 | 81 | 0.057420688 | 0.015873767 |
|  | A0A669KBE7 | ADAMTSL4 | ADAMTS-like protein 4 | 54507 | 0.050027618 | -0.003052049 |
|  | A8K2Y6 | ADGRE1 | Adhesion G protein-coupled receptor E1 | 2015 | 0.061528359 | -0.002987702 |
|  | A0A0S2Z517 | ADGRG1 | Adhesion G protein-coupled receptor G1 | 9289 | 0.002780352 | 0.000413474 |
|  | A8K4P7 | ADGRG2 | Adhesion G protein-coupled receptor G2 | 10149 | 0.017349135 | 0.001162197 |
|  | Q9BQI0 | AIF1L | Allograft inflammatory factor 1-like protein | 83543 | 0.040775309 | -0.002312005 |
|  | P00977 | AMBP | Protein AMBP | 259 | 0.086630208 | -0.017424943 |
|  | B3KRK1 | AMIGO2 | Amphoterin-induced protein 2 | 347902 | 0.121404792 | 0.036536306 |
|  | P03950 | ANG | Angiogenin | 283 | 0.018085522 | 0.000411894 |
|  | B2R6E3 | ANGPT2 | Angiopoietin-2 | 285 | 0.034843962 | -0.003221663 |
|  | P15144 | ANPEP | Aminopeptidase N | 290 | 0.060945094 | -0.009782358 |
|  | D3DNW7 | ANXA5 | Annexin A5 | 308 | 0.070380959 | 0.006216725 |
|  | B2R677 | AP3S2 | AP-3 complex subunit sigma-2 | 10239 | 0.14575604 | -0.017931972 |
|  | B2R526 | APOC1 | Apolipoprotein C-I | 341 | 0.026282064 | -0.003002305 |
|  | B2R796 | BLMH | Bleomycin hydrolase | 642 | 0.070182378 | -0.007273984 |
|  | A8K2H8 | BPIFB1 | BPI fold-containing family B member 1 | 92747 | 0.072673965 | -0.007549733 |
|  | Q13410 | BTN1A1 | Butyrophilin subfamily 1 member A1 | 696 | 0.042015747 | 0.005673755 |
|  | A0A024RAG6 | C1QA | Complement C1q subcomponent subunit A | 712 | 0.102115979 | -0.001781711 |
|  | A0A3B0ISS0 | C1QTNF9 | C1q and tumor necrosis factor-related protein 9 | 338872 | 0.061441811 | -0.008855258 |
|  | A8K0A5 | CA8 | Carbonic anhydrase-related protein | 767 | 0.032326212 | 0.001042901 |
|  | I3L3R5 | CCER2 | Coiled-coil and glutamate-rich protein 2 | 643669 | 0.081333374 | 0.026253038 |
|  | A0AAQ5BHQ1 | CD200 | Cell surface glycoprotein CD200 | 4345 | 0.071470357 | -0.004411703 |
|  | Q8IX05 | CD302 | CD302 antigen | 9936 | 0.026890391 | 0.000791053 |
|  | A8K664 | CD34 | Hematopoietic progenitor cell antigen CD34 | 947 | 0.05664225 | -0.001132886 |
|  | F8VZE2 | CD63 | CD63 antigen | 967 | 0.121900814 | -0.000268671 |
|  | Q6LET0 | CD82 | CD82 antigen | 3732 | 0.063490729 | -0.017929764 |
|  | B4DT80 | CD8A | T-cell surface glycoprotein CD8 alpha chain | 925 | 0.015571273 | -0.001601255 |
|  | A0A024R0K5 | CEACAM5 | Carcinoembryonic antigen-related cell adhesion molecule 5 | 1048 | 0.030323046 | -0.002499169 |
|  | Q5T2Y7 | CELSR2 | Cadherin EGF LAG seven-pass G-type receptor 2 | 1952 | 0.078317531 | 0.0000861 |
|  | A8K812 | CHAD | Chondroadherin | 1101 | 0.023249052 | 0.002362812 |
|  | B5MBW9 | CHCHD10 | Coiled-coil-helix-coiled-coil-helix domain-containing protein 10 | 400916 | 0.065908262 | -0.002073896 |
|  | A5PKU9 | CHRDL2 | Chordin-like protein 2 | 25884 | 0.092216863 | -0.003252072 |
|  | B3KRP6 | CLEC1A | C-type lectin domain family 1 member A | 51267 | 0.076966981 | -0.007028979 |
|  | E9PHK0 | CLEC3B | Tetranectin | 7123 | 0.003412133 | 0.0000698 |
|  | Q12860 | CNTN1 | Contactin-1 | 1272 | 0.007165885 | -0.000282849 |
|  | A1L4P0 | CNTN5 | Contactin-5 | 53942 | 0.000553406 | 0.0000948 |
|  | B2R800 | CPM | Carboxypeptidase M | 1368 | 0.052760701 | 0.005307077 |
|  | A8MXR7 | CSF3 | Granulocyte colony-stimulating factor | 1440 | 0.05977556 | -0.0073649 |
|  | P28325 | CST5 | Cystatin-D | 1473 | 0.086177545 | -0.014603386 |
|  | B4E2G8 | CTNNA1 | Catenin alpha-1 | 1495 | 0.031272309 | -0.012968995 |
|  | E9PKT6 | CTSH | Pro-cathepsin H | 1512 | 0.052505789 | 0.008025851 |
|  | Q0D2N2 | DDX53 | Cancer/testis antigen 26 | 168400 | 0.055500494 | -0.004857589 |
|  | A0AAQ5BGX8 | DSG2 | Desmoglein-2 | 1829 | 0.033407344 | 0.004056567 |
|  | F2Z328 | DYNLT3 | Dynein light chain Tctex-type 3 | 6990 | 0.082984253 | 0.00425447 |
|  | B2RBZ9 | EDA2R | Ectodysplasin-A2 receptor | 60401 | 0.075094458 | -0.001454268 |
|  | B7WPD3 | ELOB | RNA polymerase II elongation factor B polypeptide | 6923 | 0.06968899 | -0.006065436 |
|  | A1L4C5 | ENTPD5 | Ectonucleoside triphosphate diphosphohydrolase 5 | 957 | 0.031788521 | -0.001291249 |
|  | B4E1D1 | ENTPD6 | Ectonucleoside triphosphate diphosphohydrolase 6 | 955 | 0.062287517 | -0.010053604 |
|  | B4DFE9 | EPHX2 | Bifunctional epoxide hydrolase 2 | 2053 | 0.04142829 | -0.002784112 |
|  | A0A1C9HIH9 | ERMAP | Erythroid membrane-associated protein | 114625 | 0.038971126 | -0.000475207 |
|  | A0A0H3VB22 | FASLG | Tumor necrosis factor ligand superfamily member 6 | 356 | 0.096418835 | -0.0000504 |
|  | B4DU01 | FBN2 | Fibulin-2 | 2201 | 0.047259343 | -0.001557152 |
|  | A0A7U3L5H2 | FGF16 | Fibroblast growth factor 16 | 8823 | 0.056177063 | -0.011006337 |
|  | A0A7U3L649 | FGF20 | Fibroblast growth factor 20 | 26281 | 0.036043168 | -0.002535494 |
|  | A0A0S2Z4C3 | FH | Fumarate hydratase | 2271 | 0.038991107 | -0.015038563 |
|  | E9PD35 | FLT4 | Vascular endothelial growth factor receptor 3 | 2324 | 0.110891285 | -0.012930759 |
|  | A8K4E2 | FOS | Proto-oncogene c-Fos | 2353 | 0.060075749 | -0.005672893 |
|  | A8K8P8 | FUT8 | Alpha-(1,6)-fucosyltransferase | 2530 | 0.019798024 | -0.003313586 |
|  | B2R9C8 | GAS2 | Growth arrest-specific protein 2 | 2620 | 0.036938837 | -0.000410547 |
|  | A0A0E3VY36 | GAST | Gastrin | 2520 | 0.04593505 | 0.004565783 |
|  | O14629 | GDF15 | Growth/differentiation factor 15 | 9518 | 0.0554476 | -0.004088146 |
|  | B2R726 | GPC5 | Glypican-5 | 2262 | 0.020511784 | -0.005933047 |
|  | F1CME6 | HAVCR1 | Hepatitis A virus cellular receptor 1 | 26762 | 0.067088616 | -0.010630727 |
|  | A0A994J6K3 | HEG1 | Protein HEG homolog 1 | 57493 | 0.09087859 | 0.019977968 |
|  | A8MVW5 | HEPACAM2 | HEPACAM family member 2 | 253012 | 0.075958628 | 0.007045459 |
|  | D6RAR4 | HGFAC | Hepatocyte growth factor activator | 3083 | 0.045735206 | -0.009215734 |
|  | P23327 | HRC | Histidine-rich calcium-binding protein | 3270 | 0.046471306 | -0.014354619 |
|  | A0A384MEB1 | HSPB6 | Heat shock protein beta-6 | 126393 | 0.037272474 | 0.000423644 |
|  | Q8TAM9 | ICAM5 | Intercellular adhesion molecule 5 | 7087 | 0.0563022 | 0.006782058 |
|  | Q8TDY8 | IGDCC4 | Immunoglobulin superfamily DCC subclass member 4 | 57722 | 0.002770985 | -0.000126148 |
|  | B1B5Y2 | IGF1R | Insulin-like growth factor 1 receptor | 3480 | 0.064722672 | 0.011150459 |
|  | Q16270 | IGFBP7 | Insulin-like growth factor-binding protein 7 | 3490 | 0.055835621 | 0.005388685 |
|  | A8K6I0 | IL10RA | Interleukin-10 receptor subunit alpha | 3587 | 0.046198396 | -0.002055063 |
|  | Q01638 | IL1RL1 | Interleukin-1 receptor-like 1 | 9173 | 0.014285114 | 0.003197397 |
|  | B4DL40 | IL20RB | Interleukin-20 receptor subunit beta | 53833 | 0.063722785 | -0.017469335 |
|  | A8K9E8 | IL21R | Interleukin-21 receptor | 50615 | 0.038959865 | 0.000771111 |
|  | A0A7R8C389 | IL22 | Interleukin-22 | 50616 | 0.036454368 | -0.005103387 |
|  | B2R9M9 | IL2RA | Interleukin-2 receptor subunit alpha | 3559 | 0.053054142 | -0.007128312 |
|  | Q53FG2 | ITM2A | Integral membrane protein 2A | 9452 | 0.06624717 | 0.005619646 |
|  | A0A0S2Z4K7 | IVD | Isovaleryl-CoA dehydrogenase | 3712 | 0.062212567 | -0.008005442 |
|  | A0A0C4ZN11 | KIR2DS4 | Killer cell immunoglobulin-like receptor 2DS4 | 3809 | 0.062948234 | 0.008358365 |
|  | P49862 | KLK7 | Kallikrein-7 | 5650 | 0.002862952 | 0.000332504 |
|  | B2R874 | KRT19 | Keratin, type I cytoskeletal 19 | 3880 | 0.02444692 | 0.000725944 |
|  | B4DWL3 | LAMP1 | Lysosome-associated membrane glycoprotein 1 | 3916 | 0.089495845 | -0.0053944 |
|  | A0A0U4ARJ3 | LILRA5 | Leukocyte immunoglobulin-like receptor subfamily A member 5 | 353514 | 0.094435399 | 0.005012128 |
|  | P08519 | LPA | Apolipoprotein(a) | 4018 | 0.066778659 | 0.001802965 |
|  | A7E2U6 | LRTM2 | Leucine-rich repeat transmembrane neuronal protein 2 | 654429 | 0.008194647 | 0.00035278 |
|  | Q14767 | LTBP2 | Latent-transforming growth factor beta-binding protein 2 | 4053 | 0.152033732 | -0.018541848 |
|  | A0A0G2JQ91 | MAMDC2 | MAM domain-containing protein 2 | 256691 | 0.050770039 | -0.006933531 |
|  | A0A024RD04 | MAPK13 | Mitogen-activated protein kinase 13 | 5603 | 0.062247416 | 0.004763521 |
|  | A0A0G2JMX7 | MAPT | Microtubule-associated protein tau | 4137 | 0.047154365 | 0.000419964 |
|  | A1A4X9 | MEPE | Matrix extracellular phosphoglycoprotein | 56955 | 0.049533002 | 0.010906425 |
|  | B4E2E6 | METAP1 | Methionine aminopeptidase 1 | 23173 | 0.068442041 | 0.000412515 |
|  | K7ES70 | MFAP4 | Microfibril-associated glycoprotein 4 | 4239 | 0.047973809 | -0.009321412 |
|  | B2R9X8 | MMP12 | Macrophage metalloelastase | 4321 | 0.042790349 | 0.001823144 |
|  | A6ND22 | MRPS16 | 28S ribosomal protein S16, mitochondrial | 51021 | 0.034399779 | 0.000045 |
|  | A1C2E8 | MSTN | Myostatin | 2660 | 0.054254818 | 0.009998726 |
|  | Q6UWD9 | MUC13 | Mucin-13 | 56667 | 0.041454367 | 0.002063097 |
|  | O14594 | NCAN | Neurocan core protein | 1463 | 0.019917635 | -0.002792277 |
|  | B7Z625 | NELL2 | Protein kinase C-binding protein NELL2 | 4753 | 0.028416617 | -0.002824717 |
|  | B5B2S0 | NFATC3 | Nuclear factor of activated T-cells, cytoplasmic 3 | 4775 | 0.075907054 | -0.012100066 |
|  | A8K6I7 | NID2 | Nidogen-2 | 22795 | 0.012720463 | 0.000276233 |
|  | B3VK56 | NOS1 | Nitric oxide synthase, brain | 4842 | 0.027853782 | -0.001270712 |
|  | None | NTproBNP | N terminal prohormone of brain natriuretic peptide | None | 0.288202035 | -0.00926139 |
|  | A0A7P0T8U6 | NOTCH1 | Neurogenic locus notch homolog protein 1 | 4851 | 0.097510548 | 0.00250049 |
|  | A6NDH2 | NPHS1 | Nephrin | 4868 | 0.059625164 | 0.014809758 |
|  | A4D267 | NPTX2 | Neuronal pentraxin-2 | 4885 | 0.039284522 | 0.000409289 |
|  | P34130 | NTF4 | Neurotrophin-4 | 4909 | 0.029164502 | -0.0000116 |
|  | B0ZBE9 | NPPB | Natriuretic peptides B | 4879 | 0.031704605 | 0.002350711 |
|  | Q9NV35 | NUDT15 | nudix hydrolase 15 | 55270 | 0.063127643 | 0.002902036 |
|  | Q5T2G3 | OPTC | Opticin | 26254 | 0.081271218 | -0.002843614 |
|  | B2RCB8 | PCDH12 | Protocadherin-12 | 51294 | 0.090730148 | 0.011118635 |
|  | A0AA34QVH0 | PCSK9 | Proprotein convertase subtilisin/kexin type 9 | 255738 | 0.058524196 | 0.000958307 |
|  | Q8TAE4 | PDE1C | Calcium/calmodulin-dependent 3',5'-cyclic nucleotide phosphodiesterase 1C | 5137 | 0.042732513 | 0.006541765 |
|  | A0A1S5UZ02 | PGA4 | Pepsin A-4 | 643847 | 0.017300353 | 0.001941876 |
|  | Q8IUZ5 | PHYKPL | 5-phosphohydroxy-L-lysine phospho-lyase | 85007 | 0.070730118 | 0.004473903 |
|  | E7ET40 | PLAU | Urokinase-type plasminogen activator | 5328 | 0.049904579 | 0.001608738 |
|  | P01189 | POMC | Pro-opiomelanocortin | 5443 | 0.082524559 | 0.009283606 |
|  | B2R6X6 | PPIF | Peptidyl-prolyl cis-trans isomerase F, mitochondrial | 10105 | 0.057378626 | -0.012794184 |
|  | Q16378 | PRR4 | Proline-rich protein 4 | 11272 | 0.072761984 | 0.004174542 |
|  | A0A184 | PSAPL1 | Prosaposin-like protein 1 | 768239 | 0.075042352 | -0.013978229 |
|  | O75255 | PTPRS | Receptor-type tyrosine-protein phosphatase S | 5802 | 0.082042552 | 0.014227274 |
|  | Q5JT79 | RAB39B | Ras-related protein Rab-39B | 116442 | 0.045610709 | -0.011262707 |
|  | B7Z4W4 | RAD23B | UV excision repair protein RAD23 homolog B | 5887 | 0.120730768 | -0.008109178 |
|  | A0A1B0GUZ2 | REN | Renin | 5972 | 0.040095545 | -0.000729622 |
|  | B4DM41 | RFC4 | Replication factor C subunit 4 | 5984 | 0.050407611 | -0.001906352 |
|  | Q9BSI0 | RGL2 | Ral guanine nucleotide dissociation stimulator-like 2 | 5863 | 0.045390172 | 0.005021204 |
|  | B4DGL9 | RGS8 | Regulator of G-protein signaling 8 | 85397 | 0.057215992 | -0.015646241 |
|  | B2R589 | RNASE1 | Ribonuclease pancreatic | 6035 | 0.04208641 | 0.001527473 |
|  | Q52PI9 | S100A13 | Protein S100-A13 | 6284 | 0.084071852 | 0.005310022 |
|  | A8K5J0 | SBSN | Suprabasin | 374897 | 0.038412339 | 0.011417398 |
|  | Q8WXD2 | SCG3 | Secretogranin-3 | 29106 | 0.003125948 | 0.000107724 |
|  | Q96PL0 | SCGB3A1 | Secretoglobin family 3A member 1 | 92304 | 0.057138491 | 0.00899329 |
|  | A5H1I5 | SCN3B | Sodium channel subunit beta-3 | 55800 | 0.041662802 | 0.000149249 |
|  | A0A3G1HEF1 | SERPINA7 | Thyroxine-binding globulin | 6906 | 0.040160409 | -0.005511704 |
|  | B2R6Y4 | SERPINB5 | Serpin B5 | 5268 | 0.046319476 | 0.001793052 |
|  | A0A7H0TJC6 | SERPIND1 | Heparin cofactor 2 | 3053 | 0.079679423 | -0.018845043 |
|  | A0A0S2Z6B4 | SIL1 | Nucleotide exchange factor SIL1 | 64374 | 0.046990078 | 0.007720991 |
|  | P43007 | SLC1A4 | Neutral amino acid transporter A | 6509 | 0.045001529 | -0.009688788 |
|  | A0A0G2JL76 | SLC44A4 | Choline transporter-like protein 4 | 80736 | 0.063737711 | 0.021430309 |
|  | B4DWS3 | SPINK5 | Serine protease inhibitor Kazal-type 5 | 11005 | 0.022373431 | 0.006899911 |
|  | B2R5H7 | SPRR1B | Cornifin-B | 6699 | 0.043105562 | 0.003315057 |
|  | A0A140VJW3 | SUSD2 | Sushi domain-containing protein 2 | 56241 | 0.068433445 | 0.004718808 |
|  | C5IX07 | TACSTD2 | Tumor-associated calcium signal transducer 2 | 4070 | 0.054140377 | 0.008801662 |
|  | P10646 | TFPI | Tissue factor pathway inhibitor | 7035 | 0.073067897 | 0.009543438 |
|  | A0A3B3IS67 | TFPI2 | Tissue factor pathway inhibitor 2 | 7980 | 0.065151719 | 0.007146992 |
|  | A0A3B3ITZ9 | THRAP3 | Thyroid hormone receptor-associated protein 3 | 9967 | 0.054813687 | 0.021457317 |
|  | O95407 | TNFRSF6B | Tumor necrosis factor receptor superfamily member 6B | 8771 | 0.01265554 | 0.000556723 |
|  | Q07011 | TNFRSF9 | Tumor necrosis factor receptor superfamily member 9 | 3604 | 0.085756093 | -0.002658179 |
|  | B6D427 | TNNI3 | Troponin I, cardiac muscle | 7137 | 0.072069158 | -0.007464506 |
|  | A0A386NC20 | TP53 | Cellular tumor antigen p53 | 7157 | 0.103377844 | 0.005126699 |
|  | B7Z3E7 | TP53BP1 | p53-binding protein 1 | 7158 | 0.057366734 | 0.001941237 |
|  | F5GZG6 | TPK1 | Thiamin pyrophosphokinase 1 | 27010 | 0.108130066 | 0.001632333 |
|  | Q08AP8 | TREML2 | Trem-like transcript 2 protein | 79865 | 0.077201176 | 0.013389518 |
|  | A0A0F7RQP6 | TSHB | Thyrotropin subunit beta | 7252 | 0.026330904 | 0.000866014 |
|  | A0A140VJZ3 | UBQLN3 | Ubiquilin-3 | 50613 | 0.081982039 | -0.00524134 |
|  | Q5VUN4 | ULBP2 | UL16-binding protein 2 | 80328 | 0.062212936 | -0.001869188 |
|  | B3KP48 | UMOD | Uromodulin | 7369 | 0.009525755 | 0.000844781 |
|  | A3KMF8 | UPB1 | Beta-ureidopropionase | 51733 | 0.060763261 | -0.016791417 |
|  | A8K3Q3 | UXS1 | UDP-glucuronic acid decarboxylase 1 | 80146 | 0.048677231 | -0.006894512 |
|  | P13611 | VCAN | Versican core protein | 1462 | 0.073026115 | 0.007803921 |
|  | Q86VR7 | VSIG10L | V-set and immunoglobulin domain-containing protein 10-like | 147645 | 0.038489578 | -0.00529779 |
|  | Q96IQ7 | VSIG2 | V-set and immunoglobulin domain-containing protein 2 | 23584 | 0.012546892 | 0.001994487 |
|  | A8K6N3 | VWA5A | Von Willebrand factor A domain-containing protein 5A | 4013 | 0.054936454 | 0.004276267 |
|  | A6NC69 | VWC2L | Von Willebrand factor C domain-containing protein 2-like | 402117 | 0.04756395 | 0.005235507 |
|  | Q9UPY6 | WASF3 | Wiskott-Aldrich syndrome protein family member 3 | 10810 | 0.058918149 | 0.003610461 |
|  | A0A384MTN6 | WFDC2 | WAP four-disulfide core domain protein 2 | 10406 | 0.00837018 | -0.000926892 |
|  | B4DX53 | WIF1 | Wnt inhibitory factor 1 | 11197 | 0.040106445 | -0.001118334 |
|  | A6NLW2 | WNT9A | Protein Wnt-9a | 7483 | 0.106759679 | 0.002997041 |
|  | A0A024R3C6 | ZBTB16 | Zinc finger and BTB domain-containing protein 16 | 7704 | 0.14126247 | -0.003694978 |
|  | P51815 | ZNF75D | Zinc finger protein 75D | 7626 | 0.064132842 | 0.002895883 |
| Aortic valve stenosis | Q5FWF1 | ADAMTS8 | ADAM metallopeptidase with thrombospondin type 1 motif 8 | 11095 | 0.080688973 | 0.007604439 |
|  | A0A0S2Z517 | ADGRG1 | Adhesion G protein-coupled receptor G1 | 9289 | 0.006894489 | 0.0010253 |
|  | A8K4P7 | ADGRG2 | Adhesion G protein-coupled receptor G2 | 10149 | 0.008019577 | 0.000537222 |
|  | A8K144 | BCAN | Brevican core protein | 63827 | 0.058105708 | 0.005048822 |
|  | Q13410 | BTN1A1 | Butyrophilin subfamily 1 member A1 | 696 | 0.087179903 | 0.011772667 |
|  | B0QY51 | CCDC134 | Coiled-coil domain-containing protein 134 | 79879 | 0.109566124 | 0.010323229 |
|  | I3L3R5 | CCER2 | Coiled-coil and glutamate-rich protein 2 | 643669 | 0.096984569 | 0.031304979 |
|  | A0AAQ5BHQ1 | CD200 | Cell surface glycoprotein CD200 | 4345 | 0.081780208 | -0.005048107 |
|  | P55291 | CDH15 | Cadherin-15 | 1013 | 0.019570006 | 0.002441544 |
|  | B2R7B0 | CHI3L1 | Chitinase-3-like protein 1 | 1116 | 0.023973754 | -0.001115331 |
|  | E9PHK0 | CLEC3B | Tetranectin | 7123 | 0.059881915 | -0.001225192 |
|  | A1L4P0 | CNTN5 | Contactin-5 | 53942 | 0.097378718 | -0.016677035 |
|  | Q0GLB7 | DPP10 | Dipeptidyl peptidase 10 | 57628 | 0.145111587 | -0.028550878 |
|  | B0BCZ6 | ENPEP | Glutamyl aminopeptidase | 2028 | 0.052892853 | 0.001723336 |
|  | P21860 | ERBB3 | Receptor tyrosine-protein kinase erbB-3 | 2065 | 0.13330496 | -0.025123712 |
|  | A0A0H3VB22 | FASLG | Tumor necrosis factor ligand superfamily member 6 | 356 | 0.112413252 | -0.0000587 |
|  | A6NKU4 | FGL1 | Fibrinogen-like protein 1 | 2267 | 0.125967605 | -0.01088355 |
|  | E9PD35 | FLT4 | Vascular endothelial growth factor receptor 3 | 2324 | 0.139375972 | -0.016252287 |
|  | O14629 | GDF15 | Growth/differentiation factor 15 | 9518 | 0.080050403 | -0.005902108 |
|  | F1CME6 | HAVCR1 | Hepatitis A virus cellular receptor 1 | 26762 | 0.101829611 | -0.016135715 |
|  | A0A994J6K3 | HEG1 | Protein HEG homolog 1 | 57493 | 0.140957988 | 0.030986992 |
|  | A0N9W2 | IGFBP4 | Insulin-like growth factor-binding protein 4 | 3487 | 0.029744445 | 0.002798855 |
|  | Q16270 | IGFBP7 | Insulin-like growth factor-binding protein 7 | 3490 | 0.070436898 | 0.006797852 |
|  | A0A7R8C389 | IL22 | Interleukin-22 | 50616 | 0.043579347 | -0.00610084 |
|  | A0A8I5KPX7 | KIT | Mast/stem cell growth factor receptor Kit | 3815 | 0.026119404 | 0.000583022 |
|  | Q14767 | LTBP2 | Latent-transforming growth factor beta-binding protein 2 | 4053 | 0.201793802 | -0.024610525 |
|  | P12872 | MLN | Motilin | 4295 | 0.079726045 | 0.010133331 |
|  | B2R9X8 | MMP12 | Macrophage metalloelastase | 4321 | 0.07117282 | 0.003032419 |
|  | B4DDJ5 | MSR1 | Macrophage scavenger receptor types I and II | 4481 | 0.054831928 | 0.003386144 |
|  | O14594 | NCAN | Neurocan core protein | 1463 | 0.058728102 | -0.008233162 |
|  | None | NTproBNP | N terminal prohormone of brain natriuretic peptide | None | 0.211498767 | -0.006796526 |
|  | Q9NV35 | NUDT15 | nudix hydrolase 15 | 55270 | 0.096522046 | 0.004437208 |
|  | Q5T2G3 | OPTC | Opticin | 26254 | 0.085639598 | -0.00299646 |
|  | O43342 | PRSS22 | Serine protease 22 | 64063 | 0.057662157 | -0.002922131 |
|  | B4DWP2 | PRSS8 | Prostasin | 5652 | 0.055445955 | 0.007441461 |
|  | A0A184 | PSAPL1 | Prosaposin-like protein 1 | 768239 | 0.137964347 | -0.025698785 |
|  | O75255 | PTPRS | Receptor-type tyrosine-protein phosphatase S | 5802 | 0.077360837 | 0.013415402 |
|  | A0A1B0GUZ2 | REN | Renin | 5972 | 0.006054169 | -0.000110168 |
|  | B4DGL9 | RGS8 | Regulator of G-protein signaling 8 | 85397 | 0.130475427 | -0.035679711 |
|  | B2R589 | RNASE1 | Ribonuclease pancreatic | 6035 | 0.032506017 | 0.001179765 |
|  | A8K5J0 | SBSN | Suprabasin | 374897 | 0.182398622 | 0.054214809 |
|  | A0A140VJW3 | SUSD2 | Sushi domain-containing protein 2 | 56241 | 0.129624056 | 0.008938188 |
|  | B0AZS4 | TBR1 | T-box brain protein 1 | 10716 | 0.12562967 | -0.022756034 |
|  | P04155 | TFF1 | Trefoil factor 1 | 7031 | 0.109279075 | -0.011510084 |
|  | O95407 | TNFRSF6B | Tumor necrosis factor receptor superfamily member 6B | 8771 | 0.070244916 | 0.003090106 |
|  | B6D427 | TNNI3 | Troponin I, cardiac muscle | 7137 | 0.126087726 | -0.013059437 |
|  | A0A140TA52 | TNXB | Tenascin-X | 7148 | 0.095172894 | 0.002461408 |
| Mitral valve regurgitation | P26442 | AMFR | Autocrine motility factor receptor | 267 | 0.121605119 | -0.035674192 |
|  | B3KRK1 | AMIGO2 | Amphoterin-induced protein 2 | 347902 | 0.082732604 | 0.024898059 |
|  | B2R6E3 | ANGPT2 | Angiopoietin-2 | 285 | 0.064935121 | -0.006003884 |
|  | P15144 | ANPEP | Aminopeptidase N | 290 | 0.117619086 | -0.018879157 |
|  | B4E100 | APBB1IP | Amyloid beta A4 precursor protein-binding family B member 1-interacting protein | 54518 | 0.065477804 | 0.004270646 |
|  | B2R526 | APOC1 | Apolipoprotein C-I | 341 | 0.043016634 | -0.004913961 |
|  | B2R4E2 | ATP5PO | ATP synthase subunit O, mitochondrial | 539 | 0.14024023 | -0.00768902 |
|  | Q5VU50 | BSND | Barttin | 7809 | 0.074826045 | -0.015459187 |
|  | A8K2T4 | C7 | Complement component C7 | 730 | 0.031700949 | 0.002605943 |
|  | I3L3R5 | CCER2 | Coiled-coil and glutamate-rich protein 2 | 643669 | 0.081333956 | 0.026253225 |
|  | B4DT80 | CD8A | T-cell surface glycoprotein CD8 alpha chain | 925 | 0.11876496 | -0.012213065 |
|  | Q13231 | CHIT1 | Chitotriosidase-1 | 1118 | 0.066592714 | 0.004564308 |
|  | Q12860 | CNTN1 | Contactin-1 | 1272 | 0.036795786 | 0.001452387 |
|  | P28325 | CST5 | Cystatin-D | 1473 | 0.11094667 | -0.018800687 |
|  | A0A024RDB7 | CXCL13 | C-X-C motif chemokine 13 | 10563 | 0.031860768 | -0.000113184 |
|  | B4E3V1 | DDAH1 | N(G),N(G)-dimethylarginine dimethylaminohydrolase 1 | 23576 | 0.058459821 | 0.010937753 |
|  | B7WPD3 | ELOB | RNA polymerase II elongation factor B polypeptide | 6923 | 0.058180644 | -0.005063798 |
|  | P12104 | FABP2 | Fatty acid-binding protein, intestinal | 2169 | 0.085797867 | 0.003618571 |
|  | Q0Z7S8 | FABP9 | Fatty acid-binding protein 9 | 646480 | 0.110333038 | 0.008871249 |
|  | B7ZVY3 | GAGE2A | G antigen 2A | 729447 | 0.056008109 | -0.009632219 |
|  | O14629 | GDF15 | Growth/differentiation factor 15 | 9518 | 0.106339418 | -0.007840395 |
|  | P09681 | GIP | Gastric inhibitory polypeptide | 2695 | 0.056977808 | 0.0000331 |
|  | A6NFM9 | GP2 | Glycoprotein 2 | 2813 | 0.07279737 | -0.004042238 |
|  | F1CME6 | HAVCR1 | Hepatitis A virus cellular receptor 1 | 26762 | 0.10182408 | -0.016134838 |
|  | A0A384MEB1 | HSPB6 | Heat shock protein beta-6 | 126393 | 0.08531624 | -0.000969715 |
|  | Q8TAM9 | ICAM5 | Intercellular adhesion molecule 5 | 7087 | 0.077238551 | 0.009304012 |
|  | Q8TDY8 | IGDCC4 | Immunoglobulin superfamily DCC subclass member 4 | 57722 | 0.030985638 | -0.00141061 |
|  | B1B5Y2 | IGF1R | Insulin-like growth factor 1 receptor | 3480 | 0.058024561 | 0.009996505 |
|  | Q16270 | IGFBP7 | Insulin-like growth factor-binding protein 7 | 3490 | 0.041452421 | 0.004000565 |
|  | B0YJ28 | IL18 | Interleukin-18 | 3606 | 0.032311768 | -0.00279208 |
|  | Q9NZH8 | IL36G | Interleukin-36 gamma | 56300 | 0.076104014 | 0.005394285 |
|  | P05113 | IL5 | Interleukin-5 | 3567 | 0.101811477 | 0.000920947 |
|  | P49862 | KLK7 | Kallikrein-7 | 5650 | 0.024843912 | 0.002885375 |
|  | A8K6Q2 | LRRN1 | Leucine-rich repeat neuronal protein 1 | 57633 | 0.008129255 | 0.0000949 |
|  | Q14767 | LTBP2 | Latent-transforming growth factor beta-binding protein 2 | 4053 | 0.038404315 | -0.004683743 |
|  | A0A0G2JQ91 | MAMDC2 | MAM domain-containing protein 2 | 256691 | 0.05094604 | -0.006957567 |
|  | A1A4X9 | MEPE | Matrix extracellular phosphoglycoprotein | 56955 | 0.075759025 | 0.016681002 |
|  | None | NTproBNP | N terminal prohormone of brain natriuretic peptide | None | 0.366693577 | -0.01178372 |
|  | O14594 | NCAN | Neurocan core protein | 1463 | 0.078593319 | -0.011018091 |
|  | B0ZBE9 | NPPB | Natriuretic peptides B | 4879 | 0.058153465 | 0.004311739 |
|  | A4D267 | NPTX2 | Neuronal pentraxin-2 | 4885 | 0.094191589 | 0.000981344 |
|  | A4D0S3 | NRCAM | Neuronal cell adhesion molecule | 4897 | 0.078595893 | 0.004345715 |
|  | B2RPE8 | NRTN | Neurturin | 4902 | 0.101413325 | -0.012791252 |
|  | P01189 | POMC | Pro-opiomelanocortin | 5443 | 0.097813955 | 0.011003588 |
|  | O75255 | PTPRS | Receptor-type tyrosine-protein phosphatase S | 5802 | 0.073302658 | 0.01271166 |
|  | A0A1B0GUZ2 | REN | Renin | 5972 | 0.050739749 | -0.000923315 |
|  | A0A3G1HEF1 | SERPINA7 | Thyroxine-binding globulin | 6906 | 0.06266084 | -0.008599713 |
|  | B2R6Y4 | SERPINB5 | Serpin B5 | 5268 | 0.101497089 | 0.003929008 |
|  | A0A7H0TJC6 | SERPIND1 | Heparin cofactor 2 | 3053 | 0.08884772 | -0.021013444 |
|  | B4DYC1 | SFRP4 | Secreted frizzled-related protein 4 | 6424 | 0.07500226 | 0.016454599 |
|  | A4D247 | SHH | Sonic hedgehog protein | 6469 | 0.059985892 | 0.003566887 |
|  | B4DWS3 | SPINK5 | Serine protease inhibitor Kazal-type 5 | 11005 | 0.078344222 | 0.024161165 |
|  | A0A3B3ITZ9 | THRAP3 | Thyroid hormone receptor-associated protein 3 | 9967 | 0.075609787 | 0.029598139 |
|  | O14720 | TNFRSF10B | Tumor necrosis factor receptor superfamily member 10B | 8795 | 0.027856977 | 0.000479858 |
|  | P17643 | TYRP1 | Tyrosinase-related protein 1 | 7306 | 0.113465773 | 0.002033038 |
|  | A0A140VJZ3 | UBQLN3 | Ubiquilin-3 | 50613 | 0.104165288 | -0.006659577 |
|  | B3KP48 | UMOD | Uromodulin | 7369 | 0.078089557 | -0.006925286 |
|  | Q96IQ7 | VSIG2 | V-set and immunoglobulin domain-containing protein 2 | 23584 | 0.050943408 | -0.0080981 |
|  | A8K6N3 | VWA5A | Von Willebrand factor A domain-containing protein 5A | 4013 | 0.060239775 | 0.004689079 |
|  | A0A384MTN6 | WFDC2 | WAP four-disulfide core domain protein 2 | 10406 | 0.045943029 | -0.005087614 |

**Table S9. KEGG pathway analysis results.**

**Table S10. GO Enrichment Analysis of Shared and Subtype-Specific Protein Predictors.**

**Table S11. Instrumental variables for plasma proteins.**

| **PROTEIN** | **RSID** | **CHROM** | **POS** | **ALLELE0** | **ALLELE1** | **A1FREQ** | **N** | **BETA** | **SE** | **PVAL** | **F_STATISTIC** |
| --- | --- | --- | --- | --- | --- | --- | --- | --- | --- | --- | --- |
| NPPB | rs198389 | 1 | 11859214 | A | G | 0.12 | 30,931 | 0.23 | 0.02 | 3.00E-77 | 17.12 |
| CNTN5 | rs282486 | 11 | 99337409 | A | G | 0.09 | 5,335 | 0.25 | 0.03 | 5.80E-18 | 34.93 |
| CNTN5 | rs6589899 | 11 | 98987273 | G | A | 0.07 | 5,335 | 0.23 | 0.03 | 1.54E-12 | 42.84 |
| CNTN5 | rs7113594 | 11 | 99044623 | A | G | 0.08 | 5,335 | 0.26 | 0.03 | 1.13E-16 | 407.98 |
| HAVCR1 | rs156085 | 5 | 139542437 | G | A | 0.07 | 5,367 | 0.07 | 0.22 | 5.92E-09 | 32.61 |
| HAVCR1 | rs78486606 | 5 | 139605396 | A | G | 0.07 | 5,367 | 0.07 | 0.22 | 1.01E-08 | 395.77 |
| HAVCR1 | rs11558407 | 5 | 139625224 | G | A | 0.06 | 5,367 | 0.06 | 0.24 | 7.17E-09 | 45.53 |
| HAVCR1 | rs76710271 | 5 | 153973096 | T | C | 0.10 | 5,367 | 0.10 | -0.18 | 6.15E-09 | 82.50 |
| HAVCR1 | rs77851120 | 5 | 153973152 | G | T | 0.10 | 5,367 | 0.10 | -0.18 | 6.15E-09 | 90.21 |
| HAVCR1 | rs12513761 | 5 | 153979566 | C | A | 0.10 | 5,367 | 0.10 | -0.18 | 1.31E-08 | 186.55 |
| HAVCR1 | rs12516510 | 5 | 153979643 | T | A | 0.10 | 5,367 | 0.10 | -0.18 | 1.31E-08 | 159.61 |
| HAVCR1 | rs72809694 | 5 | 155600815 | A | G | 0.02 | 5,367 | 0.02 | -0.40 | 2.10E-08 | 98.27 |
| HAVCR1 | rs72811723 | 5 | 155627398 | A | G | 0.03 | 5,367 | 0.03 | -0.37 | 3.39E-10 | 124.39 |
| HAVCR1 | rs17053386 | 5 | 155709100 | G | A | 0.03 | 5,367 | 0.03 | -0.32 | 3.01E-08 | 84.51 |
| HAVCR1 | rs191192726 | 5 | 155787762 | C | T | 0.02 | 5,367 | 0.02 | 0.35 | 1.02E-08 | 212.33 |
| HAVCR1 | rs140010034 | 5 | 155794558 | A | G | 0.02 | 5,367 | 0.02 | 0.37 | 2.19E-08 | 67.01 |
| HAVCR1 | rs72798955 | 5 | 155809527 | A | G | 0.02 | 5,367 | 0.02 | -0.41 | 1.34E-08 | 62.94 |
| HAVCR1 | rs190584164 | 5 | 155845153 | G | A | 0.02 | 5,367 | 0.02 | 0.35 | 9.52E-09 | 47.55 |
| HAVCR1 | rs72799001 | 5 | 155870470 | G | T | 0.03 | 5,367 | 0.03 | -0.29 | 2.23E-08 | 212.95 |
| HAVCR1 | rs147309548 | 5 | 155885884 | T | C | 0.02 | 5,367 | 0.02 | 0.38 | 8.61E-10 | 57.44 |
| HAVCR1 | rs184717581 | 5 | 155893570 | C | T | 0.02 | 5,367 | 0.02 | 0.35 | 1.38E-08 | 42.20 |
| BCAN | rs59382478 | 1 | 156607586 | T | C | 0.02 | 5,366 | 0.57 | 0.07 | 9.52E-15 | 1915.27 |
| BCAN | rs2365715 | 1 | 156615114 | A | G | 0.38 | 5,366 | -0.23 | 0.02 | 2.87E-31 | 38.65 |
| BCAN | rs728918 | 1 | 156504728 | G | A | 0.22 | 5,366 | -0.17 | 0.02 | 2.55E-13 | 55.06 |
| IGFBP7 | rs7692969 | 4 | 57905048 | T | C | 0.50 | 5,355 | 0.10 | 0.02 | 2.56E-08 | 53.28 |
| IGFBP7 | rs1718860 | 4 | 57949517 | A | G | 0.30 | 5,355 | 0.26 | 0.02 | 2.88E-42 | 68.89 |
| IGFBP7 | rs781560 | 4 | 57714846 | G | A | 0.16 | 5,355 | 0.14 | 0.02 | 9.44E-12 | 82.98 |
| IGFBP7 | rs184325393 | 4 | 56011047 | C | T | 0.01 | 5,355 | 0.43 | 0.08 | 1.73E-08 | 48.69 |
| IGFBP7 | rs144644158 | 4 | 57615592 | T | C | 0.01 | 5,355 | 0.61 | 0.09 | 6.99E-12 | 135.08 |
| GDF15 | rs1058587 | 19 | 18499422 | C | G | 0.24 | 5,368 | 0.41 | 0.02 | 1.40E-98 | 53.65 |
| GDF15 | rs28406304 | 19 | 19252677 | C | T | 0.04 | 5,368 | 0.25 | 0.05 | 1.85E-08 | 398.24 |
| GDF15 | rs72620570 | 19 | 18413024 | C | T | 0.09 | 5,368 | 0.24 | 0.03 | 1.34E-16 | 136.68 |
| CD200 | rs62275482 | 3 | 111316801 | G | A | 0.09 | 5,363 | -0.19 | 0.03 | 3.37E-09 | 86.15 |
| CD200 | rs73214109 | 3 | 111414955 | G | A | 0.03 | 5,363 | -0.30 | 0.05 | 1.57E-08 | 46.68 |
| CD200 | rs75070661 | 3 | 111448991 | G | A | 0.05 | 5,363 | -0.26 | 0.04 | 2.59E-09 | 203.18 |
| CD200 | rs140294958 | 3 | 111476323 | A | G | 0.03 | 5,363 | -0.29 | 0.05 | 2.29E-08 | 186.54 |
| CD200 | rs115337976 | 3 | 111560973 | T | C | 0.02 | 5,363 | -0.39 | 0.06 | 6.51E-10 | 41.84 |
| CD200 | rs76403048 | 3 | 111571129 | G | A | 0.03 | 5,363 | -0.35 | 0.06 | 4.66E-10 | 36.94 |
| CD200 | rs77975132 | 3 | 111577900 | T | A | 0.03 | 5,363 | -0.35 | 0.06 | 4.66E-10 | 32.20 |
| CD200 | rs74640145 | 3 | 111692041 | A | C | 0.03 | 5,363 | -0.37 | 0.06 | 5.56E-11 | 36.30 |
| CD200 | rs76279680 | 3 | 111702068 | G | A | 0.07 | 5,363 | -0.20 | 0.04 | 3.16E-08 | 45.67 |
| FGL1 | rs426294 | 8 | 17617494 | C | T | 0.40 | 5,368 | -0.11 | 0.02 | 2.61E-09 | 67.81 |
| FGL1 | rs388048 | 8 | 17617535 | T | C | 0.21 | 5,368 | -0.19 | 0.02 | 2.66E-17 | 122.59 |
| FGL1 | rs368531 | 8 | 17617889 | G | A | 0.39 | 5,368 | -0.11 | 0.02 | 2.99E-09 | 29.90 |
| FGL1 | rs428315 | 8 | 17618886 | C | T | 0.20 | 5,368 | -0.20 | 0.02 | 2.96E-18 | 552.18 |
| FGL1 | rs595764 | 8 | 17619617 | C | T | 0.20 | 5,368 | -0.20 | 0.02 | 4.45E-18 | 44.39 |
| FGL1 | rs451322 | 8 | 17620942 | A | G | 0.43 | 5,368 | -0.11 | 0.02 | 1.53E-08 | 63.83 |
| FLT4 | rs13081582 | 3 | 94495036 | C | A | 0.01 | 5,365 | -0.25 | 0.04 | 1.77E-08 | 28.44 |
| FLT4 | rs12496615 | 3 | 97784441 | G | A | 0.05 | 5,365 | 0.13 | 0.02 | 8.17E-10 | 39.40 |
| FLT4 | rs6764595 | 3 | 97784559 | C | T | 0.01 | 5,365 | 0.26 | 0.04 | 1.24E-08 | 38.61 |
| FLT4 | rs1826351 | 3 | 97787260 | A | C | 0.04 | 5,365 | -0.15 | 0.02 | 1.55E-08 | 645.51 |
| FLT4 | rs6764022 | 3 | 97799234 | G | T | 0.07 | 5,365 | -0.12 | 0.02 | 2.92E-08 | 251.88 |
| FLT4 | rs4857315 | 3 | 97808108 | T | C | 0.22 | 5,365 | -0.08 | 0.01 | 1.22E-08 | 29.92 |
| FLT4 | rs4241465 | 3 | 97808389 | T | G | 0.48 | 5,365 | 0.07 | 0.01 | 1.51E-08 | 96.37 |
| FLT4 | rs4557200 | 3 | 97808505 | A | C | 0.04 | 5,365 | -0.18 | 0.02 | 1.51E-08 | 73.96 |
| FLT4 | rs4241466 | 3 | 97808542 | T | A | 0.03 | 5,365 | 0.22 | 0.03 | 1.51E-08 | 24.17 |
| FLT4 | rs9289515 | 3 | 97808936 | A | C | 0.07 | 5,365 | -0.14 | 0.01 | 1.38E-08 | 20.34 |
| FLT4 | rs62269696 | 3 | 97808994 | G | A | 0.42 | 5,365 | -0.08 | 0.01 | 9.31E-14 | 28.03 |
| FLT4 | rs9289516 | 3 | 97809100 | C | T | 0.08 | 5,365 | 0.15 | 0.01 | 1.46E-08 | 28.57 |
| FLT4 | rs9289517 | 3 | 97809404 | T | A | 0.07 | 5,365 | -0.16 | 0.02 | 1.99E-08 | 25.73 |
| FLT4 | rs10935196 | 3 | 97809601 | T | G | 0.01 | 5,365 | -0.45 | 0.04 | 2.16E-08 | 1290.58 |
| FLT4 | rs112853066 | 3 | 97810310 | C | A | 0.01 | 5,365 | 0.53 | 0.05 | 2.63E-08 | 80.27 |
| FLT4 | rs111633718 | 3 | 97810439 | T | C | 0.01 | 5,365 | -0.46 | 0.04 | 2.46E-08 | 24.88 |
| FLT4 | rs6800892 | 3 | 97810829 | T | C | 0.00 | 5,365 | -0.76 | 0.06 | 3.05E-08 | 28.41 |
| FLT4 | rs6439608 | 3 | 97811764 | T | C | 0.09 | 5,365 | 0.18 | 0.01 | 2.50E-08 | 102.02 |
| FLT4 | rs7633730 | 3 | 97811984 | A | C | 0.02 | 5,365 | -0.41 | 0.03 | 2.21E-08 | 1062.11 |
| FLT4 | rs6804072 | 3 | 97812319 | C | T | 0.02 | 5,365 | -0.46 | 0.03 | 2.24E-08 | 23.99 |
| FLT4 | rs6789360 | 3 | 97812567 | A | C | 0.01 | 5,365 | -0.90 | 0.05 | 2.24E-08 | 40.57 |
| FLT4 | rs6439611 | 3 | 97813003 | T | C | 0.03 | 5,365 | 0.50 | 0.02 | 2.36E-08 | 39.63 |
| FLT4 | rs6439612 | 3 | 97813392 | G | A | 0.42 | 5,365 | 0.17 | 0.01 | 2.36E-08 | 53.85 |
| FLT4 | rs4490406 | 3 | 97813615 | G | A | 0.03 | 5,365 | 0.50 | 0.02 | 2.36E-08 | 1520.37 |
| MLN | s16862260 | 2 | 222069957 | G | A | 0.24 | 5,368 | -0.25 | 0.02 | 7.17E-31 | 216.96 |
| MLN | rs59401206 | 2 | 222052551 | A | G | 0.25 | 5,368 | -0.24 | 0.02 | 3.49E-28 | 32.96 |
| MLN | rs35612982 | 6 | 20682622 | T | C | 0.14 | 5,368 | 0.25 | 0.03 | 7.02E-20 | 201.61 |
| MLN | rs1737024 | 6 | 29741592 | G | C | 0.23 | 5,368 | 0.13 | 0.02 | 2.10E-09 | 238.13 |
| MLN | rs9274514 | 6 | 32634243 | G | A | 0.14 | 5,368 | 0.16 | 0.03 | 1.71E-08 | 54.14 |
| MLN | rs112779317 | 6 | 33640243 | C | A | 0.05 | 5,368 | 0.31 | 0.04 | 5.49E-12 | 200.63 |
| MLN | rs73412140 | 6 | 33757014 | G | T | 0.27 | 5,368 | -0.47 | 0.02 | 5.31E-113 | 56.00 |
| MLN | rs12195485 | 6 | 33790877 | C | T | 0.22 | 5,368 | 0.20 | 0.02 | 2.29E-18 | 131.76 |
| MLN | rs35387652 | 6 | 33813095 | T | G | 0.19 | 5,368 | 0.25 | 0.02 | 1.71E-25 | 37.65 |
| MLN | rs6931760 | 6 | 33813971 | G | C | 0.24 | 5,368 | 0.19 | 0.02 | 4.73E-17 | 135.55 |
| MLN | rs10947457 | 6 | 33814147 | C | T | 0.19 | 5,368 | 0.25 | 0.02 | 2.06E-23 | 54.81 |
| MLN | rs6917634 | 6 | 33815117 | T | C | 0.24 | 5,368 | 0.19 | 0.02 | 5.08E-17 | 70.75 |
| MLN | rs76766228 | 10 | 67772132 | G | A | 0.03 | 5,368 | 0.31 | 0.06 | 4.14E-08 | 183.40 |
| MLN | rs734307 | 12 | 98791382 | C | T | 0.45 | 5,368 | -0.11 | 0.02 | 4.92E-09 | 84.39 |
| MLN | rs602662 | 19 | 49206985 | A | G | 0.38 | 5,368 | 0.11 | 0.02 | 7.82E-09 | 387.16 |
| MLN | rs485073 | 19 | 49207255 | G | A | 0.38 | 5,368 | 0.11 | 0.02 | 1.04E-08 | 51.77 |
| MLN | rs6034694 | 20 | 16932592 | A | T | 0.46 | 5,368 | -0.13 | 0.02 | 8.95E-12 | 3582.95 |
| MLN | rs11700038 | 20 | 17052919 | G | C | 0.47 | 5,368 | -0.10 | 0.02 | 3.42E-08 | 105.73 |
| MLN | rs2424024 | 20 | 16953692 | C | A | 0.05 | 5,368 | -0.27 | 0.04 | 5.79E-10 | 91.80 |
| MLN | rs1883551 | 20 | 16955283 | T | C | 0.10 | 5,368 | -0.19 | 0.03 | 2.58E-09 | 80.77 |
| MLN | rs2424026 | 20 | 16956638 | A | G | 0.21 | 5,368 | -0.15 | 0.02 | 8.18E-11 | 39.46 |
| MLN | rs852024 | 20 | 17081763 | G | T | 0.47 | 5,368 | -0.10 | 0.02 | 4.54E-08 | 40.01 |
| PSAPL1 | rs644591 | 1 | 41996058 | G | T | 0.04 | 5,367 | -0.26 | 0.05 | 3.14E-08 | 140.24 |
| PSAPL1 | rs144486945 | 1 | 216276590 | G | A | 0.01 | 5,367 | 0.44 | 0.08 | 4.58E-08 | 39.74 |
| PSAPL1 | rs77370429 | 4 | 7168880 | C | T | 0.02 | 5,367 | -0.30 | 0.05 | 3.70E-08 | 175.85 |
| PSAPL1 | rs74574444 | 4 | 7169275 | G | C | 0.02 | 5,367 | -0.31 | 0.06 | 2.40E-08 | 48.12 |
| PSAPL1 | rs7681193 | 4 | 7382543 | G | T | 0.48 | 5,367 | 0.11 | 0.02 | 5.55E-10 | 323.58 |
| PSAPL1 | rs2005355 | 4 | 7383395 | G | A | 0.50 | 5,367 | 0.11 | 0.02 | 2.11E-11 | 114.69 |
| PSAPL1 | rs4367173 | 4 | 7383470 | C | G | 0.17 | 5,367 | -0.15 | 0.02 | 3.44E-11 | 29.61 |
| PSAPL1 | rs4689111 | 4 | 7384238 | G | A | 0.17 | 5,367 | -0.15 | 0.02 | 6.91E-11 | 110.55 |
| PSAPL1 | rs3905844 | 4 | 7385065 | C | A | 0.18 | 5,367 | -0.15 | 0.02 | 5.47E-11 | 33.27 |
| PSAPL1 | rs28682976 | 4 | 7385140 | T | C | 0.37 | 5,367 | -0.12 | 0.02 | 4.15E-11 | 72.06 |
| PSAPL1 | rs252034 | 5 | 52614791 | G | A | 0.41 | 5,367 | 0.10 | 0.02 | 2.10E-08 | 181.16 |
| PSAPL1 | rs6073972 | 20 | 44590298 | C | G | 0.22 | 5,367 | 0.13 | 0.02 | 7.53E-10 | 101.46 |
| RNASE1 | rs10480300 | 7 | 151406005 | C | T | 0.26 | 5,366 | 0.11 | 0.02 | 1.36E-08 | 160.85 |
| RNASE1 | rs17173238 | 7 | 151406220 | A | G | 0.28 | 5,366 | 0.11 | 0.02 | 2.85E-08 | 97.42 |
| RNASE1 | rs10872856 | 14 | 21286675 | C | T | 0.09 | 5,366 | -0.19 | 0.03 | 2.89E-09 | 56.04 |
| RNASE1 | rs56216219 | 14 | 21287223 | A | G | 0.04 | 5,366 | -0.40 | 0.05 | 1.21E-18 | 925.06 |
| RNASE1 | rs72678358 | 14 | 21288839 | A | G | 0.04 | 5,366 | -0.37 | 0.05 | 3.94E-16 | 2527.22 |
| RNASE1 | rs117219627 | 14 | 21425667 | A | G | 0.03 | 5,366 | -0.36 | 0.05 | 5.65E-12 | 75.91 |
| TNXB | rs4711023 | 6 | 22709628 | T | G | 0.11 | 5,363 | -0.17 | 0.03 | 7.19E-09 | 44.67 |
| TNXB | rs56124105 | 6 | 25431771 | G | T | 0.03 | 5,363 | -0.37 | 0.06 | 2.55E-10 | 75.86 |
| TNXB | rs13192826 | 6 | 25496657 | G | A | 0.15 | 5,363 | 0.14 | 0.03 | 3.64E-08 | 2150.01 |
| TNXB | rs114159556 | 6 | 25661984 | T | G | 0.02 | 5,363 | -0.39 | 0.06 | 1.02E-10 | 52.41 |
| TNXB | rs75744298 | 6 | 26239502 | A | G | 0.05 | 5,363 | -0.24 | 0.04 | 1.94E-08 | 64.34 |
| TNXB | rs143257546 | 6 | 28059913 | C | G | 0.02 | 5,363 | -0.44 | 0.07 | 5.64E-11 | 210.95 |
| TNXB | rs11753455 | 6 | 28596252 | A | C | 0.02 | 5,363 | -0.51 | 0.08 | 3.51E-11 | 63.30 |
| TNXB | rs4713186 | 6 | 28909465 | T | C | 0.42 | 5,363 | 0.11 | 0.02 | 8.57E-09 | 123.86 |
| TNXB | rs6456891 | 6 | 29016587 | A | G | 0.41 | 5,363 | 0.11 | 0.02 | 2.91E-08 | 168.88 |
| TNXB | rs114685781 | 6 | 29107198 | A | G | 0.01 | 5,363 | -0.66 | 0.08 | 3.11E-16 | 194.23 |
| TNXB | rs151071749 | 6 | 29251786 | C | T | 0.01 | 5,363 | -0.81 | 0.08 | 1.46E-22 | 10466.57 |
| TNXB | rs17184086 | 6 | 29430213 | G | A | 0.02 | 5,363 | -0.67 | 0.07 | 1.99E-19 | 119.04 |
| TNXB | rs1233417 | 6 | 29511850 | T | C | 0.24 | 5,363 | 0.13 | 0.02 | 1.13E-08 | 166.35 |
| TNXB | rs138131857 | 6 | 29654098 | G | A | 0.02 | 5,363 | -0.96 | 0.07 | 1.42E-44 | 249.26 |
| TNXB | rs9258554 | 6 | 29812559 | T | C | 0.02 | 5,363 | -0.93 | 0.06 | 1.97E-45 | 227.37 |
| TNXB | rs2523938 | 6 | 29926976 | A | G | 0.15 | 5,363 | 0.17 | 0.03 | 2.05E-10 | 351.99 |
| TNXB | rs9261032 | 6 | 29966124 | T | A | 0.03 | 5,363 | -0.60 | 0.05 | 3.08E-29 | 254.73 |
| TNXB | rs115893523 | 6 | 30076828 | A | T | 0.01 | 5,363 | -0.53 | 0.09 | 9.31E-10 | 491.03 |
| TNXB | rs9261487 | 6 | 30109207 | C | A | 0.02 | 5,363 | -0.92 | 0.06 | 4.01E-46 | 107.69 |
| TNXB | rs2257914 | 6 | 30120563 | C | A | 0.11 | 5,363 | 0.20 | 0.03 | 1.11E-11 | 254.04 |
| TNXB | rs928824 | 6 | 30224889 | C | T | 0.06 | 5,363 | -0.45 | 0.04 | 3.52E-28 | 41.09 |
| TNXB | rs2844754 | 6 | 30246212 | C | A | 0.04 | 5,363 | 0.29 | 0.05 | 4.88E-09 | 134.15 |
| TNXB | rs114611882 | 6 | 30320209 | G | A | 0.03 | 5,363 | -0.34 | 0.05 | 9.03E-11 | 66.75 |
| TNXB | rs2524204 | 6 | 30359697 | T | C | 0.13 | 5,363 | -0.20 | 0.03 | 6.48E-13 | 55.32 |
| TNXB | rs143654430 | 6 | 30487304 | T | C | 0.02 | 5,363 | -1.04 | 0.07 | 1.63E-50 | 41.30 |
| TNXB | rs3130666 | 6 | 30740160 | G | A | 0.03 | 5,363 | 0.31 | 0.05 | 4.45E-09 | 118.04 |
| TNXB | rs116763020 | 6 | 30742141 | G | A | 0.02 | 5,363 | -1.15 | 0.07 | 6.94E-56 | 27.12 |
| TNXB | rs116045725 | 6 | 30847797 | G | A | 0.06 | 5,363 | -0.33 | 0.04 | 4.45E-18 | 291.15 |
| TNXB | rs115435966 | 6 | 30998558 | C | A | 0.02 | 5,363 | -1.02 | 0.06 | 4.42E-55 | 245.92 |
| TNXB | rs3909112 | 6 | 31017256 | G | T | 0.02 | 5,363 | -0.41 | 0.06 | 1.42E-11 | 33.16 |
| TNXB | rs147539478 | 6 | 31123434 | A | C | 0.02 | 5,363 | -1.05 | 0.07 | 5.86E-57 | 72.71 |
| TNXB | rs73728292 | 6 | 31170066 | A | G | 0.09 | 5,363 | -0.24 | 0.03 | 3.56E-12 | 30.49 |
| TNXB | rs17408553 | 6 | 31239407 | G | T | 0.31 | 5,363 | 0.14 | 0.02 | 8.40E-12 | 39.78 |
| TNXB | rs115032094 | 6 | 31340001 | A | G | 0.02 | 5,363 | -1.39 | 0.07 | 2.68E-78 | 195.07 |
| TNXB | rs9266720 | 6 | 31349809 | A | G | 0.02 | 5,363 | 0.41 | 0.07 | 3.21E-08 | 49.52 |
| TNXB | rs115793853 | 6 | 31444753 | C | G | 0.01 | 5,363 | -1.55 | 0.09 | 1.36E-66 | 62.77 |
| TNXB | rs114921900 | 6 | 31486065 | G | T | 0.02 | 5,363 | -0.91 | 0.06 | 7.84E-50 | 169.17 |
| TNXB | rs9267502 | 6 | 31553194 | G | A | 0.06 | 5,363 | 0.24 | 0.04 | 3.66E-09 | 128.65 |
| TNXB | rs150043706 | 6 | 31578465 | G | A | 0.02 | 5,363 | -1.45 | 0.07 | 3.09E-83 | 499.85 |
| TNXB | rs2178899 | 6 | 31606756 | A | T | 0.11 | 5,363 | 0.19 | 0.03 | 8.39E-11 | 42.16 |
| TNXB | rs36048548 | 6 | 31634971 | C | T | 0.03 | 5,363 | -0.30 | 0.05 | 5.32E-09 | 48.30 |
| TNXB | rs11575850 | 6 | 31682213 | G | C | 0.02 | 5,363 | -1.51 | 0.07 | 7.02E-89 | 26.39 |
| TNXB | rs2736197 | 6 | 31688388 | G | T | 0.02 | 5,363 | -1.14 | 0.06 | 4.55E-67 | 104.14 |
| TNXB | rs13207465 | 6 | 31768055 | G | T | 0.11 | 5,363 | 0.21 | 0.03 | 1.46E-11 | 35.97 |
| TNXB | rs190263387 | 6 | 31787120 | G | T | 0.02 | 5,363 | -1.49 | 0.07 | 1.44E-94 | 32.40 |
| TNXB | rs11576012 | 6 | 31795619 | C | T | 0.01 | 5,363 | -0.58 | 0.09 | 2.52E-11 | 71.32 |
| TNXB | rs628357 | 6 | 31806598 | G | A | 0.01 | 5,363 | 0.67 | 0.09 | 1.67E-14 | 124.95 |
| TNXB | rs45451301 | 6 | 31935392 | T | C | 0.04 | 5,363 | -1.60 | 0.04 | 8.63E-254 | 38.92 |
| TNXB | rs391165 | 6 | 31947086 | G | A | 0.14 | 5,363 | 0.26 | 0.03 | 1.24E-21 | 45.54 |
| TNXB | rs116298992 | 6 | 32068495 | C | T | 0.04 | 5,363 | -1.67 | 0.05 | 1.71E-266 | 76.75 |
| TNXB | rs439844 | 6 | 32072940 | C | T | 0.11 | 5,363 | 0.23 | 0.03 | 5.45E-15 | 51.77 |
| TNXB | rs151002670 | 6 | 32170837 | C | T | 0.04 | 5,363 | -1.26 | 0.04 | 3.68E-178 | 54.32 |
| CD8A | rs17026864 | 2 | 86303495 | G | A | 0.02 | 5,365 | 0.49 | 0.08 | 5.35E-10 | 233.34 |
| CD8A | rs114534559 | 2 | 86382938 | C | T | 0.04 | 5,365 | 0.29 | 0.05 | 2.29E-09 | 36.58 |
| CD8A | rs141133946 | 2 | 86391633 | G | C | 0.03 | 5,365 | 0.40 | 0.06 | 4.65E-13 | 39.18 |
| CD8A | rs113680499 | 2 | 86565667 | C | T | 0.02 | 5,365 | 0.48 | 0.06 | 1.41E-13 | 33.41 |
| CD8A | rs150649551 | 2 | 86583806 | C | T | 0.03 | 5,365 | 0.41 | 0.05 | 5.19E-15 | 34.04 |
| CD8A | rs61748134 | 2 | 86707313 | G | A | 0.02 | 5,365 | 0.41 | 0.07 | 2.41E-09 | 34.76 |
| CD8A | rs151209719 | 2 | 86750489 | A | G | 0.04 | 5,365 | 0.44 | 0.05 | 6.81E-19 | 30.66 |
| CD8A | rs11895434 | 2 | 86890560 | G | A | 0.13 | 5,365 | 0.48 | 0.03 | 9.75E-66 | 32.82 |
| CD8A | rs13028852 | 2 | 86913513 | C | A | 0.06 | 5,365 | 0.47 | 0.04 | 8.58E-33 | 39.29 |
| CD8A | rs3020726 | 2 | 87016506 | A | G | 0.19 | 5,365 | 0.54 | 0.02 | 1.33E-116 | 77.43 |
| CD8A | rs62146083 | 2 | 87052472 | C | T | 0.06 | 5,365 | -0.42 | 0.04 | 1.61E-24 | 224.39 |
| CD8A | rs138995417 | 2 | 87171633 | C | T | 0.06 | 5,365 | 0.23 | 0.04 | 1.29E-08 | 1451.77 |
| CD8A | rs731132 | 6 | 27885356 | T | G | 0.41 | 5,365 | -0.11 | 0.02 | 3.57E-08 | 126.75 |
| CD8A | rs3734572 | 6 | 28059458 | T | C | 0.04 | 5,365 | 0.30 | 0.05 | 9.00E-10 | 48.24 |
| CD8A | rs56057304 | 6 | 28207991 | A | G | 0.04 | 5,365 | 0.31 | 0.05 | 3.90E-10 | 46.32 |
| CD8A | rs116537337 | 6 | 28339478 | A | G | 0.04 | 5,365 | 0.30 | 0.05 | 1.18E-09 | 62.47 |
| CD8A | rs74874610 | 6 | 28456845 | C | T | 0.04 | 5,365 | 0.30 | 0.05 | 1.18E-09 | 40.39 |
| CD8A | rs114339006 | 6 | 28696794 | G | T | 0.05 | 5,365 | 0.30 | 0.04 | 5.54E-12 | 40.41 |
| CD8A | rs114146785 | 6 | 28797097 | A | G | 0.04 | 5,365 | 0.33 | 0.05 | 2.43E-11 | 32.73 |
| CD8A | rs9468447 | 6 | 28916872 | T | C | 0.04 | 5,365 | 0.34 | 0.05 | 1.11E-11 | 44.26 |
| CD8A | rs112646125 | 6 | 29043953 | G | A | 0.04 | 5,365 | 0.33 | 0.05 | 2.03E-11 | 170.98 |
| CD8A | rs9295795 | 6 | 29145623 | T | C | 0.04 | 5,365 | 0.33 | 0.05 | 2.36E-11 | 28.48 |
| CD8A | rs9461514 | 6 | 29256476 | G | A | 0.04 | 5,365 | 0.34 | 0.05 | 9.46E-12 | 1428.41 |
| CD8A | rs55969931 | 6 | 29406542 | T | G | 0.05 | 5,365 | 0.34 | 0.04 | 4.66E-14 | 73.56 |
| CD8A | rs28893514 | 6 | 29564486 | T | G | 0.05 | 5,365 | 0.33 | 0.04 | 2.04E-15 | 40.25 |
| CD8A | rs115938232 | 6 | 29725013 | A | G | 0.05 | 5,365 | 0.34 | 0.04 | 1.95E-14 | 85.86 |
| CD8A | rs113360274 | 6 | 29850784 | A | G | 0.24 | 5,365 | -0.13 | 0.02 | 1.38E-08 | 39.12 |
| CD8A | rs3128993 | 6 | 29876869 | A | G | 0.13 | 5,365 | 0.22 | 0.03 | 1.17E-14 | 47.94 |
| CD8A | rs356971 | 6 | 29979797 | T | G | 0.13 | 5,365 | 0.20 | 0.03 | 6.22E-12 | 72.00 |
| CD8A | rs2523734 | 6 | 30129676 | A | C | 0.11 | 5,365 | 0.26 | 0.03 | 1.05E-18 | 92.92 |
| CD8A | rs73425423 | 6 | 30230395 | G | C | 0.02 | 5,365 | 0.45 | 0.07 | 9.80E-11 | 106.79 |
| CD8A | rs28780092 | 6 | 30234991 | A | G | 0.06 | 5,365 | 0.36 | 0.04 | 1.09E-17 | 31.33 |
| CD8A | rs28780099 | 6 | 30351417 | A | G | 0.07 | 5,365 | 0.35 | 0.04 | 1.68E-19 | 33.78 |
| CD8A | rs17195173 | 6 | 30414088 | G | A | 0.02 | 5,365 | 0.43 | 0.07 | 2.40E-10 | 44.59 |
| CD8A | rs28780108 | 6 | 30455240 | G | C | 0.06 | 5,365 | 0.41 | 0.04 | 4.41E-25 | 498.89 |
| CD8A | rs73727439 | 6 | 30518874 | C | A | 0.02 | 5,365 | 0.43 | 0.07 | 2.40E-10 | 47.25 |
| CD8A | rs3873304 | 6 | 30586031 | G | A | 0.07 | 5,365 | 0.37 | 0.04 | 6.39E-24 | 181.66 |
| CD8A | rs117626418 | 6 | 30634005 | G | A | 0.02 | 5,365 | 0.43 | 0.07 | 2.40E-10 | 145.80 |
| CD8A | rs28780110 | 6 | 30719578 | G | A | 0.05 | 5,365 | 0.47 | 0.04 | 1.34E-28 | 47.91 |
| CD8A | rs112656743 | 6 | 30739183 | A | C | 0.02 | 5,365 | 0.45 | 0.06 | 3.42E-12 | 56.74 |
| CD8A | rs34331231 | 6 | 30864927 | C | T | 0.06 | 5,365 | 0.51 | 0.04 | 1.70E-35 | 36.90 |
| CD8A | rs55883436 | 6 | 30932429 | A | G | 0.02 | 5,365 | 0.45 | 0.07 | 9.04E-12 | 185.79 |
| CD8A | rs13210132 | 6 | 31001143 | A | G | 0.06 | 5,365 | 0.54 | 0.04 | 2.40E-40 | 1875.94 |
| CD8A | rs13198298 | 6 | 31103589 | C | T | 0.09 | 5,365 | 0.49 | 0.03 | 2.06E-52 | 329.56 |
| CHIT1 | rs861272 | 1 | 201269122 | A | C | 0.22 | 5,368 | 0.14 | 0.02 | 1.67E-09 | 115.37 |
| CHIT1 | rs149550609 | 1 | 201549699 | G | A | 0.02 | 5,368 | -0.44 | 0.06 | 1.76E-12 | 96.42 |
| CHIT1 | rs116201986 | 1 | 201634155 | C | T | 0.03 | 5,368 | -0.39 | 0.06 | 8.70E-11 | 58.92 |
| CHIT1 | rs76577526 | 1 | 201820094 | G | A | 0.04 | 5,368 | -0.28 | 0.05 | 3.37E-08 | 202.60 |
| CHIT1 | rs150506569 | 1 | 202029848 | T | C | 0.02 | 5,368 | -0.38 | 0.06 | 9.99E-10 | 82.22 |
| CHIT1 | rs4486500 | 1 | 202089372 | G | A | 0.14 | 5,368 | 0.18 | 0.03 | 6.19E-11 | 75.69 |
| CHIT1 | rs11586832 | 1 | 202251910 | C | T | 0.02 | 5,368 | 0.45 | 0.07 | 6.86E-10 | 56.44 |
| CHIT1 | rs79811259 | 1 | 202304709 | G | T | 0.03 | 5,368 | 0.35 | 0.06 | 2.33E-09 | 137.36 |
| CHIT1 | rs75136553 | 1 | 202348098 | C | G | 0.01 | 5,368 | -0.63 | 0.08 | 9.58E-14 | 51.17 |
| CHIT1 | rs2696956 | 1 | 202350180 | C | T | 0.32 | 5,368 | -0.12 | 0.02 | 1.18E-09 | 33.97 |
| CHIT1 | rs705740 | 1 | 202459999 | A | G | 0.32 | 5,368 | -0.13 | 0.02 | 5.41E-10 | 1716.98 |
| CHIT1 | rs138168822 | 1 | 202475810 | G | A | 0.03 | 5,368 | 0.36 | 0.06 | 1.56E-09 | 45.68 |
| CHIT1 | rs12402799 | 1 | 202616151 | T | C | 0.11 | 5,368 | -0.17 | 0.03 | 8.72E-09 | 40.26 |
| CHIT1 | rs114323184 | 1 | 202655679 | G | A | 0.05 | 5,368 | 0.29 | 0.04 | 2.66E-11 | 133.08 |
| CHIT1 | rs114475484 | 1 | 202740692 | T | C | 0.05 | 5,368 | 0.28 | 0.04 | 7.64E-11 | 35.73 |
| CHIT1 | rs12074557 | 1 | 202782257 | G | A | 0.11 | 5,368 | 0.18 | 0.03 | 4.86E-09 | 91.28 |
| CHIT1 | rs75055921 | 1 | 202857474 | T | C | 0.02 | 5,368 | -0.53 | 0.08 | 1.65E-12 | 120.03 |
| CHIT1 | rs114170147 | 1 | 202896142 | G | C | 0.09 | 5,368 | 0.22 | 0.03 | 6.86E-12 | 44.60 |
| CHIT1 | rs79483487 | 1 | 203008236 | C | A | 0.02 | 5,368 | 0.53 | 0.06 | 2.14E-17 | 40.60 |
| CHIT1 | rs114629482 | 1 | 203016320 | T | C | 0.21 | 5,368 | 0.22 | 0.02 | 3.98E-21 | 48.27 |
| CHIT1 | rs12161235 | 1 | 203035608 | G | T | 0.08 | 5,368 | -0.23 | 0.03 | 2.91E-11 | 76.96 |
| CHIT1 | rs3898276 | 1 | 203073796 | A | C | 0.07 | 5,368 | 0.25 | 0.04 | 4.35E-12 | 63.66 |
| CHIT1 | rs111768615 | 1 | 203155412 | G | A | 0.03 | 5,368 | -0.73 | 0.06 | 2.66E-35 | 134.53 |
| CHIT1 | rs872129 | 1 | 203169391 | A | G | 0.08 | 5,368 | -0.73 | 0.03 | 7.04E-97 | 153.81 |
| CHIT1 | rs35110680 | 1 | 203170892 | C | T | 0.26 | 5,368 | 0.77 | 0.02 | 2.02E-293 | 373.77 |
| CHIT1 | rs77190654 | 1 | 203177311 | C | T | 0.03 | 5,368 | 0.62 | 0.06 | 2.59E-25 | 33.72 |
| CHIT1 | rs77706048 | 1 | 203189588 | C | T | 0.01 | 5,368 | -0.76 | 0.08 | 1.08E-19 | 153.77 |
| CHIT1 | rs10920585 | 1 | 203190663 | C | A | 0.02 | 5,368 | -0.89 | 0.06 | 5.50E-42 | 570.67 |
| CST5 | rs35488686 | 20 | 23746561 | C | A | 0.32 | 982 | 0.50 | 0.09 | 8.07E-09 | 77.03 |
| CST5 | rs4378871 | 20 | 23746806 | A | G | 0.03 | 982 | 0.37 | 0.06 | 1.00E-11 | 50.91 |
| CST5 | rs35127038 | 20 | 23753051 | C | A | 0.11 | 982 | 0.45 | 0.08 | 2.17E-08 | 362.47 |
| CNTN1 | rs7896061 | 10 | 73414010 | T | C | 0.24 | 5,362 | 0.12 | 0.02 | 1.18E-08 | 158.47 |
| CNTN1 | rs7311094 | 12 | 41019837 | G | A | 0.32 | 5,362 | 0.11 | 0.02 | 3.12E-08 | 125.57 |
| CNTN1 | rs10878921 | 12 | 41023470 | G | A | 0.43 | 5,362 | 0.10 | 0.02 | 4.34E-08 | 111.57 |
| CNTN1 | rs7307329 | 12 | 41025764 | C | T | 0.41 | 5,362 | 0.10 | 0.02 | 4.34E-08 | 183.87 |
| TNFRSF10B | rs59296513 | 8 | 23024780 | G | A | 0.03 | 5,367 | 0.14 | 0.06 | 4.01E-13 | 92.14 |
| NPPB | rs10218813 | 1 | 11825851 | C | G | 0.34 | 5,368 | 0.13 | 0.02 | 5.64E-12 | 54.02 |
| NPPB | rs114941496 | 1 | 11826085 | G | A | 0.06 | 5,368 | 0.24 | 0.04 | 3.08E-10 | 48.23 |
| SERPINA7 | rs1476421 | X | 106000590 | A | G | 0.19 | 5,362 | 0.28 | 0.13 | 3.01E-16 | 85.49 |
| SFRP4 | rs184676007 | 7 | 37808083 | G | A | 0.02 | 5,362 | 0.39 | 0.07 | 4.61E-08 | 89.60 |
| SFRP4 | rs62444777 | 7 | 37934792 | A | G | 0.09 | 5,362 | 0.24 | 0.03 | 1.37E-13 | 59.97 |
| SFRP4 | rs10226308 | 7 | 37938422 | A | G | 0.20 | 5,362 | -0.17 | 0.02 | 3.73E-13 | 53.85 |
| SFRP4 | rs10256195 | 7 | 37938610 | G | A | 0.20 | 5,362 | -0.18 | 0.02 | 1.37E-13 | 79.61 |

**Table S12. Results of Mendelian Randomization analysis.**

| **Forward Mendelian Randomization analysis** | | | | | | | | | | | |
| --- | --- | --- | --- | --- | --- | --- | --- | --- | --- | --- | --- |
| Exposure | Outcome | method | nsnp | b | se | pval | ci_lower | ci_upper | beta_ci | pval (pleiotropy) | P-value threshold |
| NTproBNP (GCST90012082) | Aortic valve stenosis | Wald ratio | 1 | -0.054984943 | 0.067062962 | 0.412272942 | -0.186428348 | 0.076458462 | -0.055 (-0.186 to 0.076) | Not Applicable | 5.00E-08 |
|  |  |  |  |  |  |  |  |  |  |  |  |
|  |  |  |  |  |  |  |  |  |  |  |  |
| CNTN5 (GCST90088299) | Aortic valve stenosis | Inverse variance weighted | 3 | 0.129144138323302 | 0.0608402175077633 | 0.0337809166806384 | 0.00989731200808593 | 0.248390964638518 | 0.129 (0.010 to 0.248) | 0.8776161 | 5.00E-08 |
|  |  | MR Egger | 3 | -0.046720114 | 0.905563953212929 | 0.967184425923827 | -1.821625463 | 1.72818523405964 | -0.047 (-1.822 to 1.728) |  |  |
|  |  | Weighted median | 3 | 0.134694950774899 | 0.0739794436832846 | 0.0686512846021793 | -0.010304759 | 0.279694660394137 | 0.135 (-0.010 to 0.280) |  |  |
| HAVCR1 (GCST90090453) | Aortic valve stenosis | Inverse variance weighted | 17 | -0.003937009 | 0.0191259865805398 | 0.836911158636617 | -0.041423943 | 0.0335499246615887 | -0.004 (-0.041 to 0.034) | 0.9969032 | 5.00E-08 |
|  |  | MR Egger | 17 | -0.004146651 | 0.0564597338950014 | 0.942422927137942 | -0.11480773 | 0.106514427131023 | -0.004 (-0.115 to 0.107) |  |  |
|  |  | Weighted median | 17 | -0.000316294 | 0.0253042860229697 | 0.990027009368835 | -0.049912694 | 0.049280106755878 | -0.000 (-0.050 to 0.049) |  |  |
| BCAN (GCST90088400) | Aortic valve stenosis | Inverse variance weighted | 3 | -0.028741086 | 0.0484688795401501 | 0.553194407268901 | -0.123740089 | 0.0662579183069014 | -0.029 (-0.124 to 0.066) | 0.9070494 | 5.00E-08 |
|  |  | MR Egger | 3 | -0.011097048 | 0.129404229437882 | 0.945539965420125 | -0.264729338 | 0.242535241552978 | -0.011 (-0.265 to 0.243) |  |  |
|  |  | Weighted median | 3 | -0.024009648 | 0.0540906896240695 | 0.657131050996143 | -0.130027399 | 0.0820081039752792 | -0.024 (-0.130 to 0.082) |  |  |
| IGFBP7 (GCST90088312) | Aortic valve stenosis | Inverse variance weighted | 5 | -0.089039217 | 0.0908259793104424 | 0.326924418773396 | -0.267058137 | 0.0889797021402117 | -0.089 (-0.267 to 0.089) | 0.1141312 | 5.00E-08 |
|  |  | MR Egger | 5 | 0.411607675495508 | 0.235622405546885 | 0.178985293548346 | -0.050212239 | 0.873427590367403 | 0.412 (-0.050 to 0.873) |  |  |
|  |  | Weighted median | 5 | 0.02630741311376 | 0.0573579299004229 | 0.646483038537166 | -0.086114129 | 0.138728955718589 | 0.026 (-0.086 to 0.139) |  |  |
| GDF15 (GCST90088672) | Aortic valve stenosis | Inverse variance weighted | 3 | 0.0432914235091558 | 0.0846335885676093 | 0.608989880180349 | -0.12259041 | 0.20917325710167 | 0.043 (-0.123 to 0.209) | 0.8494365 | 5.00E-08 |
|  |  | MR Egger | 3 | -0.041712701 | 0.362704389778645 | 0.927105991322867 | -0.752613305 | 0.669187903377199 | -0.042 (-0.753 to 0.669) |  |  |
|  |  | Weighted median | 3 | 0.06786153700388 | 0.0969620106882779 | 0.484003762477714 | -0.122184004 | 0.257907077952905 | 0.068 (-0.122 to 0.258) |  |  |
| CD200 (GCST90088934) | Aortic valve stenosis | Inverse variance weighted | 9 | -0.072102396 | 0.048293443 | 0.135435642 | -0.166757544 | 0.022552753 | -0.072 (-0.167 to 0.023) | 0.919272 | 5.00E-08 |
|  |  | MR Egger | 9 | -0.059572918 | 0.129934866 | 0.66050038 | -0.314245256 | 0.195099419 | -0.060 (-0.314 to 0.195) |  |  |
|  |  | Weighted median | 9 | -0.11119343 | 0.04197842 | 0.008077248 | -0.193471133 | -0.028915726 | -0.111 (-0.193 to -0.029) |  |  |
| FGL1 (GCST90089073) | Aortic valve stenosis | Inverse variance weighted | 6 | 0.017462362 | 0.02202846 | 0.427941991 | -0.025713419 | 0.060638143 | 0.017 (-0.026 to 0.061) | 0.9199413 | 5.00E-08 |
|  |  | MR Egger | 6 | 0.023099582 | 0.057104488 | 0.706531093 | -0.088825215 | 0.13502438 | 0.023 (-0.089 to 0.135) |  |  |
|  |  | Weighted median | 6 | 0.012996413 | 0.023868369 | 0.586094955 | -0.033785589 | 0.059778416 | 0.013 (-0.034 to 0.060) |  |  |
| FLT4 (GCST90087934) | Aortic valve stenosis | Inverse variance weighted | 24 | -0.012498522 | 0.013957321 | 0.370529807 | -0.039854872 | 0.014857828 | -0.012 (-0.040 to 0.015) | 0.2264522 | 5.00E-08 |
|  |  | MR Egger | 24 | -0.042792541 | 0.028061788 | 0.14152207 | -0.097793646 | 0.012208563 | -0.043 (-0.098 to 0.012) |  |  |
|  |  | Weighted median | 24 | 0.012756979 | 0.020570886 | 0.535160838 | -0.027561958 | 0.053075915 | 0.013 (-0.028 to 0.053) |  |  |
| MLN (GCST90089107) | Aortic valve stenosis | Inverse variance weighted | 22 | 0.044596381 | 0.019050804 | 0.019236363 | 0.007256805 | 0.081935958 | 0.045 (0.007 to 0.082) | 0.1896899 | 5.00E-08 |
|  |  | MR Egger | 22 | 0.095266396 | 0.041734825 | 0.03352945 | 0.013466139 | 0.177066652 | 0.095 (0.013 to 0.177) |  |  |
|  |  | Weighted median | 22 | 0.072206339 | 0.025316514 | 0.004342545 | 0.022585971 | 0.121826707 | 0.072 (0.023 to 0.122) |  |  |
| PSAPL1 (GCST90090315) | Aortic valve stenosis | Inverse variance weighted | 12 | 0.004489236 | 0.031663174 | 0.887252995 | -0.057570584 | 0.066549057 | 0.004 (-0.058 to 0.067) | 0.5943863 | 5.00E-08 |
|  |  | MR Egger | 12 | -0.022591291 | 0.059115975 | 0.710344402 | -0.138458602 | 0.09327602 | -0.023 (-0.138 to 0.093) |  |  |
|  |  | Weighted median | 12 | -0.004091441 | 0.032876326 | 0.90095942 | -0.06852904 | 0.060346158 | -0.004 (-0.069 to 0.060) |  |  |
| RNASE1 (GCST90089740) | Aortic valve stenosis | Inverse variance weighted | 6 | 0.009873972 | 0.051764929 | 0.848724305 | -0.091585288 | 0.111333233 | 0.010 (-0.092 to 0.111) | 0.7968551 | 5.00E-08 |
|  |  | MR Egger | 6 | 0.042914627 | 0.13308236 | 0.763250166 | -0.217926799 | 0.303756054 | 0.043 (-0.218 to 0.304) |  |  |
|  |  | Weighted median | 6 | 0.059882446 | 0.054695433 | 0.273589159 | -0.047320603 | 0.167085495 | 0.060 (-0.047 to 0.167) |  |  |
| TNXB (GCST90089154) | Aortic valve stenosis | Inverse variance weighted | 52 | 0.021566151 | 0.009814433 | 0.027992508 | 0.002329862 | 0.040802441 | 0.022 (0.002 to 0.041) | 0.4641871 | 5.00E-08 |
|  |  | MR Egger | 52 | 0.028629412 | 0.01374352 | 0.042373911 | 0.001692114 | 0.05556671 | 0.029 (0.002 to 0.056) |  |  |
|  |  | Weighted median | 52 | 0.016809009 | 0.012178778 | 0.167528638 | -0.007061397 | 0.040679414 | 0.017 (-0.007 to 0.041) |  |  |
| CD8A (GCST90089255) | Mitral valve regurgitation | Inverse variance weighted | 44 | 0.10745122 | 0.042873849 | 0.012203029 | 0.023418476 | 0.191483964 | 0.107 (0.023 to 0.191) | 0.1964344 | 5.00E-08 |
|  |  | MR Egger | 44 | -0.022325944 | 0.10776367 | 0.836874367 | -0.233542736 | 0.188890849 | -0.022 (-0.234 to 0.189) |  |  |
|  |  | Weighted median | 44 | 0.157010866 | 0.062770537 | 0.012372204 | 0.033980614 | 0.280041118 | 0.157 (0.034 to 0.280) |  |  |
| CHIT1 (GCST90086282) | Mitral valve regurgitation | Inverse variance weighted | 28 | 0.040487817 | 0.050665576 | 0.424221494 | -0.058816711 | 0.139792346 | 0.040 (-0.059 to 0.140) | 0.1136022 | 5.00E-08 |
|  |  | MR Egger | 28 | -0.071923629 | 0.085208103 | 0.406316808 | -0.238931511 | 0.095084254 | -0.072 (-0.239 to 0.095) |  |  |
|  |  | Weighted median | 28 | 0.007891335 | 0.071067081 | 0.911584116 | -0.131400145 | 0.147182814 | 0.008 (-0.131 to 0.147) |  |  |
| CST5 (GCST90000456) | Mitral valve regurgitation | Inverse variance weighted | 3 | 0.060211417 | 0.070724989 | 0.394577329 | -0.078409561 | 0.198832395 | 0.060 (-0.078 to 0.199) | 0.8497902 | 5.00E-08 |
|  |  | MR Egger | 3 | 0.106496928 | 0.205093803 | 0.695099674 | -0.295486926 | 0.508480781 | 0.106 (-0.295 to 0.508) |  |  |
|  |  | Weighted median | 3 | 0.057994849 | 0.07558121 | 0.44289226 | -0.090144322 | 0.20613402 | 0.058 (-0.090 to 0.206) |  |  |
| CNTN1 (GCST90088159) | Mitral valve regurgitation | Inverse variance weighted | 4 | 0.032429467 | 0.186566567 | 0.862004963 | -0.333241004 | 0.398099938 | 0.032 (-0.333 to 0.398) | 0.5191556 | 5.00E-08 |
|  |  | MR Egger | 4 | 0.842019371 | 1.062928252 | 0.511298042 | -1.241320002 | 2.925358744 | 0.842 (-1.241 to 2.925) |  |  |
|  |  | Weighted median | 4 | 0.114668306 | 0.209189058 | 0.583584564 | -0.295342247 | 0.52467886 | 0.115 (-0.295 to 0.525) |  |  |
| TNFRSF10B (GCST90089783) | Mitral valve regurgitation | Wald ratio | 1 | 0.170830241 | 0.380704048 | 0.653632289 | -0.575349693 | 0.917010175 | 0.171 (-0.575 to 0.917) | Not Applicable | 5.00E-08 |
|  |  |  |  |  |  |  |  |  |  |  |  |
|  |  |  |  |  |  |  |  |  |  |  |  |
| NPPB (GCST90089781) | Mitral valve regurgitation | Inverse variance weighted | 2 | -0.226636023 | 0.433413011 | 0.601036845 | -1.076125525 | 0.622853479 | -0.227 (-1.076 to 0.623) | Not Applicable | 5.00E-08 |
|  |  |  |  |  |  |  |  |  |  |  |  |
|  |  |  |  |  |  |  |  |  |  |  |  |
| SERPINA7 (GCST90088028) | Mitral valve regurgitation | Wald ratio | 1 | 0.43268382 | 0.485868847 | 0.373178014 | -0.519619121 | 1.38498676 | 0.433 (-0.520 to 1.385) | Not Applicable | 5.00E-08 |
|  |  |  |  |  |  |  |  |  |  |  |  |
|  |  |  |  |  |  |  |  |  |  |  |  |
| SFRP4 (GCST90090607) | Mitral valve regurgitation | Inverse variance weighted | 4 | 0.093943225 | 0.361014209 | 0.794693848 | -0.613644624 | 0.801531074 | 0.094 (-0.614 to 0.802) | 0.2635357 | 5.00E-08 |
|  |  | MR Egger | 4 | -1.487571488 | 1.069870798 | 0.298916887 | -3.584518252 | 0.609375276 | -1.488 (-3.585 to 0.609) |  |  |
|  |  | Weighted median | 4 | -0.229061459 | 0.224588661 | 0.307768533 | -0.669255236 | 0.211132317 | -0.229 (-0.669 to 0.211) |  |  |
| NTproBNP (GCST90012082) | Mitral valve regurgitation | Wald ratio | 1 | -0.290136664 | 0.240662414 | 0.227981285 | -0.761834995 | 0.181561668 | -0.290 (-0.762 to 0.182) | Not Applicable | 5.00E-08 |
|  |  |  |  |  |  |  |  |  |  |  |  |
|  |  |  |  |  |  |  |  |  |  |  |  |
| HAVCR1 (GCST90090453) | Mitral valve regurgitation | Inverse variance weighted | 17 | -0.053248558 | 0.0676490758784826 | 0.431206279133366 | -0.185840747 | 0.079343630552988 | -0.053 (-0.186 to 0.079) | 0.7555125 | 5.00E-08 |
|  |  | MR Egger | 17 | 0.00663944845178158 | 0.200733453301529 | 0.974050144578378 | -0.38679812 | 0.400077016922779 | 0.007 (-0.387 to 0.400) |  |  |
|  |  | Weighted median | 17 | 0.015572086809686 | 0.0884942520990079 | 0.860319701589979 | -0.157876647 | 0.189020820923741 | 0.016 (-0.158 to 0.189) |  |  |
| IGFBP7 (GCST90088312) | Mitral valve regurgitation | Inverse variance weighted | 5 | 0.153551488126687 | 0.153947381717827 | 0.318556619767207 | -0.14818538 | 0.455288356293629 | 0.154 (-0.148 to 0.455) | 0.2318642 | 5.00E-08 |
|  |  | MR Egger | 5 | -0.655238039 | 0.562584414615915 | 0.328339535570798 | -1.757903492 | 0.447427413418058 | -0.655 (-1.758 to 0.447) |  |  |
|  |  | Weighted median | 5 | 0.166300901605581 | 0.181657174974501 | 0.359947233643865 | -0.189747161 | 0.522348964555603 | 0.166 (-0.190 to 0.522) |  |  |
| GDF15 (GCST90088672) | Mitral valve regurgitation | Inverse variance weighted | 3 | -0.275185583 | 0.250120687437597 | 0.271240109044387 | -0.765422131 | 0.215050964112158 | -0.275 (-0.765 to 0.215) | 0.6868893 | 5.00E-08 |
|  |  | MR Egger | 3 | -0.834727798 | 1.07395101401739 | 0.579376483662407 | -2.939671786 | 1.27021618916912 | -0.835 (-2.940 to 1.270) |  |  |
|  |  | Weighted median | 3 | -0.270677749 | 0.298014536256015 | 0.363735435375217 | -0.85478624 | 0.313430741825972 | -0.271 (-0.855 to 0.313) |  |  |
| **Reverse Mendelian Randomization analysis** | | | | | | | | | | | |
| Exposure | Outcome | method | nsnp | b | se | pval | ci_lower | ci_upper | beta_ci | pval (pleiotropy) | P-value threshold |
| Aortic valve stenosis | NTproBNP (GCST90012082) | Wald ratio | 1 | -18.18679712 | 22.18171768 | 0.412272942 | -61.66296377 | 25.28936953 | -18.187 (-61.663 to 25.289) | Not Applicable | 5.00E-08 |
|  |  |  |  |  |  |  |  |  |  |  |  |
|  |  |  |  |  |  |  |  |  |  |  |  |
| Aortic valve stenosis | CNTN5 (GCST90088299) | Inverse variance weighted | 3 | 7.00333852740681 | 1.66090071455463 | 2.48023542392002e-05 | 3.74797312687975 | 10.2587039279339 | 7.003 (3.748 to 10.259) | 0.1427965 | 5.00E-08 |
|  |  | MR Egger | 3 | -0.111902612 | 1.70577940675495 | 0.958296200551562 | -3.455230249 | 3.23142502555114 | -0.112 (-3.455 to 3.231) |  |  |
|  |  | Weighted median | 3 | 6.07182160125106 | 56.99586077 | 0.915161122739361 | -105.6400655 | 117.783708707405 | 6.072 (-105.640 to 117.784) |  |  |
| Aortic valve stenosis | HAVCR1 (GCST90090453) | Inverse variance weighted | 17 | -1.07231246 | 4.21352419622735 | 0.79911470269941 | -9.330819885 | 7.18619496437464 | -1.072 (-9.331 to 7.186) | 0.3444911 | 5.00E-08 |
|  |  | MR Egger | 17 | 3.11356216936082 | 6.01631954888398 | 0.612341155995474 | -8.678424146 | 14.9055484851734 | 3.114 (-8.678 to 14.906) |  |  |
|  |  | Weighted median | 17 | 5.75418100724339 | 12.8072516300075 | 0.653221855479937 | -19.34803219 | 30.8563942 | 5.754 (-19.348 to 30.856) |  |  |
| Aortic valve stenosis | BCAN (GCST90088400) | Inverse variance weighted | 3 | -18.94875352 | 13.3604960170568 | 0.156112763589843 | -45.13532572 | 7.23781866911091 | -18.949 (-45.135 to 7.238) | 0.3116726 | 5.00E-08 |
|  |  | MR Egger | 3 | -0.805808163 | 13.1302502268299 | 0.960979355338273 | -26.54109861 | 24.9294822812041 | -0.806 (-26.541 to 24.929) |  |  |
|  |  | Weighted median | 3 | -14.77266871 | 212.503201484742 | 0.944577788015497 | -431.2789436 | 401.733606200576 | -14.773 (-431.279 to 401.734) |  |  |
| Aortic valve stenosis | IGFBP7 (GCST90088312) | Inverse variance weighted | 5 | -2.284620314 | 1.97934535447358 | 0.248405777487987 | -6.164137209 | 1.59489658093718 | -2.285 (-6.164 to 1.595) | 0.01844642 | 5.00E-08 |
|  |  | MR Egger | 5 | -7.392948825 | 1.34970056523174 | 0.0119654073581447 | -10.03836193 | -4.747535717 | -7.393 (-10.038 to -4.748) |  |  |
|  |  | Weighted median | 5 | -2.592275243 | 2.93658640537391 | 0.377370699391021 | -8.347984598 | 3.16343411151975 | -2.592 (-8.348 to 3.163) |  |  |
| Aortic valve stenosis | GDF15 (GCST90088672) | Inverse variance weighted | 3 | 6.47456216782145 | 7.21139372759575 | 0.369279401806907 | -7.659769538 | 20.6088938739091 | 6.475 (-7.660 to 20.609) | 0.2952101 | 5.00E-08 |
|  |  | MR Egger | 3 | -201.8736418 | 104.291437253448 | 0.303574039290051 | -406.2848588 | 2.537575254 | -201.874 (-406.285 to 2.538) |  |  |
|  |  | Weighted median | 3 | 7.31800545753789 | 115.431957100329 | 0.949450612896691 | -218.9286305 | 233.564641374183 | 7.318 (-218.929 to 233.565) |  |  |
| Aortic valve stenosis | CD200 (GCST90088934) | Inverse variance weighted | 9 | -1.999761855 | 2.157607513 | 0.354008432 | -6.228672579 | 2.22914887 | -2.000 (-6.229 to 2.229) | 0.223149 | 5.00E-08 |
|  |  | MR Egger | 9 | 1.505877514 | 3.334253596 | 0.665190331 | -5.029259534 | 8.041014561 | 1.506 (-5.029 to 8.041) |  |  |
|  |  | Weighted median | 9 | -0.808022121 | 2.375759039 | 0.733772831 | -5.464509838 | 3.848465597 | -0.808 (-5.465 to 3.848) |  |  |
| Aortic valve stenosis | FGL1 (GCST90089073) | Inverse variance weighted | 6 | 6.38430502 | 6.716859214 | 0.341863521 | -6.78073904 | 19.54934908 | 6.384 (-6.781 to 19.549) | 0.1150335 | 5.00E-08 |
|  |  | MR Egger | 6 | 0.209686363 | 6.126646429 | 0.974337281 | -11.79854064 | 12.21791336 | 0.210 (-11.799 to 12.218) |  |  |
|  |  | Weighted median | 6 | 4.103612102 | 55.9872246 | 0.941570966 | -105.6313481 | 113.8385723 | 4.104 (-105.631 to 113.839) |  |  |
| Aortic valve stenosis | FLT4 (GCST90087934) | Inverse variance weighted | 24 | -1.741877426 | 3.333332902 | 0.601278189 | -8.275209915 | 4.791455063 | -1.742 (-8.275 to 4.791) | 0.02533421 | 5.00E-08 |
|  |  | MR Egger | 24 | -8.72913165 | 4.205704442 | 0.049830983 | -16.97231236 | -0.485950943 | -8.729 (-16.972 to -0.486) |  |  |
|  |  | Weighted median | 24 | -5.191878248 | 3.270961922 | 0.112452994 | -11.60296362 | 1.21920712 | -5.192 (-11.603 to 1.219) |  |  |
| Aortic valve stenosis | MLN (GCST90089107) | Inverse variance weighted | 22 | 4.795299131 | 2.028834754 | 0.018099658 | 0.818783012 | 8.771815249 | 4.795 (0.819 to 8.772) | 0.8194534 | 5.00E-08 |
|  |  | MR Egger | 22 | 5.455242485 | 3.528903563 | 0.137812324 | -1.461408498 | 12.37189347 | 5.455 (-1.461 to 12.372) |  |  |
|  |  | Weighted median | 22 | -1.29122178 | 4.147052686 | 0.755527792 | -9.419445045 | 6.837001484 | -1.291 (-9.419 to 6.837) |  |  |
| Aortic valve stenosis | PSAPL1 (GCST90090315) | Inverse variance weighted | 12 | 0.207852267 | 2.949671437 | 0.943822567 | -5.57350375 | 5.989208284 | 0.208 (-5.574 to 5.989) | 0.7546109 | 5.00E-08 |
|  |  | MR Egger | 12 | 1.192927893 | 4.344382786 | 0.78921985 | -7.322062368 | 9.707918154 | 1.193 (-7.322 to 9.708) |  |  |
|  |  | Weighted median | 12 | -0.316210858 | 4.854577883 | 0.948065213 | -9.831183509 | 9.198761793 | -0.316 (-9.831 to 9.199) |  |  |
| Aortic valve stenosis | RNASE1 (GCST90089740) | Inverse variance weighted | 6 | 0.638242829 | 3.573680884 | 0.858255437 | -6.366171703 | 7.642657361 | 0.638 (-6.366 to 7.643) | 0.8709388 | 5.00E-08 |
|  |  | MR Egger | 6 | -0.008269034 | 5.457646307 | 0.998863654 | -10.7052558 | 10.68871773 | -0.008 (-10.705 to 10.689) |  |  |
|  |  | Weighted median | 6 | -0.564534367 | 9.66037539 | 0.953399636 | -19.49887013 | 18.3698014 | -0.565 (-19.499 to 18.370) |  |  |
| Aortic valve stenosis | TNXB (GCST90089154) | Inverse variance weighted | 52 | 4.699538386 | 1.734410079 | 0.006736664 | 1.300094631 | 8.098982142 | 4.700 (1.300 to 8.099) | 0.9342637 | 5.00E-08 |
|  |  | MR Egger | 52 | 4.561605986 | 2.415869246 | 0.064810322 | -0.173497737 | 9.296709708 | 4.562 (-0.173 to 9.297) |  |  |
|  |  | Weighted median | 52 | 3.225745755 | 0.79933691 | 5.44799E-05 | 1.659045412 | 4.792446097 | 3.226 (1.659 to 4.792) |  |  |
| Mitral valve regurgitation | CD8A (GCST90089255) | Inverse variance weighted | 44 | 1.068035288 | 0.529655973 | 0.04375087 | 0.029909581 | 2.106160995 | 1.068 (0.030 to 2.106) | 0.03821056 | 5.00E-08 |
|  |  | MR Egger | 44 | -0.67933393 | 0.962138322 | 0.484044999 | -2.565125042 | 1.206457181 | -0.679 (-2.565 to 1.206) |  |  |
|  |  | Weighted median | 44 | 1.395604441 | 0.967609394 | 0.14921152 | -0.500909971 | 3.292118852 | 1.396 (-0.501 to 3.292) |  |  |
| Mitral valve regurgitation | CHIT1 (GCST90086282) | Inverse variance weighted | 28 | 0.381774608 | 0.758900748 | 0.614920238 | -1.105670858 | 1.869220073 | 0.382 (-1.106 to 1.869) | 0.0646304 | 5.00E-08 |
|  |  | MR Egger | 28 | -0.948496152 | 0.999205339 | 0.351233902 | -2.906938616 | 1.009946312 | -0.948 (-2.907 to 1.010) |  |  |
|  |  | Weighted median | 28 | 1.064798661 | 0.451573039 | 0.018374851 | 0.179715504 | 1.949881818 | 1.065 (0.180 to 1.950) |  |  |
| Mitral valve regurgitation | CST5 (GCST90000456) | Inverse variance weighted | 3 | 15.00515892 | 3.071570627 | 1.03E-06 | 8.984880497 | 21.02543735 | 15.005 (8.985 to 21.025) | 0.1563663 | 5.00E-08 |
|  |  | MR Egger | 3 | 9.160342278 | 1.810716843 | 0.12423847 | 5.611337265 | 12.70934729 | 9.160 (5.611 to 12.709) |  |  |
|  |  | Weighted median | 3 | 13.82106841 | 1507.584465 | 0.992685343 | -2941.044483 | 2968.68662 | 13.821 (-2941.044 to 2968.687) |  |  |
| Mitral valve regurgitation | CNTN1 (GCST90088159) | Inverse variance weighted | 4 | 0.370420019 | 1.720406243 | 0.829525928 | -3.001576218 | 3.742416256 | 0.370 (-3.002 to 3.742) | 0.2046963 | 5.00E-08 |
|  |  | MR Egger | 4 | -3.498626418 | 2.44545658 | 0.288816308 | -8.291721315 | 1.294468478 | -3.499 (-8.292 to 1.294) |  |  |
|  |  | Weighted median | 4 | -0.882992823 | 451.0437712 | 0.99843801 | -884.9287843 | 883.1627987 | -0.883 (-884.929 to 883.163) |  |  |
| Mitral valve regurgitation | TNFRSF10B (GCST90089783) | Wald ratio | 1 | 5.853764491 | 13.04541762 | 0.653632289 | -19.71525404 | 31.42278302 | 5.854 (-19.715 to 31.423) | Not Applicable | 5.00E-08 |
|  |  |  |  |  |  |  |  |  |  |  |  |
|  |  |  |  |  |  |  |  |  |  |  |  |
| Mitral valve regurgitation | NPPB (GCST90089781) | Inverse variance weighted | 2 | -0.226636023 | 0.433413011 | 0.601036845 | -1.076125525 | 0.622853479 | -0.227 (-1.076 to 0.623) | Not Applicable | 5.00E-08 |
|  |  |  |  |  |  |  |  |  |  |  |  |
|  |  |  |  |  |  |  |  |  |  |  |  |
| Mitral valve regurgitation | SERPINA7 (GCST90088028) | Wald ratio | 1 | 2.311156448 | 2.595241302 | 0.373178014 | -2.775516503 | 7.3978294 | 2.311 (-2.776 to 7.398) | Not Applicable | 5.00E-08 |
|  |  |  |  |  |  |  |  |  |  |  |  |
|  |  |  |  |  |  |  |  |  |  |  |  |
| Mitral valve regurgitation | SFRP4 (GCST90090607) | Inverse variance weighted | 4 | 0.243504043 | 0.899145922 | 0.786531748 | -1.518821965 | 2.005830051 | 0.244 (-1.519 to 2.006) | 0.2771078 | 5.00E-08 |
|  |  | MR Egger | 4 | 3.120447415 | 2.088024967 | 0.273663911 | -0.972081521 | 7.212976351 | 3.120 (-0.972 to 7.213) |  |  |
|  |  | Weighted median | 4 | 0.955575236 | 11.95889733 | 0.936312843 | -22.48386353 | 24.395014 | 0.956 (-22.484 to 24.395) |  |  |
| Mitral valve regurgitation | NTproBNP (GCST90012082) | Wald ratio | 1 | -3.446651615 | 2.858926849 | 0.227981285 | -9.050148239 | 2.156845009 | -3.447 (-9.050 to 2.157) | Not Applicable | 5.00E-08 |
|  |  |  |  |  |  |  |  |  |  |  |  |
|  |  |  |  |  |  |  |  |  |  |  |  |
| Mitral valve regurgitation | HAVCR1 (GCST90090453) | Inverse variance weighted | 17 | -0.698704061 | 0.901614025039118 | 0.438370337475457 | -2.46586755 | 1.06845942781196 | -0.699 (-2.466 to 1.068) | 0.03751945 | 5.00E-08 |
|  |  | MR Egger | 17 | -2.537223842 | 1.13704788927835 | 0.0413361457948023 | -4.765837705 | -0.30860998 | -2.537 (-4.766 to -0.309) |  |  |
|  |  | Weighted median | 17 | -0.498046037 | 3.94206101139269 | 0.899461579866958 | -8.22448562 | 7.22839354501356 | -0.498 (-8.224 to 7.228) |  |  |
| Mitral valve regurgitation | CST5 (GCST90000456) | Inverse variance weighted | 3 | 15.0051589248368 | 3.07157062658335 | 1.03337218913337e-06 | 8.98488049673345 | 21.0254373529402 | 15.005 (8.985 to 21.025) | 0.1563663 | 5.00E-08 |
|  |  | MR Egger | 3 | 9.16034227776592 | 1.81071684312137 | 0.124238470186847 | 5.61133726524803 | 12.7093472902838 | 9.160 (5.611 to 12.709) |  |  |
|  |  | Weighted median | 3 | 13.8210684059512 | 1507.584465 | 0.992685343421878 | -2941.044483 | 2968.68661998313 | 13.821 (-2941.044 to 2968.687) |  |  |
| Mitral valve regurgitation | IGFBP7 (GCST90088312) | Inverse variance weighted | 5 | 1.78142050746667 | 1.34563084860923 | 0.185551111853335 | -0.856015956 | 4.41885697074075 | 1.781 (-0.856 to 4.419) | 0.4267939 | 5.00E-08 |
|  |  | MR Egger | 5 | 3.99902415780892 | 2.78116916645851 | 0.246032261069357 | -1.452067408 | 9.45011572406761 | 3.999 (-1.452 to 9.450) |  |  |
|  |  | Weighted median | 5 | 1.63682149467595 | 34.3806886333898 | 0.962028061120163 | -65.74932823 | 69.0229712161199 | 1.637 (-65.749 to 69.023) |  |  |
| Mitral valve regurgitation | GDF15 (GCST90088672) | Inverse variance weighted | 3 | -2.362780637 | 1.229384365 | 0.054615595640226 | -4.772373992 | 0.0468127177307616 | -2.363 (-4.772 to 0.047) | 0.1593588 | 5.00E-08 |
|  |  | MR Egger | 3 | -0.744756011 | 0.597187854786797 | 0.430273645683857 | -1.915244206 | 0.425732184810484 | -0.745 (-1.915 to 0.426) |  |  |
|  |  | Weighted median | 3 | -1.958099075 | 34.0665624029686 | 0.954163934838091 | -68.72856139 | 64.8123632344565 | -1.958 (-68.729 to 64.812) |  |  |
| Reference for the GWAS dataset of aortic valve stenosis: Yu Chen H, Dina C, Small AM, et al. Dyslipidemia, inflammation, calcification, and adiposity in aortic stenosis: a genome-wide study. Eur Heart J. 2023;44(21):1927-1939. doi:10.1093/eurheartj/ehad142 | | | | | | | | | | | |

**Table S13. Bayesian colocalization for causal plasma proteins and diseases.**

| **Outcome** | **Exposure** | **nsnps** | **PP.H0** | **PP.H1** | **PP.H2** | **PP.H3** | **PP.H4** |
| --- | --- | --- | --- | --- | --- | --- | --- |
| Aortic valve stenosis | CNTN5 | 1867 | 0 | 0 | 0.03 | 12.41 | 87.56 |
| Aortic valve stenosis | IGFBP7 | 3543 | 0 | 0 | 0 | 2.09 | 73.03 |
| Mitral valve regurgitation | CD8A | 1867 | 0 | 0 | 0.01 | 17.74 | 82.26 |

**Table S14. Rationale for selection of risk factors.**

| Strength of associations seen in observational and mendelian randomization studies between clinical risk factors and degenerative valvular heart disease | | | | |
| --- | --- | --- | --- | --- |
| **Clinical Risk factor** | | **Degenerative Valvular Heart Disease^1^** | | |
|  |  | **AVS** | **AVR** | **MVR** |
| **Social factor** | Age | +++ | +++ | +++ |
|  | Sex | ++ | ++/- | ++/- |
|  | Ethnicity | ++ | ++ | ++ |
|  | Social-economic class | ++ | ++ | ++ |
|  | Educational attainment | ++ | + | + |
| **Health behavior** | Ever smoked | ++ | + | + |
|  | Alcohol frequency | +/- | + | + |
|  | Diet pattern | +/ none | + | + |
|  | Sleep pattern | + | + | + |
|  | Sedentary pattern | + | + | + |
|  | Physical activity | +/ none | + | + |
| **Cardiometabolic and inflammatory risk factor** | Blood Pressure | +++ | +++ | +++ |
|  | Bilirubin | + | + | NA |
|  | HDL cholesterol | +++ | + | NA |
|  | LDL-C level | +++ | none | none |
|  | Lipoprotein(a) | +++ | NA | NA |
|  | Apolipoprotein A, Apolipoprotein B level | ++ | NA | NA |
|  | Triglycerides | +++ | none | +/ none |
|  | BMI | +++ | NA | + |
|  | HbA1c | + | - | - |
|  | C-reactive protein | ++ / none | NA | +/- |
| **Kidney function and mineral metabolism** | Creatinine | +++ | + | + |
|  | Urate, urea level | NA | NA | - |
|  | Calcium, phosphorous level | ++/-/none | + | + |
| **Medications and supplements** | Lipid-lowering medication | 0 | NA | NA |
|  | Antihypertensive medication | - / none | NA | NA |
|  | Antidiabetic medication^3^ | NA | NA | NA |
|  | Antiplatelet medication^3^ | NA | NA | NA |
|  | Oral anticoagulation medication | ++ | NA | NA |
|  | Antiresorptive medication | - / none | NA | NA |
|  | Vitamins K, Vitamins D supplement | -/ none | NA | NA |
|  | Calcium supplement | + | NA | NA |
| 1. The strength of associations observed in the studies regarding risk factors and degenerative valvular heart disease is categorized as follows: If there is a significant positive association in ≥3 studies, it is represented as ++. In cases where 1-2 studies show a significant positive association, it is denoted as +. For a causative positive correlation, it is marked as +++. Insufficient data available is indicated as NA, a weak negative association is denoted as -, and no association seen is represented as none. Some research outcomes include valve calcification (e.g., mitral annular calcification, aortic valve calcification), early stages of diseases (such as mitral valve prolapse), or undifferentiated etiologies of aortic valve stenosis, aortic valve regurgitation, and mitral valve regurgitation. We categorize the evidence level for these studies as +. | | | | |
| 2. Although there are currently no randomized controlled trials (RCTs) confirming a significant impact on preventing or delaying the progression of valvular heart diseases, antidiabetic medication, and antiplatelet medication are still considered due to the potential influence of corresponding causal risk factors on the outcomes. | | | | |
| Reference |  |  |  |  |
| 1. Li Z, Cheng S, Guo B, Ding L, Liang Y, Shen Y*, et al.* Wearable device-measured moderate to vigorous physical activity and risk of degenerative aortic valve stenosis. *Eur Heart J*. 2025;**46**:649-664. doi:10.1093/eurheartj/ehae406 | | | | |
| 2. Li W, Xiong S, Yin S, Deng W, Zhao Y, Li Z*, et al.* Prevalence and Risk Factors of Mitral, Tricuspid, and Aortic Regurgitation: A Population-Based Study from Rural Northeast China. *Am J Cardiol* 2023;**209**:156-162. doi:10.1016/j.amjcard.2023.09.107 | | | | |
| 3. Nazarzadeh M, Pinho-Gomes AC, Bidel Z, Dehghan A, Canoy D, Hassaine A*, et al.* Plasma lipids and risk of aortic valve stenosis: a Mendelian randomization study. *Eur Heart J* 2020;**41**:3913-3920. doi: 10.1093/eurheartj/ehaa070 | | | | |
| 4. Mathieu P, Arsenault BJ, Boulanger MC, Bossé Y, Koschinsky ML. Pathobiology of Lp(a) in calcific aortic valve disease. *Expert Rev Cardiovasc Ther* 2017;**15**:797-807. doi:10.1080/14779072.2017.1367286 | | | | |
| 5. Kwiatkowska M, Mickiewicz A, Krzesińska A, Kuchta A, Jankowski M, Gruchała M*, et al.* The Role of Paraoxonase-1 Activity, Apolipoprotein B Levels, and Apolipoprotein B/Apolipoprotein A-I Ratio as Risk Markers for Aortic Stenosis in Patients with a Bicuspid Aortic Valve. *Antioxidants (Basel)* 2025;**14**:167. doi:10.3390/antiox14020167 | | | | |
| 6. Bhatia HS, Dweck MR, Craig N, Capoulade R, Pibarot P, Trainor PJ*, et al.* Oxidized Phospholipids and Calcific Aortic Valvular Disease. *J Am Coll Cardiol* 2024;**84**:2430-2441. doi:10.1016/j.jacc.2024.08.070 | | | | |

**Table S15. The scoring system of diet.**

| Characteristics | Data field ID | Questions | Target Value |
| --- | --- | --- | --- |
| Fruit and Vegetables | 1289 | 1. On average how many heaped tablespoons of COOKED vegetables would you eat per DAY? | Sum ≥ 4.5 pieces or servings /day, |
|  | 1299 | 2. On average how many heaped tablespoons of SALAD or RAW vegetables would you eat per DAY? | Yes (1) / No (0) |
|  | 1309 | 3. About how many pieces of FRESH fruit would you eat per DAY? | 3 tablespoons of vegetables were considered one serving |
| Fish | 1329 | 4. How often do you eat oily fish? (e.g. sardines, salmon, mackerel, herring) | Sum ≥2 intake/week, |
|  | 1339 | 5. How often do you eat other types of fish? (e.g. cod, tinned tuna, haddock) | Yes (1) / No (0) |
| Processed Meat | 1349 | 6. How often do you eat processed meats (such as bacon, ham, sausages, meat pies, kebabs, burgers, chicken nuggets) | ≤ 2 times/week intake of processed meat |
| Red Meat | 1359 | 7. How often do you eat chicken, turkey or other poultry? (Do not count processed meats) | and ≤ 5 times/week of red meat intake , Yes (1) / No (0) |
|  | 1369 | 8. How often do you eat beef? (Do not count processed meats) |  |
|  | 1379 | 9. How often do you eat lamb/mutton? (Do not count processed meats) |  |
|  | 1389 | 10. How often do you eat pork? (Do not count processed meats such as bacon or ham) |  |

**Table S16. Drug classification and data coding.**

| 1. Lipid-lowering medication | 20003;6177;6153 |
| --- | --- |
| Lipid Lowering Drug; Atorvastatin; Velastatin; Rosuvastatin; Simvastatin; Fluvastatin; pravastatin;Eptastatin; lipostat 10mg tablet; Atromid-s 500mg capsule; Modalim 100mg Tablet; crestor 10mg tablet; Bezafibrate; Fenofibrate; Bezafibrate product; Clofibrate; Gemfibrozil; Gemfibrozil product; bezalip; bezalip-mono 400mg m/r tablet; questran 4g/sachet powder; cholestyramine+aspartame 4g/sachet powder; lopid 300 capsule; colestipol; synvinolin; Acipimox; Nicotinic Acid Product; probucol;nicofuranose; lurselle 250mg tablet; colestid 5g/sachet granules; olbetam 250mg capsule; cholestyramine; colestyramine; cholestyramine product; | |
| 1140861922; 1141146234; 1140910654; 1141192410; 1140861958; 1140888594; 1140888648; 1140861970; 1140910632; 140861946;1140862028; 1141192414; 1140861924; 1140861954; 1141157260; 1140861944; 1140861856; 1141157262; 1140861926; 1140861928; 1140861936; 1140861942; 140861858;1140888590;1140910652;1140861868;1140861892;1140861876; 1140861866; 1140861848; 1140861878;1140861894; 1140865576;1140909780; 1141157416; | |
| 2. Antihypertensive medication | 20003;6177;6153 |
| capozide 50mg tablets x28; lisinopril ; carace 2.5mg tablet; zestril 2.5mg tablet; quinapril; accuretic tablet; captopril; acepril 12.5mg tablet; capoten 12.5mg tablet; captopril+hydrochlorothiazide 25mg/12.5mg tablet; innovace 2.5mg tablet; innozide tablet; enalapril maleate+hydrochlorothiazide 20mg/12.5mg tablet; coversyl 2mg tablet; ramipril; staril 10mg tablet; cilazapril; vascace 250micrograms tablet; trandolapril; gopten 500 micrograms capsule; odrik 500micrograms capsule; zestoretic 10 tablet; carace 10 plus tablet; lisinopril+hydrochlorothiazide 10mg/12.5mg tablet; indapamide; amlodipine; accupro 5mg tablet; enalapril; fosinopril; perindopril; felodipine; losartan; cozaar half strength 25mg tablet; perdix 7.5mg tablet; valsartan; diovan 40mg capsule; ecopace 12.5mg tablet; kaplon 12.5mg tablet; losartan potassium+hydrochlorothiazide 10mg/12.5mg tablet; cozaar-comp 50mg/12.5mg tablet; irbesartan; aprovel 75mg tablet; tarka 2mg/180mg m/r capsule; trandolapril+verapamil hydrochloride; candesartan cilexetil; amias 2mg tablet; imidapril hydrochloride; tanatril 5mg tablet; felodipine+ramipril; triapin mite 2.5mg/2.5mg tablet; telmisartan; hyteneze 12.5 tablet; tensopril 12.5mg tablet; capto-co 25mg/12.5mg tablet; pralenal 2.5mg tablet; eprosartan; micardis 20mg tablet; irbesartan+hydrochlorothiazide 150mg/12.5mg tablet; coaprovel 150mg/12.5mg tablet; cozaar 25mg tablet; perindopril+indapamide; coversyl plus 4mg/1.25mg tablet; telmisartan+hydrochlorothiazide 40mg/12.5mg tablet; tritace 1.25mg tablet; caralpha 10/12.5mg tablet; Olmesartan; olmetec 10mg tablet; lopace 2.5mg capsule; ranace 1.25mg capsule; lisicostad hct 10/12.5mg tablet; valsartan+hydrochlorothiazide 80mg/12.5mg tablet; co-diovan 80mg/12.5mg tablet; centyl 2.5mg tablet; urizide 5mg tablet; enduron 5mg tablet; esidrex k tablet; hygroton k tablet combination pack; navidrex-k tablet; serpasil-esidrex tablet; sotalol hydrochloride+hydrochlorothiazide 80mg/12.5mg tablet; metoprolol tartrate+hydrochlorothiazide 100mg/12.5mg tablet; acebutolol+hydrochlorothiazide 200mg/12.5mg tablet; methyldopa+hydrochlorothiazide 250mg/15mg tablet; quinalapril+hydrochlorothiazide 10mg/12.5mg tablet; bisoprolol fumarate+hydrochlorothiazide 10mg/6.25mg tablet; hydroflumethiazide; hydrenox 50mg tablet; mefruside; baycaron 25mg tablet; metolazone; xuret 500micrograms tablet; xipamide; diurexan 20mg tablet; aprinox 2.5mg tablet; berkozide 2.5mg tablet; neo-naclex 5mg tablet; chlorothiazide; saluric 500mg tablet; hygroton 50mg tablet; cyclopenthiazide; navidrex 500mcg tablet; hydrochlorothiazide; esidrex 25mg tablet; hydrosaluric 25mg tablet; amiloride hcl+cyclopenthiazide 2.5mg/250micrograms tablet; centyl k m/r tablet; neo-naclex k m/r tablet; neo-bendromax 2.5mg tablet; nindaxa 2.5mg tablet; chlortalidone; natramid 2.5mg tablet; opumide 2.5mg tablet; diltiazem hcl+hydrochlorothiazide 150mg/12.5mg m/r capsule; natrilix sr 1.5mg m/r tablet; triamterene+chlortalidone 50mg/50mg tablet; atenolol+chlortalidone; diuril 250mg/5ml oral suspension; Bendroflumethiazide; bendroflumethiazide+potassium 2.5mg/7.7mmol m/r tablet; nadolol+bendroflumethiazide 40mg/5mg tablet; timolol maleate+bendroflumethiazide 10mg/2.5mg tablet; atenolol+Bendroflumethiazide; brinaldix k tablet; diatensec 50mg tablet; laractone 25mg tablet; normetic tablet; synuretic tablet; vasetic co-amilozide 5/50mg tablet; pindolol; pindolol+clopamide 10mg/5mg tablet; timolol maleate+co-amilozide 10mg/2.5mg/25mg tablet; aridil 2.5mg/20mg tablet; spiro-co 25mg tablet; midamor 5mg tablet; berkamil 5mg tablet; spiroctan-m 200mg/10ml injection; spironolactone; aldactone 25mg tablet; lasix 20mg tablet; bumetanide; burinex 1mg tablet; spirospare 25mg tablet; spiretic 25mg tablet; spiroctan 25mg tablet; spirolone 25mg tablet; triamterene+benzthiazide 50mg/25mg capsule; triam-co tablet; triamterene+chlorthalidone 50mg/50mg tablet; triamterene+frusemide 50mg/40mg tablet; lasoride tablet; delvas tablet; navispare tablet; amilmaxco 5/50 tablet; burinex a tablet; triamaxco tablet; triamterene; dytac 50mg capsule; aldactide 25 tablet; amil-co tablet; dyazide tablet; dytide capsule; frumil tablet; kalspare tablet; moduret 25 tablet; fru-co tablet; moduretic tablet; amiloride hydrochloride+bumetanide 5mg/1mg tablet; burinex k m/r tablet; diumide-k continus m/r tablet; lasikal m/r tablet; bumetanide+potassium 500micrograms/7.7mmol m/r tablet; diuretic; amiloride; furosemide; amipramizide; zida-co 5mg/50mg tablet; co-triamterzide; co-amilozide; co-flumactone; co-amilofruse; amilamont 5mg/ml s/f oral solution; frusemek 5mg/40mg tablet; atenolol+co-amilozide; angiotensin ii receptor antagonist+diuretic; froop co 5mg/40mg tablet; komil 5/40 tablet; triamterene+furosemide 50mg/40mg tablet; furosemide+potassium 20mg/10mmol m/r tablet; inspra 25mg tablet; spiroprop tablet; metoros 95mg tablet; bedranol 10mg tablet; totamol 25mg tablet; arbralene 50mg tablet; kerlone 20mg tablet; trandate 50mg tablet; betaloc 50mg tablet; mepranix 50mg tablet; metoprolol; artrate+chlorthalidone 100mg/12.5mg tablet; secadrex tablet; sotazide tablet; penbutolol sulphate+frusemide 40mg/20mg tablet; tenoret 50 tablet; tenoretic tablet; tolerzide tablet; viskaldix tablet; timolol maleate+bendrofluazide 10mg/2.5mg tablet; timolol maleate+bendrofluazide 20mg/5mg tablet; atenixco 50mg/12.5mg tablet; beta-adalat capsule; tenif capsule; co-betaloc tablet; inderetic capsule; inderex capsule; kalten capsule; lopresoretic tablet; moducren tablet; prestim tablet; atenolol+nifedipine 50mg/20mg m/r capsule; monocor 5mg tablet; emcor 10mg tablet; celectol 200mg tablet; adalat 5mg capsule; monozide 10 tablet; antipressan 25mg tablet; angilol 10mg tablet; cardinol 10mg tablet; acebutolol; atenolol; tenormin 25 tablet; vasaten 50mg tablet; apsolol 10mg tablet; propanix 10mg tablet; betadur cr 160mg m/r capsule; beta-prograne 160mg m/r capsule; berkolol 10mg tablet; half-betadur cr 80mg m/r capsule; half-inderal la 80mg m/r capsule; half beta-prograne 80mg m/r capsule; inderal 10mg tablet; concordin 5mg tablet; betaxolol; bisoprolol; celiprolol; metoprolol; labetalol; penbutolol; sotalol; timolol; adalate 10mg capsule; avlocardyl retard 160mg m/r capsule; carvedilol; totaretic 50mg/12.5mg tablet; sloprolol 80mg m/r capsule; probeta la 160mg m/r capsule; lopranol la 160mg m/r capsule; atenix 25mg tablet; co-tenidone; atenolol+chlorthalidone; atenolol+bendrofluazide; tenben capsule; half propanix la 80mg m/r capsule; half propatard la 80mg m/r capsule; nebilet 5mg tablet; eucardic 3.125 tablet; dorzolamide+timolol; cardicor 1.25mg tablet; syprol 5mg/5ml oral solution; soloc 5mg tablet; tensomex 100mg tablet; bipranix 5mg tablet; latanoprost+timolol; rapranol sr 80mg m/r capsule; vivacor 5mg tablet calcicard 60mg tablet; lidoflazine; clinium 120mg tablet; vasad 5mg capsule; synadrin 60mg tablet; veractil 25mg tablet; nifedipine; calcilat 10mg capsule; angiopine 5mg capsule; nifensar xl 20mg m/r tablet; coracten sr 10mg m/r capsule; tildiem 60mg m/r tablet; britiazim 60mg m/r tablet; angiozem 60mg m/r tablet; adizem-60 m/r tablet; cardene 20mg capsule; isradipine; istin 5mg tablet; lacidipine; motens 2mg tablet; half securon sr 120mg m/r tablet; securon 40mg tablet; geangin 40mg tablet; berkatens 40mg tablet; cordilox 40mg tablet; parmid 10mg tablet; nimotop 30mg tablet; nimodipine; nicardipine; univer 120mg m/r capsule; verapamil; nifelease 20mg m/r tablet; slozem 120mg m/r capsule; calanif 5mg capsule; angitil sr 90 m/r capsule; adipine mr 10 m/r tablet; kentiazem 60mg m/r capsule; unipine xl 30mg m/r tablet; adizem-xl plus m/r capsule; cardilate mr 10mg m/r tablet; tensipine mr 10 m/r tablet; plendil 2.5mg m/r tablet; nisoldipine; syscor mr 10mg m/r tablet; fortipine la40 m/r tablet; slofedipine 20mg m/r tablet; nifedotard 20mr m/r tablet; verapress mr 240 m/r tablet; viazem xl 120mg m/r capsule; genalat retard 10mg m/r tablet; lercanidipine; zanidip 10mg tablet; mibefradil; calazem 60mg m/r tablet; optil 60mg m/r tablet; dilcardia sr 60mg m/r capsule; nifedipress mr 10 m/r tablet; nivaten retard 10mg m/r tablet; coroday mr 20mg m/r tablet; zemtard 120 xl m/r capsule; ethimil mr 240 m/r tablet; vertab sr 240 m/r tablet; nifopress retard 20mg m/r tablet; zildil sr 60mg m/r capsule; calchan mr 10mg m/r tablet; zemret 180 xl m/r capsule; bi-carzem sr 60mg m/r capsule; horizem sr 90mg m/r capsule; zolvera 40mg/5ml oral solution; disogram sr 60mg m/r capsule; ranvera mr 240mg m/r tablet; cabren 2.5mg m/r tablet; vera-til sr 120mg m/r tablet; kentipine mr 10mg m/r tablet; felotens xl 5mg m/r tablet; felogen xl 5mg m/r tablet; hypolar retard 10mg m/r tablet; valni 20 retard 20mg m/r tablet; cardioplen xl 5mg m/r tablet; amlostin 5mg tablet; neofel xl 5mg m/r tablet; parmid xl 5mg m/r tablet. | |
| 1140851692; 1140860696; 1140860706; 1140860714; 1140860728; 1140860736; 1140860750; 1140860752; 1140860758; 1140860764; 1140860776; 1140860784; 1140860790; 1140860802; 1140860806; 1140860878; 1140860882; 1140860892; 1140860904; 1140860912; 1140860918; 1140864618; 1140864910; 1140864952; 1140866078; 1140879802; 1140881706; 1140888552; 1140888556; 1140888560; 1140888646; 1140916356; 1140916362; 1140923718; 1141145660; 1141145668; 1141150328; 1141150560; 1141151016; 1141151018; 1141152998; 1141153006; 1141153316; 1141153328; 1141156836; 1141156846; 1141164148; 1141164154; 1141165470; 1141165476; 1141166006; 1141167758; 1141167822; 1141170544; 1141170870; 1141171336; 1141172492; 1141172682; 1141172686; 1141179974; 1141180592; 1141180598; 1141187788; 1141188408; 1141190934; 1141193282; 1141193346; 1141199940; 1141200698; 1141200726; 1141201038; 1141201040; 1140851332; 1140851336; 1140851338; 1140851362; 1140851364; 1140851368; 1140851660; 1140860332; 1140860404; 1140860422; 1140860562; 1140860738; 1140864950; 1140866072; 1140866074; 140866084; 1140866086; 1140866092; 1140866096; 1140866108; 1140866110; 1140866128; 1140866132; 1140866136; 1140866138; 1140866140; 1140866146; 1140866156; 1140866158; 1140866162; 1140866164; 1140866168; 1140866422; 1140866440; 1140866446; 1140888918; 1140888922; 1140909706; 1140916870; 1140917068; 1140926778; 1141146378; 1141180772; 1141180778; 1141188636; 1141194794; 1141194800; 1141194804; 1141194808; 1141194810; 1140851360; 1140851418; 1140851420; 1140851428; 1140851430; 1140851436; 1140860292; 1140860322; 1140860336;1140864550; 1140864574; 1140866220; 1140866226; 1140866232; 1140866236; 1140866244; 1140866248; 1140866280; 1140866282; 1140866306; 1140866308; 1140866312; 1140866318; 1140866324; 1140866328; 1140866330; 1140866332; 1140866334; 1140866340; 1140866352; 1140866354; 1140866356; 1140866360; 1140866388; 1140866390; 1140866396; 1140866400; 1140866402; 1140866404; 1140866406; 1140866410; 1140866416; 1140866418; 1140866420; 1140866426; 1140866438; 1140866442; 1140866444; 1140866448; 1140881894; 1140888512; 1140909708; 1140909722; 1140922324; 1140923272; 1140923276; 1140923282; 1140923402; 1140927174; 1140928624; 1141146128; 1141150898; 1141167108; 1141181520; 1141195254; 1141195258; 1141201250; 1140851508; 1140851522; 1140851556; 1140860172; 1140860180; 1140860232; 1140860250; 1140860266; 1140860278; 1140860308; 1140860314; 1140860318; 1140860320; 1140860324; 1140860328; 1140860330; 1140860338; 1140860340; 1140860342; 1140860348; 1140860356; 1140860358; 1140860386; 1140860394; 1140860396; 1140860398; 1140860402; 1140860406; 1140860410; 1140860426; 1140860434; 1140860492; 1140860498; 1140861090; 1140864176; 1140864410; 1140866704; 1140866712; 1140866724; 1140866738; 1140866756; 1140866758; 1140866764; 1140866766; 1140866778; 1140866782; 1140866784; 1140866798; 1140866800; 1140866802; 1140866804; 1140867734; 1140879758; 1140879760; 1140879762; 1140879818; 1140879824; 1140879834; 1140879854; 1140879866; 1140881702; 1140881722; 1140909368; 1140916628; 1140916730; 1140916868; 1140917076; 1140922930; 1140923336; 1141146124; 1141146126; 1141146184; 1141152076; 1141156754; 1141164280; 1141168498; 1141169516; 1141171152; 1141172742; 1141182904; 1141182968; 1141184324; 1141184722; 1141187048; 1141187780; 1140851730; 1140851784; 1140851786; 1140851790; 1140851800; 1140855976; 1140861088; 1140861106; 1140861110; 1140861114; 1140861120; 1140861128; 1140861130; 1140861136; 1140861138; 1140861176; 1140861190; 1140861202; 1140861276; 1140861282; 1140866460; 1140866466; 1140866484; 1140866546; 1140866554; 1140868036; 1140872472; 1140872568; 1140879810; 1140881692; 1140888510; 1140911088; 1140911698; 1140916930; 1140917428; 1140923572; 1140923618; 1140926188; 1140926780; 1140927934; 1140927940; 1140928212; 1140928226; 1140928234; 1141145870; 1141150500; 1141150538; 1141150926; 1141151474; 1141152600; 1141153026; 1141153032; 1141153394; 1141153454; 1141156656; 1141157136; 1141157140; 1141162546; 1141166752; 1141167832; 1141169096; 1141169710; 1141169730; 1141171804; 1141173766; 1141174684; 1141175224; 1141180238; 1141184390; 1141185444; 1141187056; 1141187094; 1141187774; 1141187962; 1141188152; 1141188576; 1141188936; 1141190548; 1141199858; 1141200400; 1141200782; 1141201814 | |
| 3. Antidiabetic medication | 20003; 6177; 6153 |
| Glibornuride; daonil 5mg tablet; glutril 25mg tablet; semi-daonil 2.5mg tablet; glymidine; euglucon 2.5mg tablet; gondafon 500mg tablet ; malix 2.5mg tablet ; pramidex 500mg tablet; diabetamide 2.5mg tablet; glipizide; gliclazide; glibenese 5mg tablet; diamicron 80mg tablet; minodiab 2.5mg tablet; insulin product; tolazamide; metformin; tolanase 100mg tablet; glimepiride; tolbutamide; troglitazone; glyconon 500mg tablet; romozin 200mg tablet ; rastinon 500mg tablet ; amaryl 1mg tablet ; glucophage 500mg tablet; pioglitazone; orabet 500mg tablet; actos 15mg tablet; chlorpropamide; rosiglitazone; diabinese 100mg tablet; avandia 4mg tablet; glymese 250mg tablet; rosiglitazone 1mg / metformin 500mg tablet; glibenclamide; avandamet 1mg / 500mg tablet | |
| 1140857494; 1140874724; 1140857496; 1140874726;1140857500;1140874728; 1140857502; 1140874732; 1140857506; 1140874736; 1140874646; 1140874744; 1140874650; 1140874746; 1140874652; 1140883066; 1140874664; 1140884600; 1140874666; 1141152590; 1140874674; 1141153254; 1140874678; 1141153262; 1140874680; 1141156984; 1140874686; 1141171646; 1140874690; 1141171652; 1140874706; 1141177600; 1140874712; 1141177606; 1140874716; 1141189090; 1140874718; 1141189094; | |
| 4. Antiplatelet medication | 6154; 20003 |
| Aspirin; antiplatelet drug; aspirin; aspirin 75mg tablet; nu-seals aspirin 75mg e/c tablet; aspirin+methocarbamol 325mg/400mg tablet; aspirin+metoclopramide 325mg/5mg effervescent tablet; aspirin+glycine 500mg/133mg dispersible tablet; aspirin+codeine 300mg/8mg tablet; aspirin+codeine; aspirin+papaveretum 500mg/7.71mg dispersible tablet; isosorbide mononitrate+aspirin; dipyridamole+aspirin; disprin cv 100mg m/r tablet; disprin direct dispersible tablet; acetylsalicylic acid; anadin tablet; aspro clear maximum strength soluble tablet; codis dispersible tablet; alka-seltzer tablet; norgesic tablet; persantin 25mg tablet; cerebrovase 25mg tablet; angettes 75mg tablet; aspav dispersible tablet; aspirin+cyclizine hydrochloride 500mg/25mg tablet; sulfinpyrazone; asasantin retard m/r capsule; clopidogrel; plavix 75mg tablet; platet 100mg effervescent tablet；ticlopidine; dipyridamole; pentoxifylline; | |
| 1140861776; 1140868226;1140861806;1140864860;1140868282;1140872040;1140882190;1140882268;1140882392;1141163138;1141164044;1141167844;1140861808;1140882192; 1140909772; 1140911754; 1140909480; 1140856336; 1140917114; 1140856412; 1140861780; 1140861804; 1140868258; 1140882108; 1140909890; 1141167848; 1141168318; 1141168322; 1140861800; 1141163324; 1140861778; 1140909712 | |
| 5. Anticoagulant medication | 20003 |
| Warfarin; Sodium Warfarin; Nicoumalone; Nicoumalone 4mg tablet; Sinthrome 1mg Tablet; Phenindione; Acenocoumarol; | |
| 1140888266; 1140910832; 1140861696; 1140864122; 1140861698; 1140861702; 1140909770; | |
| 6. Antiresorptive medication | 20003 |
| alendronate sodium; risedronate sodium; zoledronic acid; fosamax 5mg tablet; actonel 5mg tablet; disodium etidronate; disodium etidronate+calcium carbonate; ehdp - etidronate disodium; etidronate disodium; sodium clodronate; disodium clodronate; clodronate disodium; disodium pamidronate; disodium tiludronate; | |
| 1140922174; 1141175684;1141173814;1141176570;1141175690;1140868770;1140882980;1140910468;1140913322;1140868794;1140910726;1140910728;1140868784;1140927054 | |
| 7. Vitamin D, Vitamin K supplement | 6155; 20003 |
| Vitamin D; vitamin k product; vitamin a+d capsule; calcium+vitamin d 500units tablet; vitamin d product; vitamin k1; phytomenadione; | |
| 1140871138 ; 1140852766; 1140852948; 1140870954; 1140909886; 1140871146; | |
| 8. Calcium supplement | 6179; 20003 |
| Calcium; calcium+vitamind 500units tablet; calcium salts; calcium-sandoz syrup; calcium carbonate+cholecalciferol 1.25g/5micrograms tablet;calcium+ergocalciferol tablet; disodium etidronate+calcium carbonate; | |
| 1140852948; 1140870788;1140870796;1140871052;1140877630;1140882980; | |

**Table S17. SNP loci included in the construction of the polygenic risk score.**

| Chromosome | Locus | SNP | Effect allele | MAF | Beta | P |
| --- | --- | --- | --- | --- | --- | --- |
| 1 | CELSR2–SORT1 | rs12740374 | T | 0.21 | -0.083381609 | 2.40E-09 |
| 1 | PRRX1 | rs61817383 | T | 0.26 | 0.0861776962410524 | 3.70E-12 |
| 2 | ACTR2 | rs62139062 | T | 0.28 | 0.0769610411361284 | 4.40E-11 |
| 3 | ARHGEF26 | rs6794263 | C | 0.11 | -0.105360515657826 | 1.50E-09 |
| 9 | SMC2 | rs55909255 | C | 0.39 | 0.0676586484738149 | 5.10E-09 |
| 11 | BET1L | rs73386631 | T | 0.043 | 0.148420005118273 | 1.90E-07 |
| 11 | NLRP6 | rs17156153 | T | 0.085 | 0.148420005118273 | 2.60E-13 |
| 13 | HMGB1 | rs181753401 | A | 0.0024 | 0.524728528934982 | 2.10E-07 |
| 16 | TMEM170A | rs11643207 | C | 0.38 | -0.083381608939051 | 5.60E-13 |
| 18 | Intergenic (18q11.2) | rs551520 | T | 0.23 | -0.083381608939051 | 6.80E-11 |
| 1 | ALPL | rs6696066 | A | 0.48 | -0.083381608939051 | 7.10E-14 |
| 1 | PALMD | rs6702619 | G | 0.49 | 0.148420005118273 | 8.70E-43 |
| 1 | NAV1 | rs631556 | A | 0.41 | 0.0861776962410524 | 4.10E-14 |
| 2 | TEX41 | rs7593336 | G | 0.38 | 0.113328685307003 | 1.70E-25 |
| 6 | LPA | rs10455872 | G | 0.069 | 0.371563556432483 | 1.40E-75 |
| 6 | LPA | rs140570886 | C | 0.014 | 0.392042087776024 | 2.30E-17 |
| 7 | IL6 | rs1800797 | A | 0.45 | 0.122217632724249 | 1.90E-27 |
| 11 | FADS1/2 | rs174533 | A | 0.34 | -0.083381608939051 | 2.00E-12 |
|  |  |  |  |  |  |  |
| References: Yu Chen H, Dina C, Small AM, et al. Dyslipidemia, inflammation, calcification, and adiposity in aortic stenosis: a genome-wide study. Eur Heart J. 2023;44(21):1927-1939. doi:10.1093/eurheartj/ehad142 | | | | | | |
|  |  |  |  |  |  |  |

**Table S18. SNP loci included in the validation polygenic risk score analysis.**

| Chromosome | Locus | SNP | Risk Allele | RAF | OR [95% CI] | P |
| --- | --- | --- | --- | --- | --- | --- |
| 1 | PRDM16 | rs2817178 | C | 0.54 | 1.04 [1.03-1.05] | 2.84E-13 |
| 1 | PLEKHM2 | rs112592385 | CT | 0.76 | 1.04 [1.03-1.05] | 4.89E-09 |
| 1 | ALPL | rs12141569 | C | 0.30 | 1.07 [1.06-1.08] | 9.91E-38 |
| 1 | WASF2 | rs77608177 | A | 0.97 | 1.07 [1.05-1.09] | 1.01E-11 |
| 1 | GLIS1 | rs2694594 | C | 0.62 | 1.05 [1.04-1.06] | 3.09E-15 |
| 1 | PCSK9 | rs11591147 | G | 0.99 | 1.21 [1.16-1.26] | 1.10E-17 |
| 1 | DDAH1 | rs11161617 | T | 0.86 | 1.05 [1.03-1.06] | 5.60E-12 |
| 1 | ODF2L | rs76709100 | AT | 0.28 | 1.04 [1.03-1.05] | 1.67E-10 |
| 1 | LMO4 | rs1856047 | A | 0.45 | 1.05 [1.04-1.07] | 1.02E-21 |
| 1 | KYAT3 | rs3806340 | G | 0.48 | 1.03 [1.02-1.05] | 2.20E-10 |
| 1 | PALMD | rs6702619 | G | 0.14 | 1.14 [1.13-1.15] | 1.73E-124 |
| 1 | PSRC1 | rs602633 | G | 0.79 | 1.08 [1.06-1.09] | 7.83E-31 |
| 1 | WARS2 | rs5777384 | GA | 0.38 | 1.04 [1.03-1.05] | 2.25E-10 |
| 1 | CERS2 | rs11205303 | C | 0.24 | 1.05 [1.04-1.07] | 6.94E-21 |
| 1 | IL6R | rs7512646 | G | 0.59 | 1.05 [1.04-1.06] | 5.16E-17 |
| 1 | APCS | rs71628140 | A | 0.03 | 1.1 [1.06-1.14] | 4.37E-08 |
| 1 | PRRX1 | rs17550940 | C | 0.21 | 1.08 [1.06-1.09] | 7.70E-36 |
| 1 | CFHR2 | rs10922149 | T | 0.08 | 1.05 [1.03-1.07] | 2.78E-08 |
| 1 | NAV1 | rs650720 | T | 0.33 | 1.07 [1.06-1.08] | 2.17E-35 |
| 1 | CNTN2 | rs11240351 | G | 0.74 | 1.03 [1.02-1.04] | 3.88E-08 |
| 1 | LYPLAL1 | rs10863444 | T | 0.30 | 1.03 [1.02-1.04] | 5.51E-09 |
| 1 | TP53BP2 | rs1153965 | A | 0.85 | 1.05 [1.04-1.07] | 3.48E-09 |
| 1 | HEATR1 | rs2794763 | T | 0.57 | 1.04 [1.03-1.05] | 1.94E-10 |
| 1 | CEP170 | rs12080886 | T | 0.74 | 1.04 [1.03-1.06] | 1.04E-13 |
| 1 | TSEN15 | rs2274432 | A | 0.35 | 1.03 [1.02-1.04] | 4.93E-08 |
| 2 | TMEM18 | rs2947411 | G | 0.85 | 1.04 [1.03-1.05] | 1.67E-08 |
| 2 | COLEC11 | rs6542680 | C | 0.45 | 1.04 [1.03-1.05] | 3.05E-09 |
| 2 | RNF144A | rs389411 | C | 0.78 | 1.07 [1.06-1.09] | 8.61E-36 |
| 2 | SLC66A3 | rs13018466 | C | 0.45 | 1.04 [1.03-1.05] | 2.63E-12 |
| 2 | TTC32 | rs10184446 | G | 0.45 | 1.03 [1.02-1.05] | 6.94E-10 |
| 2 | APOB | rs5742904 | T | 0.00 | 3.35 [2.46-4.55] | 1.32E-14 |
| 2 | ASXL2 | rs563406543 | G | 0.00 | 1.17 [1.11-1.23] | 9.25E-09 |
| 2 | GCKR | rs780093 | T | 0.42 | 1.03 [1.02-1.04] | 1.50E-08 |
| 2 | STRN | rs2372785 | G | 0.52 | 1.03 [1.02-1.04] | 2.15E-08 |
| 2 | MTA3 | rs71442903 | T | 0.01 | 1.14 [1.09-1.19] | 8.80E-10 |
| 2 | ACTR2 | rs62139061 | C | 0.14 | 1.06 [1.05-1.08] | 4.06E-25 |
| 2 | DYSF | rs6708393 | T | 0.74 | 1.04 [1.03-1.05] | 3.15E-13 |
| 2 | ZEB2 | rs2252641 | C | 0.71 | 1.1 [1.09-1.11] | 4.16E-66 |
| 2 | FAM171B | rs2595382 | G | 0.09 | 1.06 [1.04-1.08] | 2.00E-08 |
| 2 | MFSD6 | rs1278892379 | A | 0.96 | 1.05 [1.03-1.07] | 9.14E-09 |
| 2 | FN1 | rs7608342 | G | 0.22 | 1.04 [1.02-1.05] | 8.05E-10 |
| 2 | TNS1 | rs61741262 | T | 0.94 | 1.06 [1.04-1.08] | 5.72E-11 |
| 2 | NYAP2 | rs2943635 | T | 0.84 | 1.04 [1.02-1.05] | 1.17E-09 |
| 2 | ABCG8 | rs72875462 | C | 0.94 | 1.07 [1.04-1.09] | 1.11E-08 |
| 3 | ATG7 | rs6791877 | A | 0.70 | 1.05 [1.04-1.07] | 9.74E-14 |
| 3 | LRRFIP2 | rs10531920 | TAAAC | 0.76 | 1.03 [1.02-1.04] | 4.90E-09 |
| 3 | FLNB | rs11130602 | G | 0.37 | 1.05 [1.04-1.06] | 1.01E-21 |
| 3 | SLC25A26 | rs62245373 | T | 0.23 | 1.03 [1.02-1.04] | 1.91E-08 |
| 3 | ST3GAL6 | rs828609 | A | 0.32 | 1.03 [1.02-1.04] | 8.59E-09 |
| 3 | UMPS | rs17843768 | A | 0.09 | 1.05 [1.03-1.07] | 1.76E-08 |
| 3 | GATA2 | rs55914222 | C | 0.02 | 1.1 [1.06-1.13] | 3.78E-08 |
| 3 | STAG1 | rs695983 | A | 0.77 | 1.04 [1.03-1.06] | 1.01E-12 |
| 3 | WWTR1 | rs4681529 | A | 0.56 | 1.03 [1.02-1.05] | 1.50E-08 |
| 3 | ARHGEF26 | rs6787361 | G | 0.22 | 1.06 [1.05-1.07] | 2.70E-19 |
| 3 | TRIM59 | rs60460891 | T | 0.65 | 1.03 [1.02-1.04] | 2.76E-09 |
| 3 | MECOM | rs59030006 | T | 0.48 | 1.07 [1.05-1.08] | 4.17E-33 |
| 3 | FNDC3B | rs7637779 | A | 0.51 | 1.04 [1.02-1.05] | 1.78E-10 |
| 3 | ATP13A3 | rs1706003 | G | 0.79 | 1.06 [1.05-1.07] | 3.38E-25 |
| 3 | MUC4 | rs2293232 | T | 0.21 | 1.06 [1.05-1.08] | 7.00E-16 |
| 4 | AFAP1 | rs62289340 | T | 0.16 | 1.07 [1.05-1.08] | 4.29E-32 |
| 4 | SGCB | rs711347 | T | 0.51 | 1.04 [1.03-1.05] | 4.95E-10 |
| 4 | PDGFRA | rs4864857 | C | 0.22 | 1.06 [1.05-1.08] | 2.40E-20 |
| 4 | SHROOM3 | rs10025351 | C | 0.78 | 1.03 [1.02-1.04] | 1.17E-09 |
| 4 | ARHGAP24 | rs12710823 | A | 0.30 | 1.05 [1.04-1.06] | 4.64E-15 |
| 4 | SLC39A8 | rs13107325 | C | 0.99 | 1.07 [1.05-1.1] | 4.75E-10 |
| 4 | LEF1 | rs12502542 | G | 0.34 | 1.06 [1.05-1.07] | 7.08E-26 |
| 4 | MAD2L1 | rs10446728 | T | 0.33 | 1.03 [1.02-1.04] | 2.15E-08 |
| 4 | FAT4 | rs9307578 | G | 0.27 | 1.03 [1.02-1.04] | 5.18E-09 |
| 4 | MAML3 | rs142036542 | T | 0.97 | 1.1 [1.08-1.12] | 5.65E-20 |
| 4 | ZNF827 | rs28702961 | T | 0.68 | 1.03 [1.02-1.05] | 1.15E-09 |
| 4 | PALLD | rs7696431 | T | 0.40 | 1.04 [1.02-1.05] | 1.18E-10 |
| 4 | UGT2B15 | rs451891 | C | 0.54 | 1.04 [1.03-1.05] | 3.21E-08 |
| 4 | RASSF6 | rs17805623 | A | 0.85 | 1.05 [1.03-1.07] | 1.16E-08 |
| 5 | TPPP | rs4990988 | A | 0.29 | 1.05 [1.03-1.06] | 1.37E-09 |
| 5 | DAP | rs12188931 | T | 0.23 | 1.04 [1.02-1.05] | 4.69E-09 |
| 5 | ANKH | rs1061813 | G | 0.26 | 1.03 [1.02-1.04] | 1.70E-08 |
| 5 | ISL1 | rs7713979 | G | 0.30 | 1.04 [1.03-1.05] | 2.28E-13 |
| 5 | HMGCR | rs6871667 | A | 0.53 | 1.03 [1.02-1.04] | 1.84E-08 |
| 5 | RPS23 | rs141825703 | CA | 0.92 | 1.05 [1.04-1.07] | 4.52E-12 |
| 5 | VCAN | rs10060113 | A | 0.00 | 1.16 [1.11-1.22] | 1.24E-11 |
| 5 | FER | rs62361338 | G | 0.87 | 1.05 [1.03-1.06] | 4.63E-13 |
| 5 | CEP120 | rs7725897 | T | 0.30 | 1.05 [1.04-1.06] | 1.66E-13 |
| 5 | FBN2 | rs36716 | G | 0.68 | 1.04 [1.03-1.05] | 1.03E-11 |
| 5 | PDGFRB | rs12523235 | C | 0.83 | 1.04 [1.03-1.05] | 1.14E-08 |
| 5 | EBF1 | rs11135046 | T | 0.35 | 1.05 [1.04-1.06] | 8.03E-17 |
| 5 | NKX2-5 | rs4868244 | A | 0.87 | 1.04 [1.03-1.05] | 5.27E-09 |
| 5 | RGS14 | rs11741640 | G | 0.77 | 1.04 [1.03-1.06] | 3.48E-11 |
| 6 | MYLK4 | rs2449447 | C | 0.56 | 1.04 [1.03-1.05] | 1.55E-10 |
| 6 | ADTRP | rs2294424 | T | 0.47 | 1.04 [1.02-1.05] | 2.39E-08 |
| 6 | PHACTR1 | rs7760527 | G | 0.30 | 1.04 [1.03-1.05] | 8.65E-13 |
| 6 | RNF144B | rs13192536 | T | 0.95 | 1.05 [1.03-1.07] | 5.47E-09 |
| 6 | NOTCH4 | rs3131295 | A | 0.44 | 1.05 [1.04-1.06] | 5.50E-19 |
| 6 | CDKN1A | rs4151702 | C | 0.18 | 1.05 [1.03-1.06] | 9.88E-12 |
| 6 | DST | rs2064592 | T | 0.55 | 1.03 [1.02-1.04] | 3.19E-08 |
| 6 | SLC35F1 | rs11968293 | A | 0.62 | 1.05 [1.04-1.06] | 7.39E-20 |
| 6 | HEY2 | rs1322756 | G | 0.42 | 1.03 [1.02-1.04] | 3.09E-09 |
| 6 | ENPP1 | rs453639 | C | 0.11 | 1.05 [1.04-1.06] | 4.80E-19 |
| 6 | PDE7B | rs6570028 | C | 0.45 | 1.04 [1.03-1.05] | 6.23E-13 |
| 6 | NMBR | rs4895588 | A | 0.29 | 1.05 [1.04-1.07] | 1.74E-17 |
| 6 | ARID1B | rs10499288 | G | 0.16 | 1.07 [1.05-1.09] | 5.41E-11 |
| 6 | LPA | rs74617384 | T | 0.02 | 1.43 [1.4-1.46] | 4.78E-261 |
| 7 | PDGFA | rs9801426 | C | 0.62 | 1.04 [1.03-1.05] | 3.26E-10 |
| 7 | DGKB | rs55734480 | A | 0.25 | 1.04 [1.03-1.06] | 6.46E-12 |
| 7 | POLR1F | rs10279302 | A | 0.34 | 1.06 [1.05-1.07] | 1.22E-24 |
| 7 | IL6 | rs1474347 | C | 0.11 | 1.11 [1.1-1.12] | 1.55E-79 |
| 7 | CREB5 | rs11767370 | G | 0.76 | 1.04 [1.03-1.05] | 9.13E-11 |
| 7 | TBX20 | rs13240826 | C | 0.58 | 1.05 [1.04-1.06] | 2.34E-17 |
| 7 | ELMO1 | rs34370233 | CA | 0.84 | 1.04 [1.03-1.06] | 3.80E-11 |
| 7 | GLI3 | rs17172071 | G | 0.74 | 1.05 [1.04-1.06] | 3.83E-15 |
| 7 | COBL | rs10481025 | G | 0.59 | 1.04 [1.03-1.05] | 1.26E-15 |
| 7 | SPDYE17 | rs4727294 | C | 0.18 | 1.05 [1.03-1.06] | 3.56E-08 |
| 7 | SEMA3C | rs6948442 | A | 0.54 | 1.03 [1.02-1.04] | 3.85E-08 |
| 7 | CDK6 | rs7804293 | T | 0.91 | 1.05 [1.04-1.06] | 4.18E-15 |
| 7 | PODXL | rs75672964 | T | 0.02 | 1.09 [1.06-1.12] | 2.91E-12 |
| 7 | ANLN | rs71553063 | C | 0.41 | 1.03 [1.02-1.04] | 4.55E-08 |
| 8 | MFHAS1 | rs2409091 | A | 0.58 | 1.06 [1.05-1.08] | 3.62E-24 |
| 8 | DLC1 | rs12545594 | C | 0.89 | 1.05 [1.03-1.06] | 5.47E-09 |
| 8 | LPL | rs115849089 | G | 0.88 | 1.07 [1.05-1.08] | 9.96E-14 |
| 8 | NKX2-6 | rs7836730 | C | 0.26 | 1.03 [1.02-1.04] | 9.44E-09 |
| 8 | EBF2 | rs10086575 | G | 0.67 | 1.04 [1.03-1.05] | 2.29E-10 |
| 8 | FAM110B | rs4374979 | G | 0.27 | 1.03 [1.02-1.05] | 3.18E-10 |
| 8 | LRP12 | rs35753933 | C | 0.12 | 1.06 [1.04-1.07] | 2.71E-17 |
| 8 | ZFPM2 | rs7016378 | C | 0.20 | 1.05 [1.03-1.06] | 1.22E-10 |
| 8 | TRIB1 | rs28601761 | C | 0.76 | 1.05 [1.04-1.06] | 1.28E-17 |
| 8 | MYC | rs55950395 | A | 0.95 | 1.07 [1.05-1.09] | 1.42E-16 |
| 8 | PTK2 | rs13438888 | T | 0.72 | 1.05 [1.04-1.06] | 6.36E-19 |
| 8 | PLEC | rs188595907 | G | 0.99 | 1.15 [1.1-1.2] | 1.69E-10 |
| 9 | MTAP | rs3927738 | T | 0.35 | 1.03 [1.02-1.05] | 2.82E-10 |
| 9 | PIP5K1B | rs1412987 | A | 0.91 | 1.03 [1.02-1.05] | 7.36E-09 |
| 9 | PHF2 | rs12553508 | C | 0.53 | 1.04 [1.03-1.05] | 7.74E-11 |
| 9 | SMC2 | rs1253391836 | T | 0.34 | 1.04 [1.03-1.05] | 9.42E-13 |
| 9 | MEGF9 | rs34089221 | T | 0.95 | 1.09 [1.06-1.11] | 9.97E-13 |
| 9 | ABL1 | rs7851994 | C | 0.84 | 1.04 [1.03-1.06] | 9.63E-09 |
| 9 | ABO | rs8176743 | C | 0.83 | 1.06 [1.04-1.08] | 3.21E-09 |
| 9 | HAUS6 | rs10963949 | T | 0.43 | 1.03 [1.02-1.04] | 1.02E-08 |
| 9 | CFAP95 | rs996376 | T | 0.64 | 1.08 [1.05-1.11] | 1.09E-08 |
| 10 | FAM107B | rs36025028 | G | 0.17 | 1.03 [1.02-1.05] | 3.95E-10 |
| 10 | CUBN | rs7905442 | G | 0.45 | 1.04 [1.03-1.05] | 4.32E-14 |
| 10 | LYZL1 | rs10740798 | T | 0.23 | 1.03 [1.02-1.05] | 2.13E-08 |
| 10 | SVIL | rs1571759 | C | 0.57 | 1.04 [1.03-1.05] | 3.43E-12 |
| 10 | JMJD1C | rs7098614 | T | 0.54 | 1.04 [1.03-1.05] | 3.80E-09 |
| 10 | CHST3 | rs1245518 | G | 0.52 | 1.05 [1.04-1.06] | 5.25E-18 |
| 10 | LRMDA | rs11001768 | C | 0.86 | 1.03 [1.02-1.04] | 2.39E-09 |
| 10 | EIF5AL1 | rs71034239 | GCTGTCAACCT | 0.67 | 1.03 [1.02-1.05] | 2.32E-08 |
| 10 | PPP1R3C | rs10881929 | A | 0.15 | 1.03 [1.02-1.05] | 2.20E-09 |
| 10 | HHEX | rs11319879 | T | 0.64 | 1.03 [1.02-1.04] | 1.01E-09 |
| 10 | PLCE1 | rs1223580 | C | 0.31 | 1.04 [1.03-1.05] | 2.35E-15 |
| 10 | SORBS1 | rs140453415 | G | 0.01 | 1.08 [1.05-1.12] | 2.73E-08 |
| 10 | SUFU | rs10786683 | A | 0.64 | 1.04 [1.03-1.05] | 4.23E-14 |
| 10 | STN1 | rs35176048 | CTT | 0.93 | 1.06 [1.04-1.08] | 3.53E-09 |
| 10 | ADRB1 | rs1801253 | C | 0.76 | 1.04 [1.02-1.05] | 9.58E-09 |
| 10 | DLG5 | rs56256440 | G | 0.25 | 1.04 [1.02-1.05] | 4.75E-08 |
| 11 | CIMAP1A | rs11551933 | C | 0.03 | 1.1 [1.08-1.12] | 1.70E-20 |
| 11 | CCDC34 | rs10835169 | C | 0.13 | 1.04 [1.03-1.06] | 4.05E-14 |
| 11 | FADS1 | rs174554 | A | 0.67 | 1.08 [1.07-1.1] | 5.60E-46 |
| 11 | LOC100130987 | rs1427755639 | A | 0.04 | 1.07 [1.05-1.09] | 6.16E-11 |
| 11 | MYEOV | rs10908185 | C | 0.50 | 1.06 [1.05-1.07] | 1.64E-23 |
| 11 | FAM168A | rs28400367 | C | 0.94 | 1.06 [1.04-1.08] | 1.19E-11 |
| 11 | MAML2 | rs12800440 | T | 0.10 | 1.04 [1.02-1.05] | 2.93E-08 |
| 11 | PDGFD | rs2019090 | A | 0.48 | 1.04 [1.03-1.05] | 4.38E-10 |
| 11 | POGLUT3 | rs56393920 | C | 0.06 | 1.04 [1.03-1.06] | 1.13E-08 |
| 11 | ZPR1 | rs964184 | G | 0.26 | 1.06 [1.04-1.07] | 3.79E-13 |
| 11 | SORL1 | rs12280388 | C | 0.06 | 1.1 [1.07-1.12] | 1.66E-18 |
| 11 | OR8A1 | rs143421508 | G | 0.00 | 17.95 [6.87-46.91] | 3.93E-09 |
| 11 | C11orf58 | rs11023993 | G | 0.85 | 1.04 [1.03-1.06] | 3.22E-08 |
| 12 | CACNA1C | rs7314860 | A | 0.19 | 1.05 [1.03-1.06] | 2.05E-10 |
| 12 | FGF23 | rs17773299 | T | 0.87 | 1.1 [1.08-1.12] | 7.32E-28 |
| 12 | CHD4 | rs1639122 | A | 0.46 | 1.03 [1.02-1.04] | 1.02E-08 |
| 12 | PDE3A | rs10770612 | A | 0.71 | 1.08 [1.07-1.1] | 3.73E-32 |
| 12 | BCAT1 | rs10219671 | C | 0.16 | 1.04 [1.03-1.05] | 1.70E-09 |
| 12 | TNS2 | rs73099903 | T | 0.06 | 1.07 [1.04-1.09] | 1.37E-10 |
| 12 | HMGA2 | rs66980761 | A | 0.90 | 1.09 [1.07-1.1] | 3.41E-32 |
| 12 | SLC6A15 | rs11116362 | T | 0.68 | 1.03 [1.02-1.05] | 7.82E-10 |
| 12 | ATP2B1 | rs57481061 | C | 0.78 | 1.05 [1.03-1.06] | 4.18E-09 |
| 12 | CEP83 | rs73372224 | G | 0.97 | 1.12 [1.1-1.14] | 1.13E-27 |
| 12 | CMKLR1 | rs141421422 | A | 1.00 | 1.26 [1.16-1.36] | 1.65E-08 |
| 12 | PPTC7 | rs1265744 | T | 0.48 | 1.05 [1.03-1.06] | 1.28E-15 |
| 12 | HNF1A | rs1169288 | C | 0.42 | 1.05 [1.04-1.06] | 8.55E-18 |
| 12 | TMED2 | rs137861315 | C | 0.02 | 1.09 [1.06-1.12] | 2.08E-08 |
| 12 | SCARB1 | rs10846744 | C | 0.53 | 1.08 [1.07-1.1] | 8.70E-27 |
| 13 | SGCG | rs7987944 | T | 0.80 | 1.03 [1.02-1.05] | 2.80E-09 |
| 13 | CDK8 | rs12430162 | T | 0.35 | 1.03 [1.02-1.05] | 3.44E-10 |
| 13 | SMAD9 | rs556429 | A | 0.12 | 1.05 [1.03-1.06] | 1.60E-12 |
| 13 | SPRY2 | rs7337769 | A | 0.79 | 1.03 [1.02-1.04] | 2.64E-08 |
| 13 | GPC6 | rs4366594 | G | 0.24 | 1.04 [1.03-1.05] | 8.54E-12 |
| 13 | FARP1 | rs11617458 | A | 0.87 | 1.04 [1.03-1.05] | 1.12E-09 |
| 13 | COL4A2 | rs3803236 | C | 0.42 | 1.03 [1.02-1.04] | 3.74E-08 |
| 14 | PRMT5 | rs8006409 | G | 0.68 | 1.03 [1.02-1.04] | 1.75E-08 |
| 14 | NFATC4 | rs2229309 | C | 0.27 | 1.03 [1.02-1.04] | 3.36E-08 |
| 14 | DAAM1 | rs1957406 | A | 0.46 | 1.03 [1.02-1.04] | 2.54E-08 |
| 14 | SIPA1L1 | rs61989383 | C | 0.11 | 1.05 [1.04-1.06] | 4.22E-14 |
| 14 | DLST | rs10483863 | G | 0.53 | 1.03 [1.02-1.04] | 8.78E-10 |
| 14 | MARK3 | rs12185021 | G | 0.26 | 1.03 [1.02-1.05] | 4.65E-09 |
| 14 | MTA1 | rs112766772 | C | 0.11 | 1.05 [1.04-1.07] | 1.79E-13 |
| 14 | ZNF219 | rs7149011 | C | 0.63 | 1.03 [1.02-1.05] | 3.02E-08 |
| 15 | STARD9 | rs9919994 | G | 0.77 | 1.07 [1.06-1.09] | 1.23E-28 |
| 15 | ALDH1A2 | rs6493981 | T | 0.70 | 1.05 [1.04-1.06] | 3.05E-20 |
| 15 | SMAD3 | rs17228212 | T | 0.93 | 1.04 [1.02-1.05] | 4.11E-09 |
| 15 | ADAMTS7 | rs4243085 | G | 0.32 | 1.06 [1.05-1.07] | 1.08E-21 |
| 15 | ADAMTS7P1 | rs757871046 | A | 0.29 | 1.08 [1.05-1.11] | 3.61E-08 |
| 15 | AKAP13 | rs716401 | C | 0.45 | 1.03 [1.02-1.04] | 5.92E-09 |
| 15 | ACAN | rs12594617 | C | 0.72 | 1.06 [1.05-1.07] | 4.43E-17 |
| 15 | LYSMD4 | rs60232676 | CTG | 0.59 | 1.03 [1.02-1.04] | 2.23E-08 |
| 15 | PCSK6 | rs4246336 | A | 0.59 | 1.03 [1.02-1.05] | 1.99E-08 |
| 15 | FES | rs1573643 | C | 0.33 | 1.03 [1.02-1.05] | 3.19E-08 |
| 16 | ANKS3 | rs859327 | A | 0.22 | 1.04 [1.03-1.06] | 1.70E-08 |
| 16 | FTO | rs56094641 | G | 0.24 | 1.05 [1.04-1.07] | 7.71E-22 |
| 16 | BCAR1 | rs12933281 | G | 0.55 | 1.07 [1.06-1.08] | 4.28E-34 |
| 16 | MAF | rs28581385 | T | 0.19 | 1.04 [1.03-1.06] | 1.92E-08 |
| 16 | ZFPM1 | rs36049560 | A | 0.45 | 1.04 [1.03-1.06] | 2.01E-12 |
| 17 | SMG6 | rs2126202 | A | 0.62 | 1.03 [1.02-1.04] | 2.57E-09 |
| 17 | RAP1GAP2 | rs715661 | T | 0.24 | 1.05 [1.03-1.06] | 1.40E-12 |
| 17 | TNFSF12 | rs11654281 | T | 0.11 | 1.08 [1.06-1.11] | 1.01E-10 |
| 17 | MYOCD | rs11078075 | C | 0.33 | 1.04 [1.03-1.06] | 1.49E-10 |
| 17 | SREBF1 | rs9899634 | T | 0.77 | 1.04 [1.03-1.05] | 3.26E-11 |
| 17 | SARM1 | rs7212510 | T | 0.32 | 1.04 [1.03-1.05] | 3.44E-13 |
| 17 | BRCA1 | rs8176215 | T | 0.35 | 1.04 [1.03-1.06] | 4.36E-15 |
| 17 | RNFT1 | rs62083666 | A | 0.68 | 1.04 [1.03-1.06] | 6.01E-10 |
| 17 | TBX4 | rs744651 | C | 0.73 | 1.04 [1.02-1.05] | 7.83E-09 |
| 17 | MRC2 | rs2465417 | C | 0.29 | 1.04 [1.03-1.05] | 9.36E-11 |
| 17 | APOH | rs78357146 | G | 0.02 | 1.13 [1.09-1.17] | 1.26E-12 |
| 17 | KCNJ2 | rs7225126 | A | 0.78 | 1.03 [1.02-1.05] | 1.41E-09 |
| 17 | SDK2 | rs113977592 | C | 0.94 | 1.07 [1.05-1.1] | 2.56E-08 |
| 17 | SOCS3 | rs7221341 | T | 0.18 | 1.04 [1.03-1.05] | 8.51E-11 |
| 17 | RNF213 | rs9674961 | G | 0.55 | 1.03 [1.02-1.05] | 7.50E-09 |
| 17 | BAHCC1 | rs11870740 | A | 0.76 | 1.07 [1.05-1.08] | 2.22E-21 |
| 17 | NF1 | rs17883335 | C | 0.00 | 2.71 [1.92-3.83] | 1.65E-08 |
| 17 | CDC27 | rs117778193 | T | 0.93 | 1.07 [1.04-1.09] | 2.00E-08 |
| 17 | TMEM100 | rs117573680 | T | 0.00 | 2.05 [1.58-2.64] | 4.14E-08 |
| 18 | CTAGE1 | rs636261 | T | 0.93 | 1.06 [1.04-1.07] | 3.90E-18 |
| 18 | CDH2 | rs12458840 | G | 0.90 | 1.04 [1.03-1.05] | 5.97E-11 |
| 18 | LOC105372043 | rs1621375 | A | 0.35 | 1.04 [1.03-1.05] | 9.39E-11 |
| 18 | NFATC1 | rs454854 | G | 0.56 | 1.04 [1.03-1.05] | 7.16E-10 |
| 19 | ANGPTL4 | rs116843064 | G | 0.99 | 1.2 [1.16-1.25] | 2.16E-21 |
| 19 | S1PR2 | rs2033493 | A | 0.30 | 1.04 [1.03-1.06] | 2.96E-10 |
| 19 | LDLR | rs6511720 | G | 0.95 | 1.07 [1.05-1.09] | 5.77E-16 |
| 19 | KLF2 | rs1840828 | T | 0.48 | 1.04 [1.03-1.05] | 4.44E-11 |
| 19 | COMP | rs12974746 | G | 0.01 | 1.14 [1.1-1.18] | 9.18E-15 |
| 19 | LTBP4 | rs112009052 | A | 0.00 | 1.16 [1.12-1.22] | 3.00E-12 |
| 19 | HAS1 | rs10417548 | A | 0.11 | 1.04 [1.03-1.06] | 1.40E-11 |
| 19 | SCAF1 | rs76387513 | T | 0.03 | 1.08 [1.05-1.11] | 4.16E-08 |
| 20 | HAO1 | rs11700260 | C | 0.09 | 1.07 [1.06-1.09] | 1.59E-18 |
| 20 | JAG1 | rs4813967 | C | 0.53 | 1.03 [1.02-1.04] | 2.03E-09 |
| 20 | NOL4L | rs4911238 | T | 0.58 | 1.04 [1.02-1.05] | 1.56E-08 |
| 20 | TP53INP2 | rs541427922 | C | 0.88 | 1.05 [1.03-1.07] | 7.78E-11 |
| 20 | CYP24A1 | rs35870583 | G | 0.86 | 1.06 [1.05-1.08] | 4.47E-16 |
| 20 | RBM38 | rs34161672 | A | 0.20 | 1.03 [1.02-1.04] | 2.33E-08 |
| 20 | ZNF512B | rs2275294 | A | 0.43 | 1.04 [1.03-1.05] | 1.48E-09 |
| 21 | ADAMTS5 | rs151058 | C | 0.84 | 1.05 [1.03-1.06] | 2.48E-10 |
| 21 | MAP3K7CL | rs2832230 | G | 0.82 | 1.05 [1.03-1.07] | 7.91E-11 |
| 21 | ERG | rs117870289 | T | 0.00 | 1.24 [1.18-1.3] | 1.06E-17 |
| 22 | CCDC188 | rs175183 | T | 0.54 | 1.04 [1.03-1.05] | 1.28E-09 |
| 22 | ASCC2 | rs16988381 | A | 0.03 | 1.14 [1.1-1.18] | 1.49E-15 |
| 22 | SYN3 | rs35659812 | AGGC | 0.99 | 1.15 [1.11-1.19] | 1.64E-16 |
| 22 | TRIOBP | rs1129448 | A | 0.55 | 1.05 [1.04-1.07] | 1.36E-21 |
| 22 | CYP2D6 | rs1176281218 | G | 0.78 | 1.05 [1.04-1.07] | 1.92E-14 |
| 22 | FAM118A | rs6006988 | T | 0.52 | 1.04 [1.03-1.06] | 3.68E-13 |
|  |  |  |  |  |  |  |
| Small AM, Yang TY, Itoh S, et al. Genomic and transcriptomic analyses of aortic stenosis enhance therapeutic target discovery and disease prediction. Nat Genet. 2026;58(1):57-66. doi:10.1038/s41588-025-02417-6 | | | | | | |
|  |  |  |  |  |  |  |

**Table S19. Missing percentage and reason for variables in primary cohort.**

| **Class** | **Variable** | **Missing Percentage (%)** | **Missing reason** | **Type** |
| --- | --- | --- | --- | --- |
| **Social factors** | Age at UKB recruitment | 0 | — | Continuous |
|  | Sex | 0 | — | Dichotomous |
|  | Ethnicity | 0.45 | Do not know / Prefer not to answer | Categorical |
|  | Townsend deprivation index | 0.12 | — | Continuous |
|  | Education | 18.65 | Prefer not to answer | Dichotomous |
| **Health behaviors** | Smoking status | 0.47 | Prefer not to answer | Ordinal |
|  | Alcohol intake frequency | 0.2 | Prefer not to answer | Ordinal |
|  | Cooked vegetable intake | 1.41 | Do not know / Prefer not to answer | Continuous |
|  | Salad/raw vegetable intake | 1.45 | Do not know / Prefer not to answer | Continuous |
|  | Fresh fruit intake | 0.54 | Do not know / Prefer not to answer | Continuous |
|  | Oily fish intake | 0.71 | Do not know / Prefer not to answer | Ordinal |
|  | Non oily fish intake | 0.62 | Do not know / Prefer not to answer | Ordinal |
|  | Processed meat intake | 0.34 | Do not know / Prefer not to answer | Ordinal |
|  | Poultry intake | 0.31 | Do not know / Prefer not to answer | Ordinal |
|  | Beef intake | 0.6 | Do not know / Prefer not to answer | Ordinal |
|  | Lamb/mutton intake | 0.8 | Do not know / Prefer not to answer | Ordinal |
|  | Pork intake | 0.75 | Do not know / Prefer not to answer | Ordinal |
|  | Sleep time | 0.74 | Do not know / Prefer not to answer | Continuous |
|  | Time spent using computer | 1.02 | — | Continuous |
|  | Time spent watching television (TV) | 0.99 | — | Continuous |
|  | MET minutes per week for vigorous activity | 22.78 | — | Continuous |
|  | MET minutes per week for moderate activity | 22.78 | — | Continuous |
|  | MET minutes per week for walking | 22.78 | — | Continuous |
| **Physical measures and biomarkers** | Systolic blood pressure | 5.78 | — | Continuous |
|  | Diastolic blood pressure | 5.78 | — | Continuous |
|  | Body mass index | 0 | — | Continuous |
|  | C-reactive protein | 4.54 | — | Continuous |
|  | Creatinine | 4.38 | — | Continuous |
|  | HDL cholesterol | 12.33 | — | Continuous |
|  | LDL direct | 4.52 | — | Continuous |
|  | Lipoprotein(a) | 23.56 | — | Continuous |
|  | Triglycerides | 4.41 | — | Continuous |
|  | Total bilirubin | 4.73 | — | Continuous |
|  | Direct bilirubin | 18.78 | — | Continuous |
|  | Apolipoprotein A | 12.81 | — | Continuous |
|  | Apolipoprotein B | 4.81 | — | Continuous |
|  | HbA1c | 4.71 | — | Continuous |
|  | Calcium | 12.33 | — | Continuous |
|  | Phosphate | 12.45 | — | Continuous |
|  | Urate | 4.46 | — | Continuous |
|  | Urea | 4.41 | — | Continuous |
| **Medications and supplements** | Lipid-lowering medication | 0 | — | Dichotomous |
|  | Antihypertensive medication | 0 | — | Dichotomous |
|  | Antidiabetic medication | 0 | — | Dichotomous |
|  | Antiplatelet medication | 0 | — | Dichotomous |
|  | Anticoagulant medication | 0 | — | Dichotomous |
|  | Antiresorptive medication | 0 | — | Dichotomous |
|  | Vitamin supplement | 0 | — | Dichotomous |
|  | Calcium supplement | 0 | — | Dichotomous |
| **Outcomes** | AVS event | 0 | — | Dichotomous |
|  | AVR event | 0 | — | Dichotomous |
|  | MVR event | 0 | — | Dichotomous |
|  | AVS event date | 0 | — | Continuous |
|  | AVR event date | 0 | — | Continuous |
|  | MVR event date | 0 | — | Continuous |

**Table S20. Missing percentage and reason for variables in proteomic cohort.**

| **Class** | **Variable** | **Missing Percentage (%)** | **Missing reason** | **Type** |
| --- | --- | --- | --- | --- |
| **Social factors** | Age at UKB recruitment | 0 | — | Continuous |
|  | Sex | 0 | — | Dichotomous |
|  | Ethnicity | 0.48 | Do not know / Prefer not to answer | Categorical |
|  | Townsend deprivation index | 0.12 | — | Continuous |
|  | Education | 18.88 | Prefer not to answer | Dichotomous |
| **Health behaviors** | Smoking status | 0.48 | Prefer not to answer | Ordinal |
|  | Alcohol intake frequency | 0.23 | Prefer not to answer | Ordinal |
|  | Cooked vegetable intake | 1.55 | Do not know / Prefer not to answer | Continuous |
|  | Salad/raw vegetable intake | 1.61 | Do not know / Prefer not to answer | Continuous |
|  | Fresh fruit intake | 0.62 | Do not know / Prefer not to answer | Continuous |
|  | Oily fish intake | 0.84 | Do not know / Prefer not to answer | Ordinal |
|  | Non oily fish intake | 0.8 | Do not know / Prefer not to answer | Ordinal |
|  | Processed meat intake | 0.41 | Do not know / Prefer not to answer | Ordinal |
|  | Poultry intake | 0.36 | Do not know / Prefer not to answer | Ordinal |
|  | Beef intake | 0.66 | Do not know / Prefer not to answer | Ordinal |
|  | Lamb/mutton intake | 0.9 | Do not know / Prefer not to answer | Ordinal |
|  | Pork intake | 0.87 | Do not know / Prefer not to answer | Ordinal |
|  | Sleep time | 0.87 | Do not know / Prefer not to answer | Continuous |
|  | Time spent using computer | 1.22 | — | Continuous |
|  | Time spent watching television (TV) | 1.12 | — | Continuous |
|  | MET minutes per week for vigorous activity | 23.04 | — | Continuous |
|  | MET minutes per week for moderate activity | 23.04 | — | Continuous |
|  | MET minutes per week for walking | 23.04 | — | Continuous |
| **Physical measures and biomarkers** | Systolic blood pressure | 6.07 | — | Continuous |
|  | Diastolic blood pressure | 6.07 | — | Continuous |
|  | Body mass index | 0 | — | Continuous |
|  | C-reactive protein | 4.74 | — | Continuous |
|  | Creatinine | 4.49 | — | Continuous |
|  | HDL cholesterol | 12.5 | — | Continuous |
|  | LDL direct | 4.66 | — | Continuous |
|  | Lipoprotein(a) | 23.68 | — | Continuous |
|  | Triglycerides | 4.52 | — | Continuous |
|  | Total bilirubin | 4.82 | — | Continuous |
|  | Direct bilirubin | 18.98 | — | Continuous |
|  | Apolipoprotein A | 12.99 | — | Continuous |
|  | Apolipoprotein B | 4.99 | — | Continuous |
|  | HbA1c | 5 | — | Continuous |
|  | Calcium | 12.45 | — | Continuous |
|  | Phosphate | 12.65 | — | Continuous |
|  | Urate | 4.58 | — | Continuous |
|  | Urea | 4.52 | — | Continuous |
| **Medications and supplements** | Lipid-lowering medication | 0 | — | Dichotomous |
|  | Antihypertensive medication | 0 | — | Dichotomous |
|  | Antidiabetic medication | 0 | — | Dichotomous |
|  | Antiplatelet medication | 0 | — | Dichotomous |
|  | Anticoagulant medication | 0 | — | Dichotomous |
|  | Antiresorptive medication | 0 | — | Dichotomous |
|  | Vitamin supplement | 0 | — | Dichotomous |
|  | Calcium supplement | 0 | — | Dichotomous |
| **Outcomes** | AVS event | 0 | — | Dichotomous |
|  | AVR event | 0 | — | Dichotomous |
|  | MVR event | 0 | — | Dichotomous |
|  | AVS event date | 0 | — | Continuous |
|  | AVR event date | 0 | — | Continuous |
|  | MVR event date | 0 | — | Continuous |

**Table S21. ICD codes and Field ID defining diseases in UKB.**

| **Disease** | **Data field ID** | **ICD-10** |
| --- | --- | --- |
| **Valvular heart disease** | | |
| **Rheumatic valve disease** |  |  |
| Mitral valve | 131276 | I05.0; I05.1; I05.2; I05.8; I05.9; |
| Aortic valve | 131278 | I06.0; I06.1; I06.2; I06.8; I06.9; |
| Tricuspid valve | 131280 | I07.0; I07.1; I07.2; I07.8; I07.9; |
| **Congenital valve disease or Marfan's syndrome** |  |  |
| Pulmonary and tricuspid valves | 132470 | Q22.1; Q22.2; Q22.4; Q22.5; Q22.8; Q22.9; |
| Aortic and mitral valves | 132472 | Q23.0; Q23.1; Q23.2; Q23.3; Q23.4; Q23.8; Q23.9; |
| Marfan's syndrome | 41270 | Q87.4 |
| **Endocarditis with Valvular Heart Disease** |  |  |
| Valve unspecified | 131330 | I38 |
| Heart valve disorders in diseases classified elsewhere | 131332 | I39.0; I39.1; I39.3; I39.4; I39.8; |
| **Nonrheumatic heart diseases** |  |  |
| Mitral valve disorders | 131322 | I34.0; I34.1; I34.2; I34.8; I34.9; |
| Aortic valve disorders | 131324 | I35.0; I35.1; I35.2; I35.8; I35.9; |
| Tricuspid valve disorders | 131326 | I36.0; I36.1; I36.2; I36.8; I36.9; |
| Pulmonary valve disorders | 131328 | I37.0; I37.1; I37.2; I37.8; I37.9; |
| Multiple valve disorders | 131382 | I08.0; I08.1; I08.2; I08.3; I08.8; I08.9; |
| **Desease outcome: occurrence of degenerative valvular heart disease (ICD-10 codes from hospital episode records or death registers)** | | |
| Aortic valve stenosis | 41270 | I35.0; I35.2; |
| Aortic valve regurgitation | 41270 | I35.1 |
| Mitral valve regurgitation | 41270 | I34.0 |
| Note: ICD: international classification disease. | | |

**Table S22. Comparative analysis of the clinical performance of three different predictive models.**

| **Cox regression** | | | **LightGBM** | | | **XGBoost** | | |
| --- | --- | --- | --- | --- | --- | --- | --- | --- |
| Disease(trait) | C-index for train | C-index for test | Disease(trait) | C-index for train | C-index for test | Disease(trait) | C-index for train | C-index for test |
| Valvular heart disease | 0.7661(0.7645, 0.7678) | 0.7711(0.7658, 0.7763) | Valvular heart disease | 0.7682 (0.7665, 0.7699) | 0.7695 (0.7642, 0.7749) | Valvular heart disease | 0.7671 (0.7654, 0.7687) | 0.7681 (0.7628, 0.7734) |
| Aortic valve stenosis | 0.8139(0.8124, 0.8155) | 0.8065(0.8015, 0.8114) | Aortic valve stenosis | 0.7998 (0.7972, 0.8024) | 0.7893 (0.7842, 0.7945) | Aortic valve stenosis | 0.8080 (0.8065, 0.8096) | 0.7834 (0.7782, 0.7886) |
| Mitral valve regurgitation | 0.7428(0.7410, 0.7445) | 0.7477(0.7423, 0.7532) | Mitral valve regurgitation | 0.7401 (0.7384, 0.7419) | 0.7389 (0.7334, 0.7444) | Mitral valve regurgitation | 0.7343 (0.7325, 0.7360) | 0.7340 (0.7284, 0.7395) |
| Aortic valve regurgitation | 0.7517(0.7500, 0.7534) | 0.7504(0.7449, 0.7558) | Aortic valve regurgitation | 0.7254 (0.7236, 0.7272) | 0.7088 (0.7028, 0.7148) | Aortic valve regurgitation | 0.7234 (0.7217, 0.7252) | 0.7758 (0.7706, 0.7811) |
